# Mapping the global prevalence, incidence, and mortality of *Plasmodium falciparum*, 2000–17: a spatial and temporal modelling study

**DOI:** 10.1016/S0140-6736(19)31097-9

**Published:** 2019-07-27

**Authors:** Daniel J Weiss, Tim C D Lucas, Michele Nguyen, Anita K Nandi, Donal Bisanzio, Katherine E Battle, Ewan Cameron, Katherine A Twohig, Daniel A Pfeffer, Jennifer A Rozier, Harry S Gibson, Puja C Rao, Daniel Casey, Amelia Bertozzi-Villa, Emma L Collins, Ursula Dalrymple, Naomi Gray, Joseph R Harris, Rosalind E Howes, Sun Yun Kang, Suzanne H Keddie, Daniel May, Susan Rumisha, Michael P Thorn, Ryan Barber, Nancy Fullman, Chantal K Huynh, Xie Kulikoff, Michael J Kutz, Alan D Lopez, Ali H Mokdad, Mohsen Naghavi, Grant Nguyen, Katya Anne Shackelford, Theo Vos, Haidong Wang, David L Smith, Stephen S Lim, Christopher J L Murray, Samir Bhatt, Simon I Hay, Peter W Gething

**Affiliations:** a Malaria Atlas Project, Big Data Institute, Li Ka Shing Centre for Health Information and Discovery, University of Oxford, Oxford, UK; b Global Health Division, Research Triangle Institute International, Washington, DC, USA; c Public Health Division, School of Medicine, University of Nottingham, Nottingham, UK; d Menzies School of Health Research, Charles Darwin University, Casuarina, NT, Australia; e Seattle and King County Public Health, Seattle, WA, USA; f Institute for Disease Modeling, Bellevue, WA, USA; g Public Health England, Department of Health and Social Care, London, UK; h Instruct: An Integrated Structural Biology Infrastructure for Europe, Oxford, UK; i Institute for Health Metrics and Evaluation, University of Washington, Seattle, WA, USA; j Imperial College London, London, UK

## Abstract

**Background:**

Since 2000, the scale-up of malaria control interventions has substantially reduced morbidity and mortality caused by the disease globally, fuelling bold aims for disease elimination. In tandem with increased availability of geospatially resolved data, malaria control programmes increasingly use high-resolution maps to characterise spatially heterogeneous patterns of disease risk and thus efficiently target areas of high burden.

**Methods:**

We updated and refined the *Plasmodium falciparum* parasite rate and clinical incidence models for sub-Saharan Africa, which rely on cross-sectional survey data for parasite rate and intervention coverage. For malaria endemic countries outside of sub-Saharan Africa, we produced estimates of parasite rate and incidence by applying an ecological downscaling approach to malaria incidence data acquired via routine surveillance. Mortality estimates were derived by linking incidence to systematically derived vital registration and verbal autopsy data. Informed by high-resolution covariate surfaces, we estimated *P falciparum* parasite rate, clinical incidence, and mortality at national, subnational, and 5 × 5 km pixel scales with corresponding uncertainty metrics.

**Findings:**

We present the first global, high-resolution map of *P falciparum* malaria mortality and the first global prevalence and incidence maps since 2010. These results are combined with those for *Plasmodium vivax* (published separately) to form the malaria estimates for the Global Burden of Disease 2017 study. The *P falciparum* estimates span the period 2000–17, and illustrate the rapid decline in burden between 2005 and 2017, with incidence declining by 27·9% and mortality declining by 42·5%. Despite a growing population in endemic regions, *P falciparum* cases declined between 2005 and 2017, from 232·3 million (95% uncertainty interval 198·8–277·7) to 193·9 million (156·6–240·2) and deaths declined from 925 800 (596 900–1 341 100) to 618 700 (368 600–952 200). Despite the declines in burden, 90·1% of people within sub-Saharan Africa continue to reside in endemic areas, and this region accounted for 79·4% of cases and 87·6% of deaths in 2017.

**Interpretation:**

High-resolution maps of *P falciparum* provide a contemporary resource for informing global policy and malaria control planning, programme implementation, and monitoring initiatives. Amid progress in reducing global malaria burden, areas where incidence trends have plateaued or increased in the past 5 years underscore the fragility of hard-won gains against malaria. Efforts towards elimination should be strengthened in such areas, and those where burden remained high throughout the study period.

**Funding:**

Bill & Melinda Gates Foundation.

## Introduction

The global burden of *Plasmodium falciparum* malaria has declined substantially since 2000,^1^, ^2^ but declines have not been universal and areas of high burden persist in many countries. Previous research attributed declines in *P falciparum* burden to the scale-up of malaria control interventions,^1^ while indirect factors such as expanded access to improved health care and increasing levels of urbanisation have also contributed to *P falciparum* parasite rate declines.^3^ The confluence of historical *P falciparum* burden, environmental suitability for transmission, and varying levels of malaria control results in complex spatiotemporal patterns of prevalence, incidence, and mortality.

Establishing the spatiotemporal patterns in global malaria maps is necessary to contextualise changes in burden relative to shifting disease control policy, funding, and implementation. Here, we present global maps of *P falciparum* prevalence in children aged 2–10 years, all-age incidence, and all-age mortality at a 5 × 5 km spatial resolution annually for 2000–17. To the best of our knowledge, the resulting maps are the first high-spatial-resolution assessment of *P falciparum* malaria at the global scale since 2010,^4^ the first global maps of *P falciparum* mortality, and the first time any of these pixel-level metrics have been modelled through time at the global scale. The maps show the spatially varying progress towards reducing malaria and form a basis for assessing the ongoing effectiveness of malaria control in the context of efforts towards elimination, and provide an evidence base for directing effort and interventions to areas of greatest need.

**Research in context**

**Evidence before this study**

Mapping malaria transmission intensity and the distribution of cases has been the cornerstone to many countries' control programmes and elimination strategies, including using malaria maps to inform intervention targeting and implementation. The Malaria Atlas Project (MAP) developed methods to synthesise geospatially resolved malaria data and predictors of malaria transmission into maps of *Plasmodium falciparum* endemicity; however, previous global maps have focused on a single year and did not include routine surveillance data for modelling burden. More recent studies from 2015 and 2016, extended these methods to produce 5 × 5 km maps of *P falciparum* burden and mortality in sub-Saharan Africa for 2000–15, while also assessing the contribution of key malaria interventions toward burden declines. To date, however, no global temporally dynamic *P falciparum* maps exist at high spatial resolution. Global epidemiological endeavours such as the Global Burden of Diseases, Injuries, and Risk Factors Study (GBD) provide some subnational estimates of *P falciparum* morbidity and mortality, while many national malaria control programmes monitor malaria deaths and cases at the first or second administrative level.

**Added value of this study**

Drawing from the geostatistical methods established by the MAP and the broader GBD 2017 study, we generated the first-ever, high-resolution, temporally dynamic, global maps of *P falciparum* incidence, prevalence, and mortality for 106 endemic countries for 2000–17. Since the MAP's 2010 *P falciparum* map was published, our database of *P falciparum* parasite rate points has expanded substantially and now includes 43 187 points within sub-Saharan Africa for the years 2000–17. These points underpin the burden estimates for sub-Saharan Africa, but outside Africa routine surveillance case reports are far more common than *P falciparum* parasite rate points. As such, we collected 69 120 routine reports at a range of administrative levels and standardised each using protocols defined by WHO. To optimally use these differing data types, we developed a two-pronged malaria estimation strategy. The first approach was termed the cartographic method, whereby we updated input datasets to produce new results for 36 countries in sub-Saharan Africa. The second approach is known as the surveillance method, in which we used routine malaria surveillance reports to define national-level burden estimates and then applied an ecological downscaling model to produce pixel-level estimates. We modelled malaria mortality by updating an established approach in sub-Saharan Africa that relies on estimating pixel-level case fatality rates. For malaria endemic countries outside of sub-Saharan Africa, we produced mortality estimates by drawing on the broader GBD study's cause-of-death estimation approach. Based on the 5 × 5 km pixel gridded estimates, we aggregated each malaria metric to first and second administrative units, national, regional, and global levels to provide the full range of geospatial resolution that decision makers might require. These outputs provide the information necessary for assessing levels and trends in malaria at more granular geographical units while using a consistent methodological framework that allows for cross-border comparisons.

**Implications of all the available evidence**

Our global, high-resolution maps of *P falciparum* burden over time provide an evidence base to inform targeted malaria programme implementation and resource allocation. Such information is not only critical to further driving down malaria morbidity and mortality but also essential to identify subnational reservoirs for the disease and ultimately interrupt local transmission. Our maps highlight the continued, disproportionate burden of *P falciparum* borne by sub-Saharan Africa where, despite progress, many countries remain far from elimination. By providing a means of establishing international and intranational heterogeneity, our maps serve as an essential tool for assessing progress and guiding future intervention planning from local to global scales.

This research builds on previous efforts by the Malaria Atlas Project^1^, ^4^, ^5^ in which we used *P falciparum* parasite rate measured at fixed geographical locations to create pixel-level outputs. In Africa, the availability of *P falciparum* parasite rate household survey points supported the spatiotemporal results for parasite rate and incidence.^1^ Outside Africa, the relative scarcity of *P falciparum* parasite rate points motivated a change of our analytical framework to incorporate data for clinical malaria cases captured by routine health information systems. Such data, collated exhaustively from endemic countries across the Americas and Asia, provide geographically and temporally rich information on malaria burden at the clinic-level and administrative-level in otherwise poorly assessed regions of the malaria endemic world. However, these data necessitate careful interpretation and modelling to mitigate important sources of bias. Combining survey-derived *P falciparum* parasite rate and health system-derived case incidence data allow globally comparable burden estimates. The results presented here were combined with those for *Plasmodium vivax*^6^ and presented as all-species malaria estimates within the 2017 Global Burden of Disease (GBD) study.^7^, ^8^

## Methods

### Overview

To optimally incorporate varying types, quantity, and quality of available malaria data, we used separate modelling approaches for high-burden countries in sub-Saharan Africa and for other *P falciparum* endemic countries (appendix p 72). For 36 high-burden countries in sub-Saharan Africa, we used the cartographic approach in which we mapped *P falciparum* parasite rate at the pixel level and subsequently converted these results into estimates of clinical incidence and mortality. For the 70 remaining countries, we used the surveillance approach in which the response data consisted of reported *P falciparum* malaria cases. The surveillance approach was developed because *P falciparum* parasite rate survey points are rare in these countries, whereas routinely reported data are widely available and reliable. In the surveillance approach, we jointly modelled incidence and *P falciparum* parasite rate at the national level, modelled mortality from incidence, and then spatially disaggregated all three metrics to produce high-resolution maps. Both approaches used a rich set of temporally dynamic geospatial covariates for estimating spatiotemporal heterogeneity within *P falciparum* burden.

This analysis adheres to Guidelines for Accurate and Transparent Health Estimates Reporting standards. A table detailing our compliance to the guidelines is included in the appendix (p 76); statistical code is available through an online repository. Analyses were done with R version 3.4.4 or later.

**Table:** Comparison of *Plasmodium falciparum* all-age incidence and mortality from 2000, 2005, and 2017

	**Global**			**Sub-Saharan Africa**		
	2000	2005	2017	2000	2005	2017
Incidence (per 1000)	35·7 (30·5–42·1)	36·5 (31·2–43·6)	26·3 (21·2–32·6)	282·8 (234·8–339·6)	276·6 (232·6–335·5)	169·5 (134·6–212·4)
Incidence count (millions)	212·7 (181·5–250·8)	232·3 (198·8–277·7)	193·9 (156·0–240·2)	189·2 (157·1–227·2)	213·0 (179·1–258·3)	182·7 (145·0–228·9)
Mortality (per 100 000)	14·3 (8·7–21·4)	14·6 (9·4–21·1)	8·4 (5·0–12·9)	110·5 (70·2–162·2)	106·7 (71·0–152·3)	50·6 (36·9–76·6)
Mortality count (thousands)	850·3 (518·1–1271·6)	925·8 (596·9–1341·1)	618·7 (368·6–952·2)	739·3 (470·0–1085·1)	821·5 (546·4–1172·8)	545·2 (333·1–825·1)

Data are n (95% uncertainty intervals).

### The cartographic approach

The cartographic approach used for mapping *P falciparum* parasite rate, incidence, and mortality within 36 countries in sub-Saharan Africa was based on established methods,^1^, ^9^ which we applied to updated malaria measures^10^, ^11^, ^12^ and covariate^13^, ^14^, ^15^ datasets. This approach relied on household surveys collected for clusters of households at fixed geographical locations. The *P falciparum* parasite rate points came from national surveys collected by organisations such as the Demographic and Health Survey Program, and through systematic literature reviews of *P falciparum* prevalence studies.^11^ The resulting dataset consisted of 43 187 *P falciparum* parasite rate points collected from 2000 to 2017, and included data from 43 of 45 countries in sub-Saharan Africa (appendix p 4). The *P falciparum* parasite rate points were standardised to the 2–10 years age range (*P falciparum* parasite rate_2–10_)^16^ and to account for differing diagnostic methods.^17^ The predictor data consisted of geospatial environmental and socioeconomic data characterising vector and human habitats. The predictor dataset also included geospatial surfaces depicting temporally changing coverage of the main malaria control interventions such as insecticide treated bednets,^18^ indoor residual spraying, and effective treatment with an antimalarial drug.^19^ Each of the interventions was modelled independently and included to improve estimates and spatiotemporal predictions of *P falciparum* parasite rate.

These data were used in a Bayesian space-time geostatistical model to predict *P falciparum* parasite rate_2–10_ for every 5 × 5 km pixel across all of sub-Saharan Africa annually from 2000–17 (appendix p 8).^1^, ^20^ The results from this model were used for the 36 countries for which the cartographic approach was selected. The model allowed predictions to be made at any location by drawing strength from nearby *P falciparum* parasite rate observations, observations from earlier years, and by the relationships between those observations and the suite of covariates. We generated stochastic realisations from the model's posterior distribution, reflecting uncertainty due to availability and spatial heterogeneity within the *P falciparum* parasite rate points. The realisations also provided the basis for generating estimates of *P falciparum* parasite rate aggregated across national and subnational administrative units.

*P falciparum* incidence was calculated by converting *P falciparum* parasite rate_2–10_ into *P falciparum* incidence in three age-groups (0–4 years, 5–14 years, and 15 years and older) using an established relationship.^21^ This grouping reflected the biological relationship between the cross-sectional prevalence of infection in a given community and the resulting incidence of uncomplicated clinical malaria. This relationship was captured using an ensemble of mechanistic transmission models calibrated against observations at 30 sites from 24 studies that reported age-specific incidence of clinical malaria fevers and local *P falciparum* parasite rate.^22^ Statistical uncertainties from the calibration procedure were propagated through to predictions from this ensemble, which also allowed adjustments for the local effective treatment rate (proportion of individuals with a fever who seek treatment) and the seasonality of transmission.

*P falciparum* mortality was calculated using a combination of the pixel-level and case fatality rate (CFR) model for sub-Saharan Africa. The CFR modelling approach built on earlier work^9^ and used updated cause of death studies (verbal autopsy and vital registration reports) extracted from the GBD database.^12^ 70 such studies, which accounted for 1450 unique country, year, age-group, and sex combinations were used to parametrise the CFR model. From these studies, we took the proportion of deaths caused by malaria and multiplied the values by the associated all-cause mortality (another GBD derivative) to produce a site-level *P falciparum* mortality. We then divided *P falciparum* mortality by the site-level untreated incidence to get a site-level CFR value, with untreated incidence calculated as the product of the effective treatment and incidence. The site-level CFR values were used as the response variable in a geospatial modelling framework, which estimated pixel-level CFR for all of sub-Saharan Africa. Predictor variables in the CFR model consisted of gridded covariates for accessibility to cities,^15^ nighttime lights, and landcover types. The final step in the mortality modelling required adjusting the pixel-level mortality results via linear scaling so that the mortality totals within each administrative unit corresponded to GBD estimates that account for other causes of death.^23^

### The surveillance approach

For the remaining 70 *P falciparum*-endemic countries, located predominately outside of sub-Saharan Africa, we modelled *P falciparum* incidence using the surveillance approach, which relied on reported case data extracted from sources such as the World Malaria Report,^2^ online repositories typically compiled by national ministries of health, and peer-reviewed publications. Advantageously, the surveillance countries tended to have more robust health data reporting systems, but still required adjustments to the reported cases using methods defined by WHO.^24^ These adjustments accounted for factors such as treatment-seeking behaviour, under-reporting, and cases treated at private or non-formal sector health-care facilities and thus excluded from governmental statistics (appendix p 9). For elimination-phase countries, published *P falciparum* incidence counts were considered accurate as reported and not adjusted. The resulting database contained 69 120 aggregated case count estimates, of which 1721 were national-level and 67 399 were subnational. The subnational incidence estimates provided the critical information for modelling spatial heterogeneity in *P falciparum* within surveillance countries (appendix p 18).

Our modelling was based on national-level *P falciparum* surveillance reports, which were gap-filled for country-years in which no incidence data were available. We modelled the log-incidence time-series using a regression model with a random country-specific intercept, linear relations with gap-filled sociodemographic covariates, and short-term and long-term moving average terms to account for residual variation over the years (appendix p 18). Regional effects were incorporated by allowing countries in the same region to share the long-term trends. In *P falciparum* endemic countries where GBD produced subnational malaria estimates (Brazil, China, India, Indonesia, Iran, Mexico, and South Africa), the time-series modelling approach was applied at the level of the largest subnational units, with the restriction that the estimated subnational counts summed to the national estimates.

Although the time-series modelling produced administrative-level estimates, it did not produce pixel-level estimates. To create the high-resolution maps, we developed an approach for using subnational surveillance data to spatially disaggregate the time-series results within each country. In contrast to national-level data, subnational surveillance reports were often temporally sparse or only available for portions of countries (or both). Furthermore, even when complete, subnational incidence totals often did not match the national estimates. As such, these data provided a valuable yet challenging dataset for modelling spatiotemporal patterns in *P falciparum*.

The modelling technique underlying the spatial disaggregation approach was ecological downscaling.^25^, ^26^ We related pixel-level environmental covariates and a spatial Gaussian random-field to the administrative-level incidence data via a summation step within the link function. The regression parameters on the covariates, the mean of the random-field, and the hyperparameters of the random field were all learned by comparisons to the aggregated incidence data within a Bayesian modelling framework (appendix p 26). Where available, *P falciparum* parasite rate point data were used to inform the downscaling model by first creating pixel-level surfaces from these points using a suite of machine learning models, and predictions resulting from this initial analysis were included as new covariates. We then simultaneously modelled *P falciparum* parasite rate and *P falciparum* incidence using an established method to convert between the two metrics,^21^ while also ensuring that the incidence and *P falciparum* parasite rate values corresponded to the results from the national time-series modelling.

Mortality due to malaria in surveillance countries was estimated using the Cause of Death Ensemble model platform.^27^ This model examines a series of spatiotemporal Gaussian process regression and mixed-effects regression submodels with varying permutations of covariates, given some initial priors on covariate importance and direction of effect. The submodels are weighted via out-of-sample predictive validity and brought together into a final predictive model.^8^ We stratified our analysis by sex and age (older and younger than 5 years old), and used *P falciparum* incidence and the proportion of fevers effectively treated with an antimalarial used as the primary predictor variables. As with the cartographic approach, the final mortality estimates were adjusted using the all-cause mortality envelope to ensure consistency in relation to other diseases estimated within the GBD. As pixel-level data were not available outside of sub-Saharan Africa for differentiating treated and untreated cases, the final death totals were then linearly distributed within each country-year over the incidence count surfaces.

### Role of the funding source

The funder of the study had no role in study design, data collection, data analysis, data interpretation, or writing the report. All authors had full access to the data in the study and had final responsibility for the decision to submit for publication.

## Results

**Figure 1:**
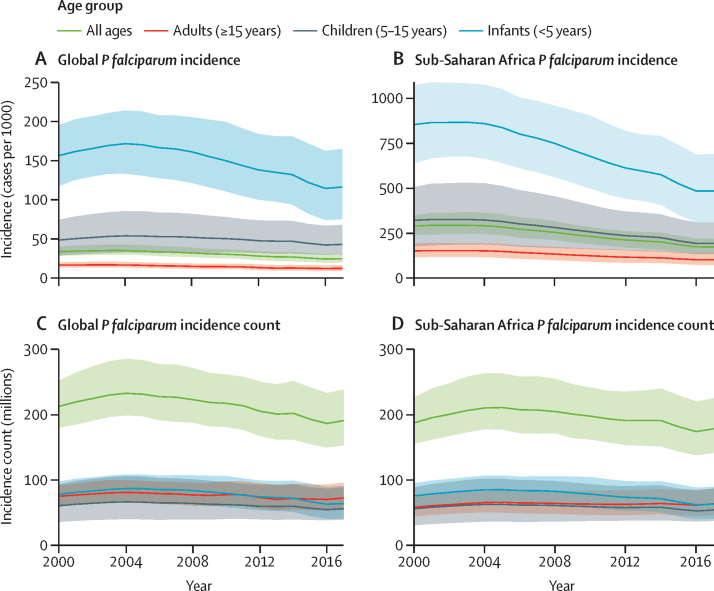
*Plasmodium falciparum* incidence (A and B) and count (C and D) globally and for sub-Saharan Africa from 2000–17 95% uncertainty intervals shown via the corresponding coloured bands around the mean lines. Rates were calculated using the total population in each age group in all endemic countries.

**Figure 2:**
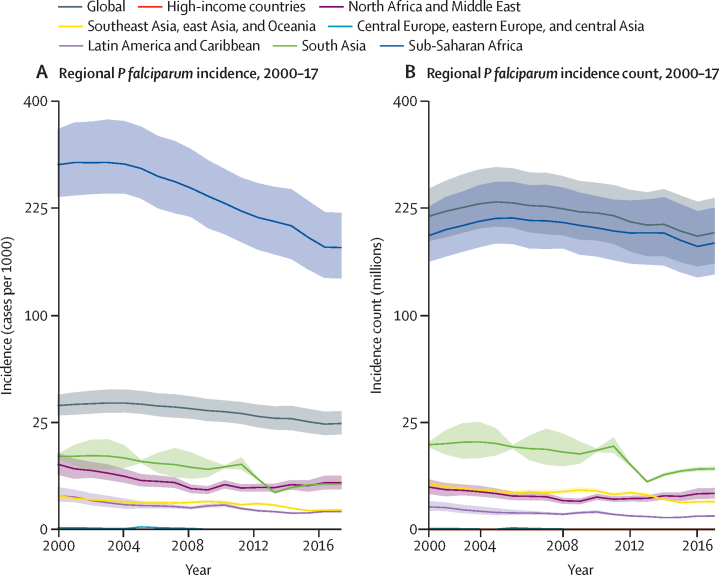
Regional distribution of *Plasmodium falciparum* incidence (A) and count (B) To show trends across regions with such different endemicity levels, the y-axis is scaled using the square root of incidence (per 1000 individuals) for A and count (in millions of cases) for B. 95% uncertainty intervals are shown via the corresponding coloured bands behind the mean lines. Rates were calculated using the total population in all endemic countries within each region.

**Figure 3:**
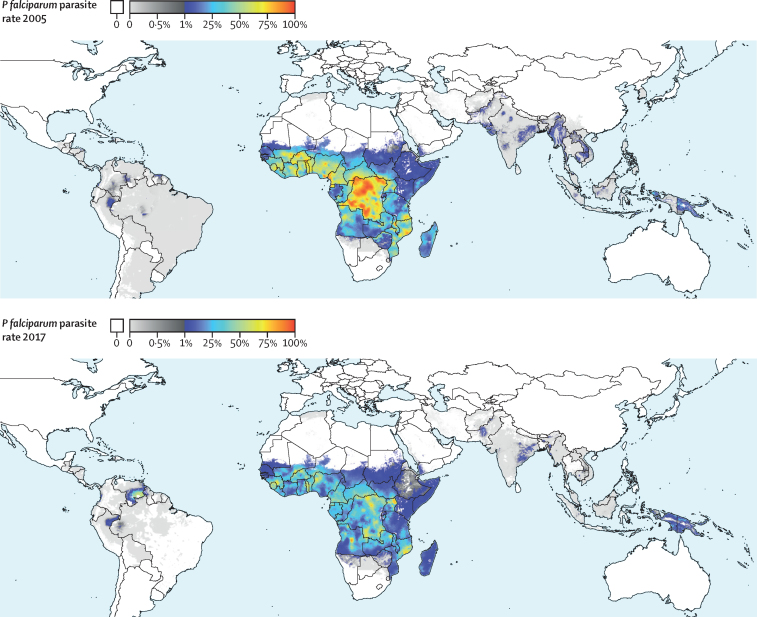
Spatial distribution of age-standardised *P falciparum* parasite rate_2–10_ in 2005 (top) and 2017 (bottom) Note the colour scaling is split to better differentiate within low endemic areas, with one linear scale between zero and 0·01 *P falciparum parasite rate*_2–10_ (grey shades) and a second linear scale between 0·01 and 1 (colours from blue to red). Areas without endemic *P falciparum* are shown in white. *P falciparum* parasite rate_2–10_=*P falciparum* parasite rate for children aged 2–10 years of age.

**Figure 4:**
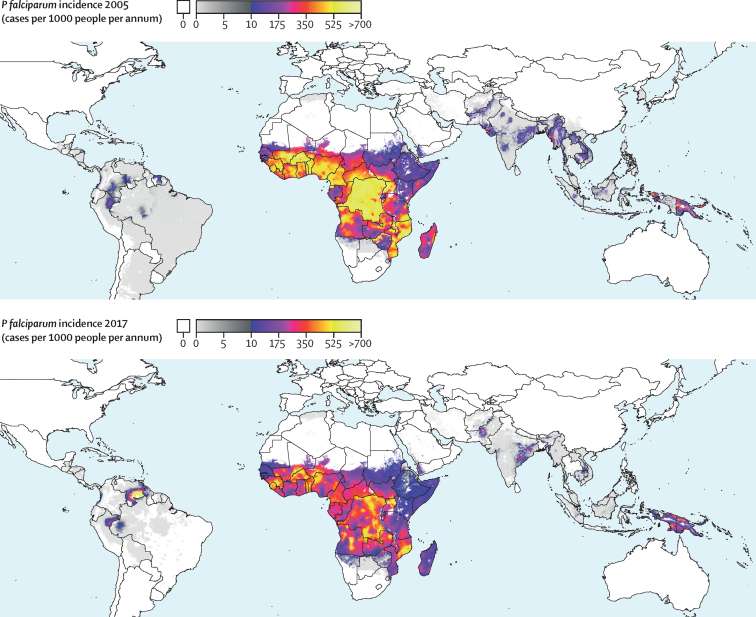
Spatial distribution of all-age *Plasmodium falciparum* incidence in 2005 (top) and 2017 (bottom) Note the colour scaling is split to better differentiate within low endemic areas, with one linear scale between rates of zero and 10 (grey shades) and a second linear scale between 10 and 1000 (colours from purple to yellow). Areas without endemic *P falciparum* are shown in white.

**Figure 5:**
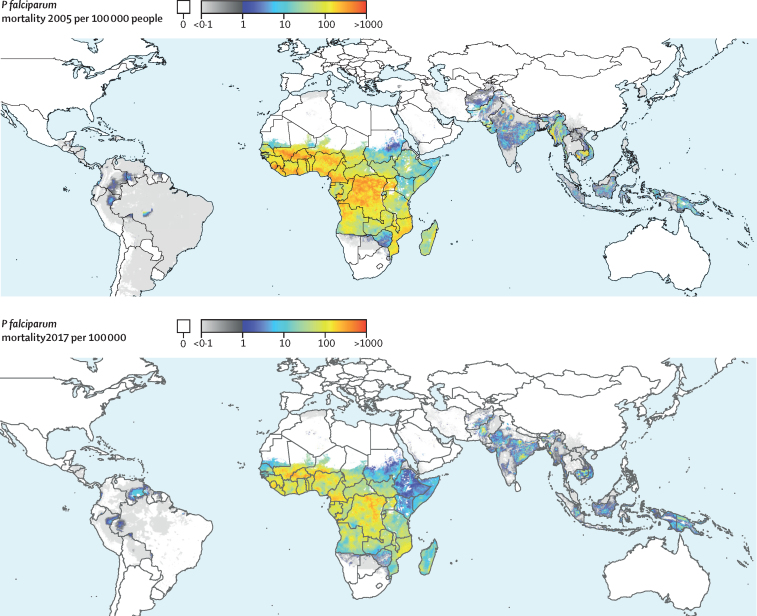
Spatial distribution of all-age *Plasmodium falciparum* mortality (deaths per 100 000 population per annum) in 2005 (top) and 2017 (bottom)

Global *P falciparum* incidence and mortality peaked from 2002–05 and have since declined across all age groups (figure 1, appendix p 73) and in all regions (figure 2, appendix p 74). The global maps for *P falciparum* parasite rate (figure 3), incidence (figure 4), and mortality (figure 5) in 2005 and 2017, illustrate both the progress that has been made in reducing *P falciparum* burden and the areas of continuing concern. The table highlights progress between 2005 (the peak year for number of cases globally) and 2017. Notably, within sub-Saharan Africa, both the incidence and mortality halved across this period. However, infants (aged 0–4 years) in sub-Saharan Africa bore a disproportionate amount of the global *P falciparum* burden across the study period, accounting for 42·6% (95% uncertainty interval [UI] 38·5–45·9) of global *P falciparum* cases in 2005 and 37·8% (27·9–44·5) of global *P falciparum* cases in 2017.

Despite high population growth in *P falciparum* endemic countries, the global percentage of individuals living in areas at risk of contracting *P falciparum* fell from 48·5% (95% UI 48·5–48·5) in 2005 to 40·9% (40·8–40·9) in 2017, highlighting the broad success of campaigns to control the disease (appendix pp 72, 75). The increase in the global percentage of humans living in *P falciparum*-free areas was driven largely by shifts from hypoendemic (ie, where *P falciparum* parasite rate is between 0–10%) to *P falciparum*-free areas, which were concentrated in south Asia and southeast Asia. In these regions, the percentage of the population living in *P falciparum*-free areas rose from 1·8% (95% UI 1·8–1·8) to 17·9% (17·9–17·9) between 2005 and 2017 in south Asia and 71·8% (71·8–71·8) to 82·2% (82·2–82·2) between 2005 and 2017 in southeast Asia. In terms of land area, 7·3% (95% UI 7·3–7·3) of the Earth's land surface (excluding Antarctica) transitioned from *P falciparum*-endemic to *P falciparum*-free during this time, which equates to 9·93 (95% UI 9·92–9·93) million km^2^, or an area roughly the size of China. The decrease in area in *P falciparum* endemicity is concentrated in South America, where Argentina and large portions of Brazil became *P falciparum*-free during the study period. In sub-Saharan Africa, the increase in *P falciparum*-free areas did not mirror the gains seen elsewhere as most of the population (90·9% [95% UI 90·9–90·9]) remained at risk of contracting *P falciparum* in 2017. However, the proportion of individuals in sub-Saharan Africa living in the highest burden areas has decreased substantially, as the percentage of the population living in hyperendemic or holoendemic regions (ie, where *P falciparum* parasite rate exceeds 50%) fell from 24·5% (95% UI 20·8–28·4) in 2005 to just 5·7% (4·3–7·5) in 2017. This decrease corresponds to nearly 122·4 (109·2–133·0) million fewer people living in the highest transmission areas in just 12 years. Declines in the highest burden areas of sub-Saharan Africa drive the increasing global percentage in the mesoendemic class (ie, *P falciparum* parasite rate from 10–50%), as *P falciparum* parasite rate in the higher burden areas in 2005 shifted into the more moderate category in 2017.

Downloadable means and uncertainty maps, national and subnational summaries, and data visualisation tools are available from the Malaria Atlas Project. National-level estimates were summarised from pixel-level results and provided key inputs for the total malaria (ie, combined *P falciparum* and *P vivax*) burden estimates in the 2017 GBD study.

## Discussion

The results presented here include the first high-resolution global maps of *P falciparum* prevalence and incidence produced since 2010. The findings are the first to be produced annually and show changes over time. The results also include the first high-resolution global maps of *P falciparum* mortality. These results provide a means of assessing progress in the fight against malaria and an evidence base for malaria control programme managers tasked with directing resources to areas of greatest need. Likewise, our results provide a resource for identifying areas with inadequate intervention coverage, or where the supplied interventions are underperforming (or both). Unlike estimates derived at the national level, our maps enable fine-grained evaluations of the relationships between interventions and burden by increasing the number of observations from 106 endemic countries to 1·6 million pixels that span a wide range of epidemiological, economic, and political settings.

Our results illustrate that although *P falciparum* has declined globally both in terms of spatial extent and burden intensity, the disease remains pervasive in sub-Saharan Africa despite a halving in incidence and mortality since 2005. Declines in malaria were likely to be driven by multiple factors, including widely increased access to insecticide treated bednets^18^ and artemisinin combination therapy antimalarial drugs,^19^ improved health-care systems, increased urbanisation, and reduced poverty. In areas where *P falciparum* remains a major cause of morbidity and mortality, its persistence is made more complex by political instability, vector habitat changes, the rise of insecticide and antimalarial drug resistances, importation of cases due to human movement, and shifting funding priorities as *P falciparum* burden declines and other public health needs take precedence. These phenomena pose a grave threat to the progress made against *P falciparum,* and a resurgence in the disease is possible should the political will or funding for malaria elimination campaigns fade. To sustain the gains made against *P falciparum* and further reduce its burden, the support for key malaria control interventions should be maintained, as should efforts to improve the effectiveness of interventions to maximise their effect within a constrained budget.

The Malaria Atlas Project contributes results to both the World Malaria Report 2017^2^ and the GBD studies,^28^ thus making comparisons with alternate global burden estimates challenging. However, by comparing our results with these sources, we can show key differences between available estimates. Unlike the results presented here, those published in the alternate sources consisted of pooled estimates for all *Plasmodium* species that were summarised by administrative level. To create a comparable dataset, we merged our *P falciparum* results with those we produced for *P vivax*^6^ and then derived population-weighted summaries globally and for each malaria endemic country. Our results were very similar to those from the alternative sources for years 2000–10, which was expected. Since 2014, our results differ slightly from the other sources, which illustrates the impact of newly incorporated malaria burden and intervention data. For 2016, we estimated 10·1 million fewer malaria cases (4·8%) than the estimates for that year in GBD 2016, and 12·9 million fewer cases (6·0%) than the corresponding year estimate from the World Malaria Report 2017. The discrepancy between our data and that of the World Malaria Report 2017, also highlights the effect of an alternate incidence estimation method applied outside of Africa by WHO when deriving results for the World Malaria Report. With respect to global mortality, we estimated 93 800 fewer deaths (13·1%) than GBD 2016, and this decline is due to reduced incidence and adjustments made to the GBD 2017 cause of death dataset. The estimates for number of deaths presented here show 182 000 more deaths (40·7%) than those presented in the World Malaria Report 2017, which is attributable to fundamentally different approaches used for calculating malaria mortality.

Decreasing *P falciparum* morbidity and mortality has coincided with the increased availability of datasets that provide estimares of burden. The results presented here represent a substantial improvement over previous global maps of *P falciparum* burden^4^ due to the incorporation of routine surveillance data, which vastly increased the information available for modelling the disease outside of Africa. An outstanding question, however, pertains to the use of surveillance data in sub-Saharan African countries for which we applied the cartographic method. Surveillance data provide another source of evidence for assessing *P falciparum* burden in these countries and such data could inform future versions of our maps. Surveillance data also lack potential biases within the *P falciparum* parasite rate response dataset, including points gleaned from literature reviews that cluster in high-burden areas and issues stemming from the timing of PR survey collection relative to seasonally variable malaria transmission. Despite these advantages, a substantial challenge when incorporating surveillance data from high-burden countries is that most of the reported *P falciparum* cases represent a patient testing positive for malaria infection at the point of care via a rapid diagnostic test or microscopy. This definition does not distinguish patients with true cases of clinical malaria from patients harbouring asymptomatic malaria infections who are seeking care for another reason. Critically, our definition of incidence distinguishes causal and non-causal infections, based on parasite density thresholds,^21^, ^29^, ^30^ and this distinction leads to potentially large disparities with incidence data observed in active case detection. The ideal situation for producing *P falciparum* burden estimates in high-burden countries would be to hybridise the cartographic and surveillance methods, but developing such models will require both high-quality surveillance data and point measurements of *P falciparum* parasite rate to be present in the same location and time period. This confluence of data types is rare. Nevertheless, for future maps we will explore hybrid approaches and methods for quantifying the factors that lead to disparities between competing methods.

Although high-burden areas in sub-Saharan Africa receive the most research funding and focus, far more people live within hypoendemic regions that have low but persistent *P falciparum* transmission. Our results will be particularly relevant for policy discussions in endemic countries outside Africa as they are the first high-resolution burden estimates produced for such areas since 2010.^4^ Substantial progress was made in malaria reduction between 2000 and 2017 in these regions, but due to high population densities in areas such as south Asia and southeast Asia, over 2·6 billion individuals remain at risk, albeit a low risk, of contracting *P falciparum*. This risk will remain until *P falciparum* is fully eliminated, but achieving elimination is made more complex by the persistence of submicroscopic infections,^31^ which remain harmful^32^ and contribute to onward transmission.

The results presented here illustrate the great progress that has been made in combating malaria in the past two decades as a result of global efforts to reduce morbidity and mortality of this disease. By providing detailed maps quantifying high-resolution spatiotemporal patterns of *P falciparum* burden, our results will serve as a valuable resource for assessing the localised efficacy of intervention efforts across a range of settings as well as highlighting areas where burden remains high or progress has stalled. These results contribute directly to global malaria burden estimates in the World Malaria Report and the GBD study from 2015 onwards, and thus form the basis of the official UN numbers relied on by agencies including the Global Fund and national ministries of health. Furthermore, international policy and guidance^33^, ^34^ increasingly advocate for planning and implementation of malaria control at a subnational level using approaches tailored to local conditions, a prerequisite for which is granular evaluation of risk and burden such as the high-resolution results presented here. Remaining and emerging challenges in the fight to eliminate *P falciparum* globally highlight the need for health-care providers, malaria researchers, those developing and improving interventions, and the international funding community to be resolute in their efforts to eliminate the disease. Our results provide a valuable resource to these communities and will aid their decision making processes for years to come.

## Data sharing

All pixel-level and administrative-level summaries are available for visualisation and download at www.map.ox.ac.uk/malaria-burden.
