# Supplementary material for: Mapping the global prevalence, incidence, and mortality of Plasmodium falciparum, 2000–17: a spatial and temporal modelling study
Source: Lancet. 2019 Jul 27;394(10195):322–31. doi: 10.1016/S0140-6736(19)31097-9 (PMC6675740; doi:10.1016/S0140-6736(19)31097-9)
Supplement: Supplementary appendix [file mmc1.pdf]

THE LANCET

Supplementary appendix

This appendix formed part of the original submission and has been peer reviewed. We post it as supplied by the authors.

Supplement to: Weiss DJ, Lucas TCD, Nguyen M, et al. Mapping the global prevalence, incidence, and mortality of *Plasmodium falciparum*, 2000–17: a spatial and temporal modelling study. *Lancet* 2019; published online June 19. http://dx.doi.org/10.1016/S0140-6736(19)31097-9.

Supplementary Material for "The global landscape of *Plasmodium falciparum* prevalence, incidence, and mortality 2000–2017"

Weiss, D.J., Lucas, T.C.D, Nguyen, M., Nandi, A., Bisanzio, D., Battle, K.E., Cameron, E., Twohig, K., Pfeffer, D., Rozier, J., Gibson, H., Rao, P., Casey, D., Bertozzi-Villa, A. Collins, E., Dalrymple, U., Gray, N.,Harris, J., Howes, R. Kang, S., Keddie, S., May, D., Rumisha, S. Thorn, M., Barber, R., Fullman, N., Huynh, C., Kulikoff, R., Kutz, M., Naghavi, M., Nguyen, G., Shackelford, K., Vos, T., Wang, H., Smith, D.L. Lim, S., Murray, C.J.L., Bhatt, S., Hay, S.I. and Gething, P.W.

Contents

1 Morbidity **2**
1.1 Data 2
1.1.1 Raster covariates 2
1.1.2 Population data 2
1.1.3 *Pf* PR data collection 3
1.1.4 Treatment-seeking data assembly 3
1.1.5 Surveillance data collection 5
1.2 Methods 8
1.2.1 Prevalence to incidence conversion 8
1.2.2 Africa prevalence model 8
1.2.3 Treatment-seeking model 9
1.2.4 API estimation 9
1.2.5 Outside of Africa: time-series models 18
1.2.6 Outside of Africa: disaggregation regression 25
1.3 Results 58

2 Mortality **64**
2.1 Data 64
2.2 Methods 64
2.2.1 Mortality in Africa 64
2.2.2 Mortality in surveillance countries 64
2.3 Results 65

3 Schematic diagrams **69**

4 Extended figures and table **72**

5 GATHER compliance **76**
5.1 Checklist 76

References **78**

1 Morbidity

1.1 Data

1.1.1 Raster covariates

A number of rasterised environmental and anthropological covariates at 2.5 arcminute (approximately 5km × 5km) resolution were used. The covariates and their associated references and processing notes are given in the table below.

Table S1: Covariates used.

Covariate	Source	Processing
IGBP Combined Forest	MODIS MCD12Q1[1]	Process 1
EVI Mean	MODIS MCD43B4[2]	Process 2
EVI SD	MODIS MCD43B4[2]	Process 2
LST Daytime Annual Mean	MODIS MOD11A2[3]	Process 2
LST Daytime Annual SD	MODIS MOD11A2[3]	Process 2
LST Night Annual Mean	MODIS MOD11A2[3]	Process 2
LST Night Annual SD	MODIS MOD11A2[3]	Process 2
TCB Annual Mean	MODIS MCD43B4[2]	Process 2
TCB Annual SD	MODIS MCD43B4[2]	Process 2
Precipitation	WorldClim[4]	Overall local mean
Accessibility	Weiss et al.[5]	2.5 arcminute spatial mean
Nighttime lights (2010 stable lights)	DMSP F18 Satellite	2.5 arcminute spatial mean
Elevation	SRTM 3 arcsecond Digital Elevation Model[6]	2.5 arcminute spatial mean
CGIAR-CSI Global PET Database	Zomer et al.[7]	2.5 arcminute spatial mean

The processes referred to in the table above are:

- Process 1: The MODIS MCD12Q1[1] data were downloaded for the closest available relevant year (2013: the data are not available for subsequent years) and the IGBP landcover band was extracted, reprojected, and merged to a global lat/lon GeoTIFF grid at 15 arcsecond (approximately 500m) resolution. The various IGBP classes representing types of forest (Evergreen Needleleaf forest, Evergreen Broadleaf forest, etc) were selected and reclassified to a single forest/not-forest grid. This grid was then aggregated by a factor of 10 to 2.5 arcminute (approximately 5km) resolution, where the output cell value represents the percentage of the 100 input cells that were classified as any forest.
- Process 2: The MODIS MCD43B4 (BRDF reflectance)[2] and MOD11A2 (land surface temperature)3 products are available at an 8-daily interval. These were downloaded for the entire period of data availability and were converted to GeoTIFFs for the relevant metric by extracting the relevant bands and performing the necessary calculations to convert to the required indices such as EVI, before reprojecting and merging to global lat/lon GeoTIFFs at 30 arcsecond resolution. All of these grids were then gapfilled using the algorithm published by Weiss et al[8]. These 8-daily 30 arcsecond grids were then aggregated to 8-daily 2.5 arcminute (taking the spatial mean value of the 25 source pixels) and then those were aggregated (temporally) to annual summaries (using the spatial mean and SD values derived from the 25 source pixels).

1.1.2 Population data

Population figures were provided by the Institute of Health Metrics and Evaluation (IHME), the University of Washington, Seattle. The IHME population figures are derived from the United Nations (UN) official estimates

of population. Figures were provided at national level for all countries and at administration level one for Kenya, Saudi Arabia, Brazil, India, China, Mexico, and Indonesia.

Initial global raster surfaces of population were created using a hybrid of data from GPWv4[9] and WorldPop[10], with the latter taking priority for those pixels where both had population data. A raster was created in this manner for each year in which GPWv4 and WorldPop data were available (2000, 2005, 2010, 2015). For the intervening years (i.e. 2001-2004, etc), initial population rasters were created by linear interpolation of the surrounding five-yearly rasters.

For each model year, a raster of IHME population was then created by distributing the IHME population figures for country/administrative units across the pixels bounded by each country/administrative unit, in the same proportions as the corresponding pixels in the hybrid GPWv4 / WorldPop raster for the corresponding year. That is, for a given IHME administrative unit for which a population figure was available, we calculated the total of the pixels in the initial raster for that year, divided this by the corresponding IHME figure for that administrative unit / year, and then divided the value of all the pixels in that administrative unit by the resulting number to ensure that the total of the pixels matched the IHME figure. As IHME values were available for years prior to 2000 but initial population grids were not, for years prior to 2000 we used the 2000 grid as the initial value. This process produced rasters matching total administrative population values from IHME with the pixel-level population values determined by the proportions of the initial rasters.

MAP has previously published a global limits layer outside which transmission of malaria is highly unlikely.[11] This layer was based on environmental factors, travel guidelines, and statements by the countries regarding their malaria-endemic status in 2010.

An amended version of this global limits layer was created excluding the malaria-endemic status of the country. This exclusion was necessary because the research project covered data extending back to 2000 during which time the status of many countries have changed.

This new global limits layer was applied over the IHME-adjusted population rasters to set population values in pixels outside the limits of transmission to be zero, resulting in population-at-risk grids.

Rasterized versions of GADM[12] geometry files for both sub-national administrative units and national borders were then used to provide a set of pixels in the IHME raster to sum to produce the population-at-risk for those sub-national units. Eritrea was an exception in that GAUL[13] geometry files were used rather than GADM.

1.1.3 *Pf* PR data collection

Plasodium falciparum parasite rate (*Pf* PR) data came from geopositioned community-based survey measurements of PfPR identified available from surveys like those conducted by the Demographic and Health Survey (DHS) program and through periodic literature searches from published data sources and direct communication with malaria specialists for unpublished measurements of *Pf* PR. The resulting *Pf*PR dataset (n = 43,187) included data from 43 of 45 *Pf* endemic countries in Sub-Saharan Africa (Figure S1). Further details of the collation of this data can be found in previous publications.[14,15]

1.1.4 Treatment-seeking data assembly

Data on treatment-seeking behaviours in malaria endemic countries were gathered from DHS and Malaria Indicator Surveys (MIS) that were conducted from the year 1995 onwards. Treatment-seeking rates were determined from the number of children reported to have fever in the past two weeks for which treatment was sought. Response codes were manually classified into treatment at public points of care, such as government hospitals, clinics and community health workers; and any treatment, which included all public treatment as well as private and non-governmental organization (NGO) facilities. Friends, family and traditional and homeopathic healers were not considered as treatment. Data downloading, extraction and processing was automated using Feature Manipulation Engine (FME), version 2017, by Safe Software, using data obtained from the DHS online platform (https://dhsprogram.com/).[16] Sampling weights were extracted and applied at the individual level following DHS guidelines[17] and the total number of children, fever cases and cases that sought treatment (public- and any-) were summarized nationally. Where data were extreme outliers, the value from the survey report was used instead as the extreme difference pointed to some error in the automation (e.g. Bolivia, 2008).

Data were extracted from a total of 152 surveys from 56 countries. These spanned the six WHO regions with 95 surveys in the African region (AFRO), two in the Eastern Mediterranean (EMRO), three in Europe (EURO), 27 in

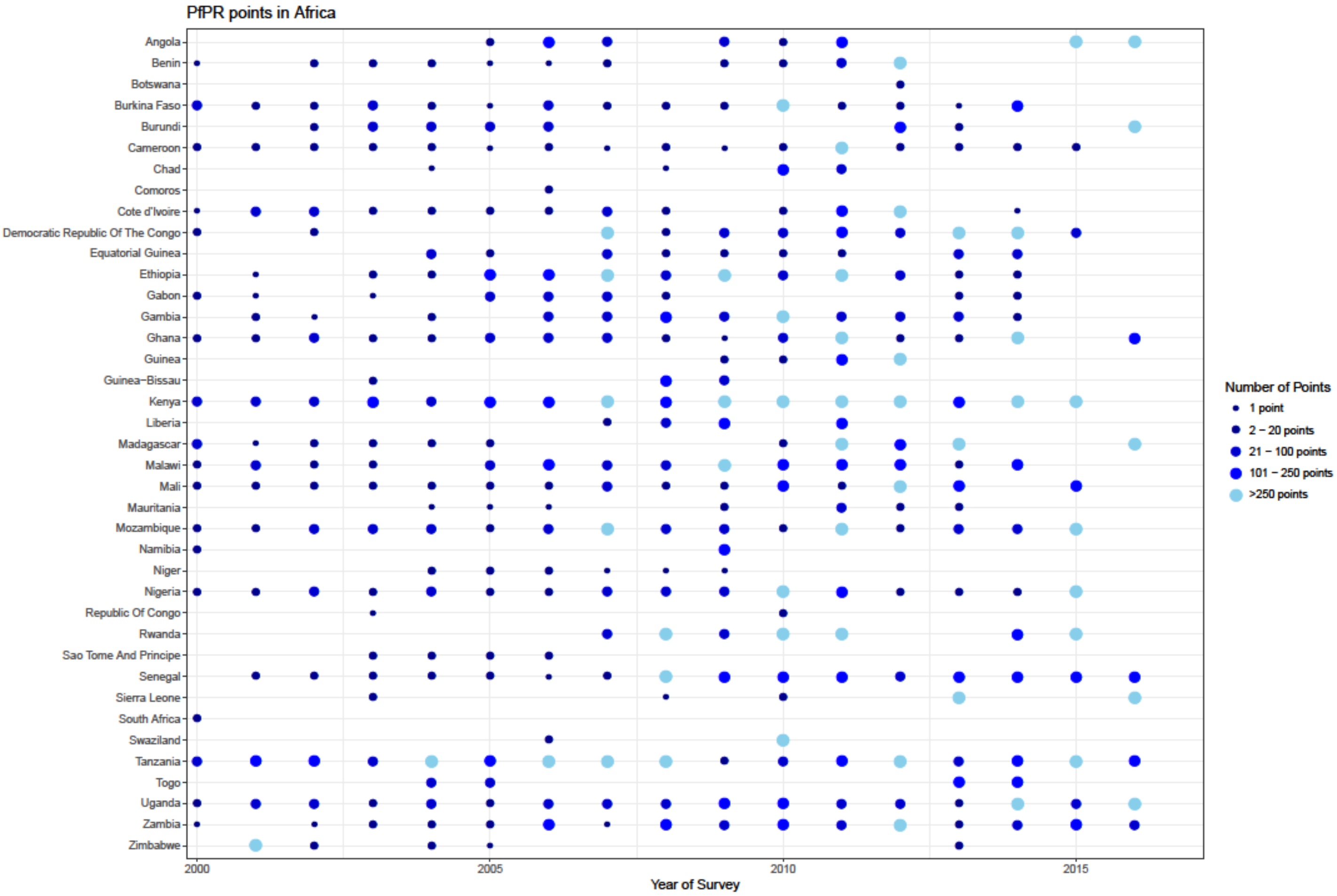

Figure S1: *Pf* PR AFRO data summary.

the Americas (PAHO), 17 in the South-East Asian region (SEARO) and 8 in the Western Pacific (WPRO). For descriptive purposes, countries in the African region were separated into the sub-African regions reported in the World Malaria Report: West Africa (AFRO-W), Central Africa (AFRO-C), East Africa and high-transmission areas in Southern Africa (AFRO-E), and low-transmission Southern African countries (AFRO-S).[18]

Treatment-seeking rates were reported as national mean values for the percentage of children under the age of five with fever occurring in the two weeks before the survey, that were taken for treatment at a public or any type of facility. Treatment-seeking rates for India were reported as subnational (ADMIN1) mean values. These were calculated from the weighted individual child records from each survey. Upper and lower confidence intervals (95% CIs) for the national rates were calculated using the "survey" package in R.[19]

Socio-economic indicator data were obtained from the World Bank.[20] The variables selected had been identified as strong indicators of treatment-seeking rates from a previous literature review and statistical analysis.[21] These included indicators of wealth and health infrastructure: gross domestic product (GDP) growth, health expenditure (% of GDP) and out-of-pocket health expenditure (% of total expenditure on health). Accessibility of care shown by the proportions of pregnant women that sought prenatal care and children immunized for diphtheria pertussis and tetanus (DPT). Finally, overall level of education was represented from rates of primary education completion. Matching indicator data to treatment-seeking rates was also done using the FME tool.

1.1.5 Surveillance data collection

The suitability, availability and quality of *Pf* PR and routine case reporting data, as well as detailed intervention coverage information, differs markedly inside versus outside Africa that separate modelling strategies were developed for countries inside Africa versus those outside. The exceptions were Algeria, Egypt, Morocco, Comoros, Mauritius, Cape Verde, Sao Tome and Principe, Botswana, Namibia, Eritrea, Djibouti, and South Africa. The modelling strategy use for most of Africa does not work well for island nations. The other countries have data availability that made modelling them with non-African countries more appropriate.

Malaria endemic countries outside Africa tend to have less *Pf* PR data than those inside, in part because prevalence is generally lower and thus *Pf* PR becomes an inefficient way to measure malaria risk. Conversely, routine surveillance systems outside Africa are generally stronger, meaning that reports of malaria cases from health systems are more reliable and provide some insight into the total malaria burden in the community. The protocol for collecting this surveillance data is detailed below.

1.1.5.1 Data selection criteria

Rules for data selection were developed to address conflicts arising between data sources for any given administrative unit in a country for a given year. Where there was consistency in the data reported between sources, it would be possible to apply a simple rule e.g. favouring, the most recently published source. However, it is justifiable to assign a greater preference to some sorts of data over others regardless of the publication date. For example, two different sources might report the following conflicting data for a given administrative unit and year:

- Microscopy figures for explicitly stated indigenous species-specific cases
- A figure for API, with no indication on how this figure was calculated and whether or not it included only indigenous cases

In this example, the microscopy-based figures offer estimates that are more robust for that administrative unit for that year, even if the overall API figures were published more recently.

In order to determine which figures should feed into the model in cases of conflict, data was assessed using up to three steps of processing with the following rules:

1. Allocate all data for a given admin unit-year combination to a "band" according to its perceived usefulness and reliability.
    - If the highest-ranking band for which there are data contains data from only one source, that data is used.
    - If the highest-ranking band for which there are data contains conflicting data from multiple sources, these data are processed as per step 2 – all data from lower-ranking bands are discarded.

2. Each set of figures from the same band is allocated points, according to the points table applicable to that band. This avoids giving points for irrelevant data – the points are for figures that are relevant for computing API with the data from that band.
    - If a set of figures from one source has higher points than all other sources, then that set of figures is preferred.
    - If two or more sources have equally high points, then those are processed as per step 3 and the other lower-scoring sources for that band are discarded.
3. For sets of figures from equally-scoring sources, the most recently published source is preferred. If the sources have the same publication date, one is chosen at random.

We allocate all data for a given admin unit-year combination to a band according to its perceived usefulness and reliability. Table S2 details the variety of data reported ranked in descending order of perceived usefulness and reliability.

A row in a higher band will always be preferred over a row in a lower band.

Table S2: Data bands.

Band	Band name	Band description
1	Has indigenous microscopy or RDT confirmed cases by species	Indigenous microscopy or RDT confirmed cases by species is always the most desirable data.
2	Has non-explicitly indigenous microscopy or RDT by species but no imported cases	The fact these microscopy and RDT results are not explicitly stated to be indigenous is not an issue where there are no imported cases, so these are second only to explicitly indigenous results.
3	Has non-explicitly indigenous microscopy or RDT by species and species specific imported cases	Microscopy or RDTs with species breakdowns but not explicitly stated to be indigenous are slightly less ideal if there are imported cases, even if these are by species, since it forces the assumption that these need to be taken off, when this may actually vary by source reporting practice.
4	Has non-explicitly indigenous microscopy or RDT by species and has non-species-specific imported cases	Microscopy or RDT results with species breakdowns but not explicitly stated to be indigenous are less ideal if there are imported cases, especially where these are not by species, since a) it forces the assumption the imported cases should be subtracted from the total cases identified by the test results â€“ i.e. we have to assume the test results include both indigenous and imported cases because it is not otherwise stated in the source b) these have to be taken off by species according to the ratio.
5	Has indigenous microscopy with no species breakdown	Microscopy or RDT results are better than confirmed cases without justification as to how they were confirmed. The best of these are explicitly indigenous, since there are no worries about
6	Has non-explicitly indigenous microscopy or RDT with no species breakdown but no imported cases	imported cases. Microscopy or RDT results which are not broken down by species or explicitly stated to be indigenous where there are no imported cases to take off anyway are the next best results.
7	Has non-explicitly indigenous microscopy or RDT with no species breakdown and imported cases	Microscopy or RDT results which are not broken down by species and are not explicitly stated to be indigenous where there are imported cases to remove, whether or not these imported cases are species specific.
8	Has confirmed cases without diagnostic details by species and no imported cases	Confirmed cases by species are preferred less than data with microscopy or RDT, because their confirmation method is unspecified. Those without imported cases to take off are preferred.

Band	Band name	Band description
9	Has unjustified confirmed cases by species and imported cases by species	Where there are imported cases to take off confirmed cases by species, it is better to know the species breakdown of the imported cases than to have to compute an estimate.
10	Has unjustified confirmed cases by species and imported cases not broken down by species	Here there are confirmed cases by species, but the imported cases must be taken off based on the proportion of Pf:Pv:Other as given in the report if provided, or by a national estimate otherwise.
11	Has unjustified confirmed cases not broken down by species and no imported cases	Confirmed cases without justification or species breakdown must be reallocated by species; there are no imported cases to take off, though, which is preferred.
12	Has unjustified confirmed cases not broken down by species and imported cases	Confirmed cases without justification or species breakdown must be reallocated by species, and the imported cases taken off (and reallocated by species where these are not reported by species).
13	Has total cases explicitly stated to be local population	Where the only cases are unconfirmed, those explicitly stated to come from the local population take precedence.
14	Has explicit unconfirmed cases not stated to be local, or total cases not stated to be local	Where the only cases are unconfirmed, and this is not explicitly stated to come from the local population.
15	Has reported API	The lowest band is where only API is reported, and must be transformed back into cases via the administrative unit population taken from IHME (and hence UN) figures.

When there are competing sources within a single band, these are tie broken according to points system below.

Table S3: Tie-break points.

Bands	Condition	Points
1,5	Has an accompanying figure for number of tests undertaken	1
2–4, 6–7	Has microscopy confirmation	1000
	Has RDT confirmation	100
	Has microscopy results and an accompanying figure for number of microscopy tests undertaken, plus unconfirmed or total cases (local pop or not) values	10
	Has RDT results and an accompanying figure for number of RDT tests undertaken, plus unconfirmed or total cases (local pop or not) values	1
8–10	Has a figure for *P. falciparum*	10
	Has a figure for *P. vivax*	1
11–12	Has a reported percentage or proportion of *P. falciparum* or *P. vivax*	1
13–15	Has a reported slide positivity rate (SPR) or test positivity rate (TPR) to allow estimation of percentage confirmed cases	10
	Has a reported percentage or proportion of *P. falciparum* or *P. vivax*	1

In the event of all the above criteria being equal for two or more competing sets of values, one was chosen at random.

1.2 Methods

1.2.1 Prevalence to incidence conversion

In a number of contexts throughout the analysis we wish to convert data from prevalence (*P. falciparum* parasite rate in ages 2–10, in the interval [0, 1]) to incidence (per person per year, in the interval [0, ∞]. To do this we use a model that was published previously.[22] This involves applying an emulator approach to three *P. falciparum* microsimulation models and a standardized calibration data set to obtain an ensemble model for the *Pf* PR-incidence relationship. While the approach itself addresses model uncertainty, the random effect terms in the model account for uncertainty due to the limited and noisy data by allowing for local, data-driven variations in the relationship between prevalence and incidence. After fitting, this model defines a function

$$\text{Prev2Inc}: f(P) = 2.616P - 3.596P^2 + 1.594P^3.$$

We call this function the prevalence-incidence relationship. By applying this function, we do not propagate the uncertainty in the relationship itself; however, by doing so at the realisation level, we propagate uncertainty from the *Pf*PR model. The rationale for this decision is to make the conversion computationally tractable over so many pixels and realisations.

We also use the inverse of this model to calculate national-level *Pf* PR from national incidence estimates from time-series modelling (see section "Outside of Africa: time-series models"). However, due to the non-linear nature of the relationship, converting national-level incidence to prevalence does not give the same answer as converting pixel incidence to prevalence and taking the population-weighted mean. The value calculated from national-level incidence are therefore only used as covariates in CODEm models and not as final results. Instead, final aggregate prevalence values are calculated as the population weighted mean of pixel prevalence.

While the prevalence-incidence relationship is a function over all values of prevalence ([0, 1]), the simple inverse of the equation is neither a function nor defined over all possible values of incidence. Therefore, for the reverse relationship, we cap incidence at the maximum value given by the prevalence-incidence relationship,

$$P_{\max} = \arg\max_{P} \text{Prev2Inc}(P).$$

This corresponds to a maximum prevalence $P_{\max} = 0.616$ and a maximum incidence rate of $I_{\max} = 0.620$ per person per year.

This inverse relationship also has no simple analytical form, so is solved numerically in each instance.

The incidence-prevalence function is therefore

$$\text{Inc2Prev}: f(I) = \begin{cases} \text{Prev2Inc}^{-1}, & \text{if } 0 < I < I_{\max} \\ P_{\max}, & \text{if } I > I_{\max} \end{cases}$$

As with the prevalence-incidence function, we apply the incidence-prevalence function at the realisation level to propagate uncertainty from the time-series models.

1.2.2 Africa prevalence model

The large assembly of geolocated *Pf* PR surveys maintained by MAP was used in a Bayesian spatiotemporal geostatistical model to predict *Pf* PR for every pixel-year in sub-Saharan Africa, representing an update to earlier work.[14,23] The model took into account (i) *Pf* PR survey participant age ranges and diagnostic type; (ii) coverage of ITNs, IRS and treatment with an effective antimalarial drug and how these metrics changed through time at each data and prediction location; (iii) environmental conditions at each data and prediction location (including density of vegetation, temperature, humidity, rainfall, elevation, proximity to populated areas). The outcome was a predicted space-time "1cube" of *Pf* PR, standardized to the 2–10 age range, for each year 2000–2016.

The *Pf* PR cube was then converted into an equivalent cube of the predicted incidence rate of clinical malaria using the prevalence-incidence relationship. This cube was then stratified into three broad age bins (0–5; 5–14; >15) using age-splitting models fitted previously.[22]

1.2.3 Treatment-seeking model

1.2.3.1 Gaussian process to gap-fill the World Bank indices

In order to predict treatment-seeking rates for the period 2000–2016, it was necessary to build a gap-filled time-series of the World Bank indices. The approach used to create this was based on Gaussian process regression (GPR).[24] To keep each index independent, the GPR was performed using only time (year) as a covariate. This reduced the possibility of introducing circularity in the time-series which could have been created by using other World Bank indices as covariates for the regression process. A GPR, based on a Gaussian process (GP),[25] is well-suited for estimating missing data and making forecasts in of time-series. A detailed mathematical description of GP procedures and its implementation can be found in.[24] The GP implemented to estimate missing data in each World Bank index had the following form:

$$y = f(\text{Year}) + N(0, \sigma^2)$$

Where y is the missing point in the World Bank index, $f(\text{Year})$ is the non-linear effect of year and $N(0, \sigma^2)$ is Gaussian noise. The GPR was performed using R26 package GPfit27 which performs GPR using algorithm described in.[28] For countries with greater than 90% missing data for the World Bank indices time-series, information was borrowed from other countries belonging to the same WHO region. These were made by performing a GPR on the indices using the limited data available from said country and randomly selected points from countries of the same region. All GPR processes were performed 1000 times to calculate the uncertainty of the estimates.

1.2.3.2 Model approach

Generalized additive mixed models (GAMM)[29] were applied to obtain estimates of treatment-seeking rates for malaria endemic countries for the years 2000 to 2016. To calculate the proportion of children under five with fever that sought treatment at any type of provider or at public/government points of care, two GAMMs were built using information from indicator variables described above following covariate selection as described previously.[21] The GAMM for seeking treatment at any type of facility included the proportions of the population that completed primary education and pregnant women that received prenatal care as covariates. Treatment-seeking at public facilities were predicted using public health expenditure and proportion of pregnant women given prenatal care. To account for temporal and spatial autocorrelation, both models included the survey year as a non-linear effect and the WHO region. To calculate uncertainty of predicted treatment-seeking estimates, the models were run 1000 times. Each run sampled from the range of the 95% CIs of the observed treatment-seeking rates and indicator variable values as described above.

1.2.4 API estimation

1.2.4.1 AFI formulae

Annual *Plasmodium falciparum* incidence per 1000 population per year (AFI), was calculated at the national level for all countries and at every available sub-national level for which record sets could be obtained. The formula used for calculating non-species-specific annual parasite incidence per 1000 population per year (API) for a given administrative unit and year is trivial:

$$\text{API} = 1000\frac{M}{\text{population}}$$

where M is the number of cases for that administrative unit and year. However, the data gathered only includes cases that have been captured by the healthcare reporting systems of the respective countries. For countries with poorly developed health management information systems (HMIS), this might represent an under-reporting of cases. To obtain an estimate closer to the true number of cases for a given area, the formulae published by Cibulskis et al[30] were used (with some additional considerations noted for India in a subsequent section). This approach takes the number of cases to be the mean of higher and lower estimates that each use treatment-seeking and slide positivity rates to adjust the stated number of cases:

$$M = \frac{M_{\text{upper}} + M_{\text{lower}}}{2}$$

Where:

$$M_{\text{upper}} = \frac{C+sU}{rp}$$

$$M_{\text{lower}} = \frac{(C+sU)(1-n)}{rp} = \frac{a(C+sU)}{rp}$$

And:

C - Reported number of confirmed malaria cases in a year.

U - Reported number of unconfirmed malaria cases in a year.

s - Slide positivity rate

r - Reporting completeness

p - The proportion of the population with fever that seeks treatment from health facilities covered by the public reporting system.

n - The proportion of the population with fever that does not seek treatment.

a - The proportion of the population with fever that seeks treatment from any health facility (public and private).

Regarding the variables *p*, *n*, and *a*, the occurrence of fever is taken as a proxy for malaria.[30]

The source data gathered seldom provided the data in a format corresponding directly with the variables in the above formulae. In many cases, interpretation of the data with a predefined set of rules was required to determine appropriate values. These rules are set out in subsequent sections.

1.2.4.2 Calculating the proportion of *P. falciparum* cases from raw data

The above equations relate to non-species-specific API calculations. In order to calculate AFI, these equations need to be used with figures specific to *P. falciparum*. Wherever possible, figures from the primary sources were used to calculate the proportion of *P. falciparum* cases.

However, in many cases, only non-species-specific figures were available. In some cases, the source provided a figure for the proportion of cases that were *P. falciparum*. Often they did not, so national level figures for species breakdowns from the World Health Organization's annual World Malaria Reports for 2016, 2015 and 2013[18,31,32] were obtained.

These were used to derive the proportion of *P. falciparum* malaria in each country and allow the calculation of confirmed and unconfirmed cases of *P. falciparum*.

The figures in these sets of World Malaria Report annexes list the number of *P. falciparum*, *P. vivax*, and other malaria cases, from which a proportion can be calculated. There were two issues:

- The dates covered by the reports overlapped: the 2015 report covered the years 2000–2014 and the 2012 report covered the years 1990–2011. For the period of overlap (2000–2011), the 2015 report figures had precedence. The figures for 2015 were taken from the 2016 report.
- No country had a complete set of species breakdown figures for the entire period.

In summary, if no species-specific case figures were available in the source, the *P. falciparum* cases were calculated by multiplying the total cases by the best available species-proportion figures as follows:

- An explicitly stated proportion/percentage in the source.
- If there was nothing in the source paper, the World Health Organization national species proportions from the annexes of the World Malaria Reports for the year of the source were used.
- If the WHO does not have a national species proportion for the year of the source, the mean species proportion over all years for that country was used.

1.2.4.3 Reported testing regimes

The principle testing regimes reported in sub-national data sources were microscopy tests and rapid diagnostic tests (RDT).
Only one source reported polymerase chain reaction (PCR) figures as a subset of microscopy tests. Since there was a lack of additional PCR results from other sources, the PCR values were not used. The vast majority of reported tests were microscopy.

In a minority of cases, different sources reported conflicting figures for a given admin unit/year combination via microscopy tests versus RDTs.
In these cases, the values from the microscopy tests took precedence over the RDT results.

1.2.4.4 Reported number of confirmed malaria cases in a year (*C*)

Confirmed cases were identified from sources where available and fell into the following six categories, listed in descending order of perceived quality:

Table S4: Confirmed cases fell into the following categories, listed in descending order of perceived quality.

Category of data	Derivation of *P. falciparum* confirmed cases
Species-specific cases confirmed via a testing regime (microscopy, RDT, or a combination of both), with figures of the numbers of tests undertaken and species-specific results.	Confirmed cases were taken directly from the data provided.
Species-specific cases confirmed via a testing regime (microscopy, RDT, or a combination of both) but without providing figures of the total number of examinations.	Confirmed cases were taken directly from the data provided.
Non-species-specific cases confirmed via a testing regime (microscopy, RDT, or a combination of both), with figures of the numbers of tests undertaken and species-specific results.	If the proportion of *P. falciparum* cases is stated in the source, it was used to calculate the number of confirmed cases. If not, the national proportion of *P. falciparum* cases was used for the year of the source. If no national proportion was available for the year, the mean proportion for those years available was used.
Non-species-specific cases confirmed via a testing regime (microscopy, RDT, or a combination of both) but without providing the figures on the number of tests undertaken.	If the proportion of *P. falciparum* cases is stated in the source, it was used to calculate the number of confirmed cases. If not, the national proportion of *P. falciparum* cases was used for the year of the source. If no national proportion was available for the year, the mean proportion for those years available was used. Species-specific cases, without indicating a testing regime or providing raw figures.
Note that an indication of species implies that a test process must have occurred and so the cases are confirmed.	Confirmed cases were taken directly from the data provided.
Non-species-specific cases explicitly stated as confirmed, without indicating a testing regime or providing raw figures. Note the requirement for the figures to have been explicitly stated as confirmed. This is distinct from the data described in the next section, where malaria case figures were provided without indicating they were confirmed.	If the proportion of *P. falciparum* cases is stated in the source, it was used to calculate the number of confirmed cases. If not, the national proportion of *P. falciparum* cases was used. If no national proportion was available for the year, the mean proportion for those years available was used.

The number of confirmed cases was taken as follows:

- If there were just microscopy figures, these were used as the number of confirmed cases.
- If there were just RDT figures, these were used as the number of confirmed cases.

- If there was no indication of the method used, the figures stated as confirmed were taken as the number of confirmed cases.
- If there were microscopy and RDT figures, the total number of confirmed cases was taken as the sum of the microscopy and RDT figures.

The result of the above was a species-specific number of confirmed cases but consideration needed to be taken to exclude cases that were imported.
This depended on the source:

- If the number of cases by microscopy / RDT / unstated method were explicitly stated as being indigenous, they were taken as the final figure for the number of confirmed cases.
- If the number of cases by microscopy / RDT / unstated method were not explicitly stated as being indigenous and the source paper included species-specific figures of imported cases, these imported cases were subtracted from the appropriate species-specific number of confirmed cases to give a final figure for the number of confirmed cases.
- If the number of cases by microscopy / RDT / unstated method were not explicitly stated as being indigenous and the source paper included non-species-specific figures of imported cases, these imported cases were divided into species-specific figures by applying the same proportion of species indicated by the test results.

1.2.4.5 Reported number of unconfirmed cases in a year (*U*)

Many sources provided figures for malaria cases without explicitly stating these were confirmed cases. These figures might be provided in addition to figures for confirmed cases (such as microscopy tests or simply explicitly stated confirmed figures) and were likely cases treated following a presumptive diagnosis based on symptoms. Case figures not explicitly stated to be confirmed were assumed to be unconfirmed. Where additional confirmed cases were provided by a source, these were subtracted from the total case figures to provide a calculated number of unconfirmed cases. Consideration was given to figures for imported cases:

- If the source included figures for confirmed cases, the imported cases were subtracted from those in accordance to the proportion of species in the confirmed cases.
- If the source figures did not indicate any confirmed cases, the imported cases were subtracted from the unconfirmed cases to provide a calculated figure for unconfirmed cases.

Once a figure for unconfirmed cases had been calculated, it was used to calculate an estimate of the number of unconfirmed cases that were *P. falciparum*. This was done using the proportions described earlier in this document.

1.2.4.6 Reporting completeness (*r*)

Reporting completeness represents the number of health facilities reporting relative to the number of reports expected. Sub-national figures for reporting completeness were rare in the government sources collected. Our search only managed to find publicly available sub-national figures for Nepal.
The National Malaria Control Programme in Eritrea also provided figures on request and on condition of keeping these figures confidential.

Sub-national reporting completeness figures are submitted by countries to the World Health Organization as part of the annual data collection exercise for the World Malaria Report. However, the World Health Organization does not have the permission of the countries donating the data to share these sub-national level reporting figures to the research community.

For those countries where sub-national reporting completeness figures were not available, national reporting completeness values derived from the World Health Organizations' 2015 World Malaria Reports were used.[18]

Most countries only had figures for between three and eight years. These figures were not necessarily for consecutive years. To fill in the gaps between years, a mean value of all available years was used. To deal with the missing data prior to the earliest year for which a figure was available, the earliest reporting completeness available was assigned to one of the following bands:

- > 80%
- 50%-80%
- < 50%

The values for the missing earlier years were then calculated using a linear progression back to the base of the reporting completeness band the figure fell into. For example, if the earliest year for which data was available was 2005 and this figure was 65% reporting completeness, the years 2000 to 2004 would be calculated by decreasing the reporting completeness by even steps from 65% to 50% for the years 2004 to 2000 (with each year 1·5% less than the previous one): The justification for this is the assumption that reporting completeness has improved over time. For the < 50% band, a floor of 10% reporting completeness was assumed.

1.2.4.7 Slide Positivity Rate (*s*)

Slide Positivity Rate is defined as the number of microscopy slides positive for malaria divided by the total number of slides examined. For the purposes of calculating AFI, the SPRPf was required (i.e. the number of slides positive for *P. falciparum* divided by the total number of microscopy examinations undertaken).

Where figures were available in the source for the total number of examinations undertaken and the resultant slide-positives for *P. falciparum*, these were used to calculate SPRPf. Where the number of examinations and slide-positivity figures were available but did not specify a species, SPR was calculated and then multiplied by the best available figure for the proportion of *P. falciparum*.

If the source did not have slide examinations and slide-positivity figures available, national figures were used instead.
The national figures were derived from the slide figures in the appendices of the World Health Organization's annual World Malaria Reports for 2016, 2015 and 2012[18,31,32] .
Because the data in these reports overlaps, the data in the most recent report took precedence over the earlier ones.

No country reported slide figures for the entire period under research, so a mean value was calculated for each country based on the years for which there were figures. This mean value was then used for the missing years for the country.

1.2.4.8 Population figures

Most of the sub-national case data collected from Ministry of Health reports had associated population figures. However, while these population figures were collected, they were disregarded in favour of figures provided by IHME because of the latter's provenance to the UN. For the purposes of calculating AFI, the population-at-risk was used as the denominator (see above).

1.2.4.9 Treatment-seeking figures (*p*, *n*, *a*)

Treatment-seeking rates are estimated as the proportion of children under five with fever that were taken to treatment (see section "Treatment-seeking model"). These values were used as representative of the population as a whole. [21]

These figures provide an upper, lower, and mean figure for each of:

- The proportion of children under five with fever seeking treatment from health facilities covered by the public reporting system.
- The proportion of children under five with fever seeking treatment from any health facility (public and private).

To reflect the uncertainty in the reliability of the data sources collected, the following assignments were made to the AFI calculations to reflect -the widest possible range of estimates.

For the calculation of M_{upper}:

p was set to the lower estimate of the proportion of children under five with fever seeking treatment from health facilities covered by the public reporting system. The formula for the upper cases estimate adjusts for cases being missed due to persons with fever who did not seek treatment from the public healthcare system: both those who sought treatment from the private sector, and those who sought no treatment. Using the lower estimate for this proportion amplifies the estimated number of fevers omitted from the public sector reporting and, accordingly, the

upper cases estimate by the largest amount. The upper cases estimate assumes the same slide positivity rate, s, among those who do and do not seek treatment.[30]

For the calculation of M_{lower}:

p was set to the upper estimate of the proportion of children under five with fever seeking treatment from health facilities covered by the public reporting system. *a* was set to the lower estimate of the proportion of children under five with fever seeking treatment from any health facility (public and private).

The formula for the lower cases estimate adjusts for cases missed by persons with fever who did not seek treatment from the public healthcare system. The upper estimate for public treatment-seeking is used, since this provides the most optimistic view of treatment-seeking from the public healthcare system, and therefore of the completeness of the reported figures from the public sector. This reduces the number of fevers to be added to those from the public sector, and therefore the lower cases estimate.

By the same logic, the lower estimate for any treatment-seeking is used since, in combination with the upper public treatment- seeking estimate, this minimises the number of persons with fever estimated to have sought treatment within the private sector. Multiplying the slide positivity rate (*s*) used to adjust the unconfirmed cases by the proportion of persons with fever who sought any treatment applies the assumption that only fever cases that sought treatment had malaria and those that did not seek treatment had no malaria ($s = 0$).

1.2.4.10 Special considerations for India

A large number of malaria cases in India are treated in the private sector and hence go unreported by the HMIS.[33] The figures for cases and microscopy examinations in the appendices of the World Health Organization's World Malaria Reports are those reported in the public sector and are therefore not representative of the total case burden in the country. Furthermore, the World Malaria Report figures represent data from both passive case detection (PCD: i.e. people presenting at hospital with symptoms) and active case detection (ACD: i.e. public healthcare going and screening everyone in a village).[33]

This means that although we can calculate a slide positivity rate (SPR) from the figures of slides positive/slides examined in the public sector (published in the World Malaria Reports), we have no way of knowing what the SPR is for ACD or PCD – it is just a combined figure. The SPR for PCD will be higher than that for ACD – people are detected by PCD when they go to a hospital because they are ill. The ACD rates are based on active screening of fevers in the community meaning the febrile denominator population is likely to be larger than the subset that may seek care.

The World Malaria Reports include figures for the high, low, and mean case estimates for countries for selected years, based on the formulae discussed in section 2.1. The inputs for these estimates are the cases and number of slides reported in the appendices and unpublished treatment-seeking estimates calculated from Measure DHS (http://www.dhsprogram.com/) survey results.[33]

However, because the World Malaria Report figures for India do not include the totals for the significant number of cases treated in the private care sector and it is impossible to determine an appropriate value for SPR because of the high levels of ACD in the public sector, a different approach is taken for India for the high, low, and mean estimates in the World Malaria Reports. [33] Estimates of the number of private sector cases and cases where no treatment was sought are calculated by scaling up the public cases figures by the ratio of private treatment-seeking rates to public treatment-seeking rates and non-treatment-seeking rates to public treatment-seeking rates respectively.

Because all cases in the private sector are passively detected, the private figures have to be modified upwards again by a factor to reflect that the PCD SPR is unknown.

This factor is also applied to the non-treatment-seeking cases. The World Health Organization do this using unpublished figures from the private sector that are not available publically.[33]

In order to provide credible national case figures for India, we adopted the same approach as the World Health Organization, taking the following steps:

- The low, high, and mean case estimates for India published in the 2015, 2016 and 2017 World Malaria Reports[18,32,34] were taken as anchor points. The years for which there are estimates are 2000, 2005, 2010, 2012, 2013, 2014, 2015 and 2016.

- The public sector case figures from the World Malaria Reports and treatment-seeking estimates (see section “Treatment-seeking model”) were used to estimate private sector cases and non-treatment-seeking cases for each of the above years.
- The private and non-treatment-seeking cases were then scaled up by adjusting the SPR applied to them: the SPR was increased until the sum of reported public sector cases, estimated private sector cases, and estimated non-treatment-seeking cases approximated the point estimates published in the 2015, 2016 and 2017 World Malaria Reports[18,32,34].
  We could not use the actual SPR adjustments used by the World Health Organization in the World Malaria Reports because these are unpublished.
- The final national figures used by MAP were therefore the sum of reported public sector cases, estimated private sector cases scaled up by a modified SPR, and estimated non-treatment-seeking cases scaled up by a modified SPR.

1.2.4.11 Special Considerations for countries in Elimination Phase

Countries classified by the World Health Organization as being in “Malaria Elimination Phase” (i.e. API < 0·001) had their data treated as follows for the years they were in elimination phase:

- The reporting completeness was taken as 1 (i.e. 100% of cases reported), regardless of the reporting completeness reported by sources.
- The treatment-seeking values were taken to be 1 (i.e. 100% of patients seek treatment via the public healthcare system), regardless of the modelled treatment-seeking rates
- All reported case figures were treated as confirmed, regardless of whether the sources explicitly reported them as such.

Countries classified by the World Health Organization as being in “Pre-Elimination Phase” (i.e. API < 0·005) received no additional treatment during the calculation of AFI.

1.2.4.12 Outlier removal

Outlier removal was performed by two processes. At the national level, data were examined and removed if they were considered unreliable. Unreliability was inferred by large changes year to year within a country and comparisons to other published estimates. The table of national data exclusions is given in Table S5.
A further outlier rule was applied to subnational data. Data were removed if they either had an AFI of > 600; or had an AFI of > 100 and a population at risk of less than 10% of its population. Indian subnational units were exempt from this rule. Additionally, this exclusion pertains only to areas modelled using the surveillance method (i.e., generally lower burden). In practice this outlier exclusion, which was rarely used and only applied to low administrative levels (e.g., admin level 3), occurred when large case numbers were attributed to small administrative units with low populations. The most common interpretation for such instances was that a medical facility (at which cases were counted) was located within the admin unit, and this facility attracted cases from neighbouring areas (thus inflating the apparent APIs). Because high-resolution data on treatment seeking and migration behaviour did not exist we were unable to estimate catchments areas beyond the administrative unit boundaries. Truncated probability distributions could have been used in the likelihood to mitigate this issue without outliering the data, but that approach would also have required an arbitrary threshold for truncation while also introducing computational issues in implementation. As a result, we were limited to this practical restriction.

1.2.4.13 Post-hoc masking

This step creates a raster cube that indicates where pixels should be exactly zero.

Any administrative unit, year pair with zero AFI is included in the mask (i.e. set to zero) unless:

Table S5: National level data exclusion years.

Country	Excluded year(s)
Afghanistan	1994
Bangladesh	< 2000
Belize	1990–1999
Bolivia	1990–1999
Botswana	< 1990
Brazil	< 1990
Bhutan	< 1998
Colombia	< 1990
Comoros	< 2004
Djibouti	< 1990
Algeria	< 2000
Eritrea	< 1990
French Guiana	< 1990
Guyana	< 1990
Haiti	1993, 1999, 2002, 2003
Indonesia	< 2000
India	All but 2000, 2005, 2010, 2012-2016
Iran	< 1990
Kyrgyzstan	< 1990
Cambodia	< 1990
South Korea	< 2000
Laos	< 1990
Sri Lanka	< 1990
Mexico	< 1990
Myanmar	< 1990
Namibia	< 2000
Oman	< 1991
Pakistan	< 2000
Peru	< 2000
Papua New Guinea	< 2000
Paraguay	< 1990
Solomon Islands	< 2008
Sao Tome Principe	1982
Suriname	< 2000
Swaziland	< 2000
Tajikistan	< 2000
East Timor	1998
Tanzania	2016
Vietnam	< 2000
Yemen	< 2001
South Africa	< 2000

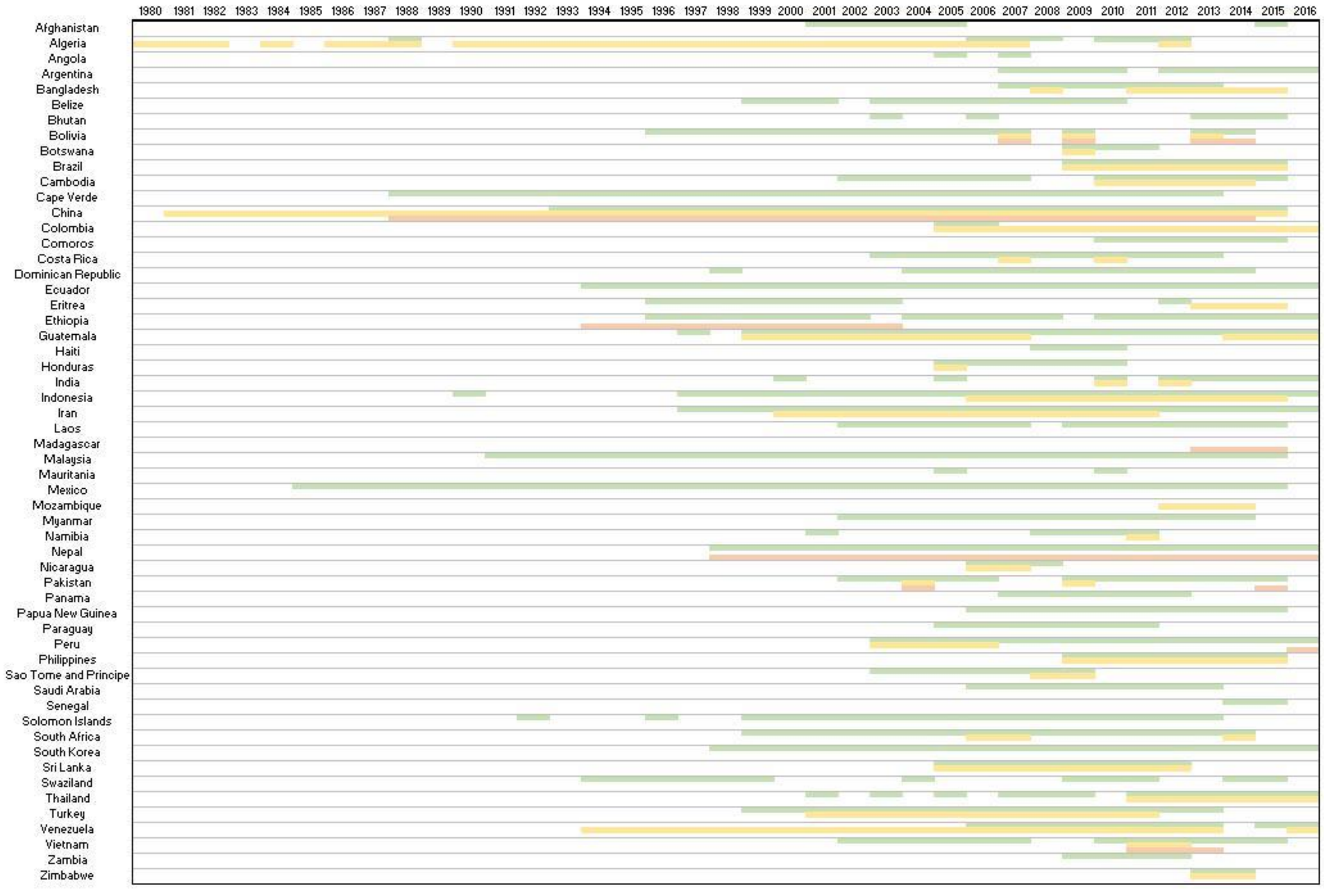

Figure S2: API data availability at different admin levels and years.

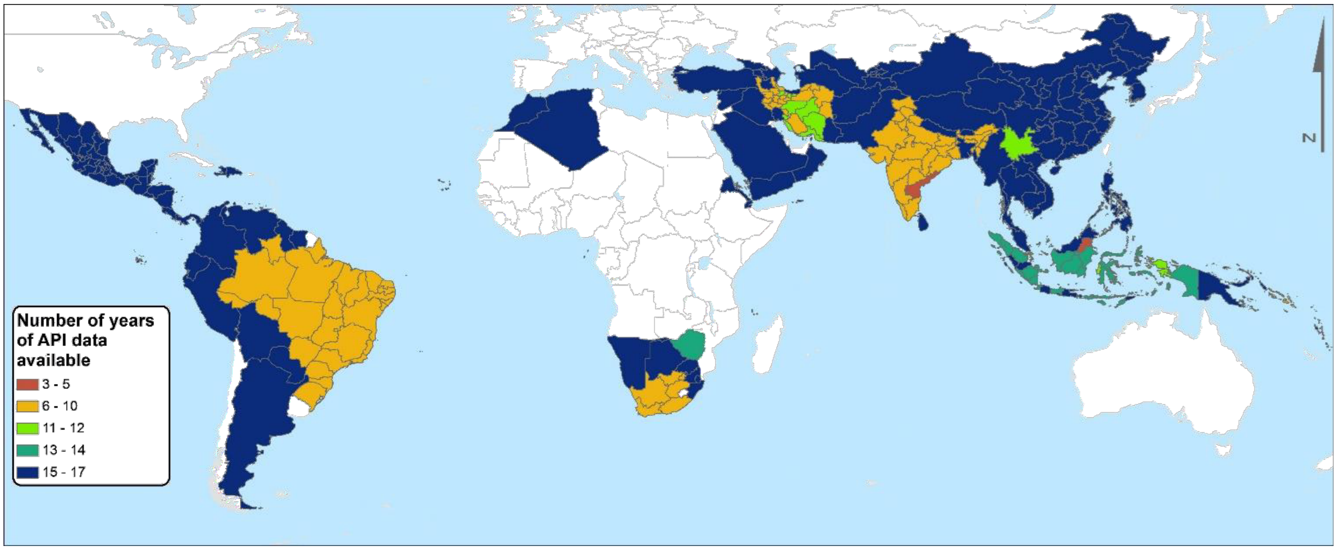

Figure S3: API data availability (2000-2017) at admin levels used for time series modelling.

- The zero is due to a population at risk value of zero.
- A child administrative unit (i.e. a smaller administrative unit fully contained by the large unit) has a non-zero AFI value.

Furthermore, if a unit is included in the mask and all later data in that unit is zero AFI or missing, the mask is extrapolated forward in time. This is particularly needed because many areas stop reporting malaria cases once they have reached elimination.

For example:

If Goa had a zero AFI in 2011 and 2014 and an AFI of 0.1 in 2015, then the algorithm would:

- Do nothing.

If Goa had a zero AFI in 2011 and 2014 and nothing for 2015, then the algorithm would:

- Do nothing until 2011.
- Add 2011 to the mask, since it is reported as zero cases.
- Add 2012-2015 to the mask, since the most recent previous value was a zero, and there are no future values.

If Goa had a zero AFI in 2011 and 2014 and a zero AFI for 2015, then the algorithm would:

- Do nothing until 2011.
- Add 2011 to the mask, since it is reported as zero cases.
- Add 2012-2014 to the mask, since the most recent previous value was a zero, and the next reported value in 2015 was zero.
- Add 2015 to the mask, since it is reported as zero cases.

Finally, all administrative unit, year pairs that are included in the mask are combined with the environmental limits to create a raster cube mask.

1.2.5 Outside of Africa: time-series models

For estimating malaria incidence outside of Africa, we first fitted time-series models to national API data. These time-series estimates were then included as data in the subsequent disaggregation regression models.

1.2.5.1 National time-series

The basic model for a national time-series includes short term (ST) and long term (LT) moving average elements to capture short range and long range variation. In hierarchical Bayesian notation, the API for country *i* in year *j* is modelled as:

$$
\begin{aligned}
API_{i,j} &\sim \begin{cases} N(\mu_{i,j}, \sigma_{i,j}^2) \\ N(\mu_{i,j}, (0.001\mu_{i,j} + 0.001)^2) & \text{for fixed data.} \end{cases} \\
\log(API_{i,j}) &= \begin{cases} \text{offset}_i, & \text{for } j = 1, \\ \text{offset}_i + \sum_{l=1}^{p} \beta_l X_{l,i,j} + w_{i,j-1}^{(ST)} + w_{i,j-1}^{(LT)} & \text{for } j > 1. \end{cases} \\
\text{where } w_{i,j}^{(ST)} &= \frac{\sum_{k<j} \exp(-\frac{(j-k)^2}{0.5^2}) \epsilon_{i,k}^{(ST)}}{\sum_{k<j} \exp(-\frac{(j-k)^2}{0.5^2})}, \\
w_{i,j}^{(LT)} &= \frac{\sum_{k<j} \exp(-\frac{(j-k)^2}{2.5^2}) \epsilon_{i,k}^{(LT)}}{\sum_{k<j} \exp(-\frac{(j-k)^2}{2.5^2})}, \\
\epsilon_{i,j}^{(ST)} &\sim N(0, \tau_i^2 * \rho), \\
\epsilon_{i,j}^{(LT)} &\sim N(0, \tau_i^2 * (1-\rho)), \\
\text{logit}(\rho) &\sim \text{Uniform}(-\infty, \infty), \\
\log(\tau_i^2) &\sim N(\gamma_i, \zeta_i^2), \\
\gamma_i &\sim N(-4,1), \\
\log(\zeta_i^2) &\sim N(-4,1), \\
\text{offset}_i &\sim N(0,10), \\
\text{and } \beta_l &\sim N(0,1).
\end{aligned}
$$

Here, $\mu_{i,j}$ and $\sigma_{i,j}^2$ denote the API mean and standard deviation from the API data. The latter is calculated using the upper and lower bounds of the API estimates.

$\{X_{l,i,j}\}$ represents the lth covariate for country i in year j. The covariates considered are the gap-filled World Bank indices:

1. Health expenditure, total (% of GDP).
2. Immunization, DPT (% of children ages 12-23 months).
3. GDP growth (annual %).
4. Pregnant women receiving prenatal care (%).
5. Primary completion rate, total (% of relevant age group).
6. Health expenditure, public (% of GDP).

The covariates are normalised before testing and only significant covariates with negative coefficients are used in the final model.

The above model is used for countries with large amounts of data over the study period. For countries with many missing values, we borrow information from countries in the same region by setting the long-term moving average ($w^{(LT)}$) as a shared regional trend. IHME superregions, which are based on epidemiological similarity and geographic closeness, were used as regions. If there are conflicting trends within the IHME superregions (e.g. increasing versus decreasing), countries with similar trends were grouped and treated as separate regions.

Note that the basic model presented above cannot predict zero API values. To account for zeros especially in low API settings, we introduce a Tobit factor. If $\log(API_{i,j}) < c$, where c is the smallest $\log(API)$ value corresponding to a non-zero API value for that country, we set the API estimate to zero.

The models were defined with the R package Template Model Builder (TMB)[35] and optimised in R. Once a model has been fit, we generate 1000 realisations of the API time series using the posterior distributions of the estimated parameters. These enable us to create 95% credible intervals.

1.2.5.1.1 Special case: Sri Lanka

For Sri Lanka, we replace the moving average terms in time-series model by an autoregressive model of order one (AR(1)). In the hierarchical Bayesian formulation, we have, in place of $w_{i,j}^{(ST)}$, $w_{i,j}^{(LT)}$ and their parameters:

$$\begin{aligned} w_j &= \phi w_{j-1} + \epsilon_j \\ \text{where } \epsilon_j &\sim N(0, \sigma^2), \\ \tau^2 &= \frac{\sigma^2}{1-\phi^2}, \\ \log(\tau^2) &\sim N(\gamma, \zeta^2), \\ \gamma &\sim N(0,1), \\ \log(\zeta^2) &\sim N(0,1), \end{aligned}$$

and the correlation parameter $|\phi| < 1$. This AR(1) model gives a better fit for the Sri Lanka data than a regional model with other Southeast Asian countries, resulting in narrower credible intervals.

1.2.5.2 Subnational time-series

The models for subnational time-series modelling in subnational units of Brazil, China, Indonesia, India, Iran and Mexico are based on the regional time-series model. (Kenya and Ethiopia use the cartographic approach and aggregate administrative level API via the pixels.) Now, instead of countries in one region sharing a LT moving average, subnational units in one country share the LT moving average.

The main difference between the national models and the subnational models is that for the latter, the modelled subnational (ADMIN1) counts need to add up to the national count where we have national API data. That is, for subnational unit i in year j, we require that:

$$\begin{aligned} Count_{national,j} &= \sum_i Count_{i,j} \\ \text{where } Count_{national,j} &= \frac{API_{national,j}}{1000} \times Population_{i,j}, \\ \text{and } Count_{i,j} &= \frac{API_{i,j}}{1000} \times Population_{i,j}. \end{aligned}$$

Model variants involve the Tobit factor and omitting unit-specific ST moving averages.

For units where we have sufficient years with both subnational and national data, we proportionally adjust the subnational API data so that the subnational counts add up to the national count so as to help the model convergence ("prop_adj" = "Yes").

Instead of the World Bank covariates, we use analogous IHME covariates which are available at the ADMIN1 level:

1. Fraction of children born in a given country-year who have received 3 doses of DPT3.
2. GDP per capita (with 2010 as the base year and in international dollars).
3. Proportion of pregnant women receiving any antenatal care from a skilled provider.
4. Proportion of pregnant women receiving 4 or more antenatal care visits including 1 or more from a skilled provider.
5. Age standardized educational attainment for males.
6. Age standardized educational attainment for females.
7. Education in years per capita for males.
8. Education in years per capita for females.

As before, the covariates are normalised before testing and only significant covariates with negative coefficients are used in the final model.

Additional covariates used are:

9. Mean national modelled API.

10. Indicator for the time period after 2012.
11. Indicator for the time period after 2013.
12. Indicator for the time period before 2000.

Note that we do not model subnational units without API data or which only have data zeros but set their API values to zeros since we do not have data to suggest otherwise (“zeros” = “Yes”).

If the national trend aggregated from the subnational time series differs significantly from that suggested by the national model, we proportionally adjust the subnational model draws using the national model draws (“draws” = “Yes”). This approach can be seen as using the subnational models to model the changes in the subnational proportions intead. Alternatively, we considered using the means of the national API estimates from the national model as a covariate. Note that a sensible coefficient sign for this would be positive.

The details for the final models for the national and subnational models can be found in Tables S6, S7.

1.2.5.2.1 Special case: India

The API data for Indian ADMIN1 units have different features (fluctuating, steady decrease, drops in different years) which are difficult to capture with one subnational model. Instead, we group the units according to these features and model these ‘clusters’ separately. Since the clusters also have different degree of API variability from year to year, we allow the bandwidth of the moving averages (“st_bw”) to vary between clusters. Since the subnational API data have been adjusted proportionally against the national API data, we can aggregate the subnational API estimates (via counts) to get the final India national API estimates (“aggr.” = “Yes”).
To get the India urban and rural time series we split the state level time series based on population densities. For each admin unit, the population density of urban and rural portions are calculated from the gridded population raster and IHME shapefiles that define urban and rural boundaries. The population densities are then linked to incidence rate via an empirical relationship. These urban and rural incidence rates are subsequently scaled, taking population into account, such that they are consistent with incidence rate for the entire admin unit.

Table S6: National time-series model variant descriptions.

Model	Long-term trend	Short-term trend	Covariates	Tobit	Comments
pf	Country-specific	Country-specific	No	No	Trends governed by moving averages of differing smoothness. (Original model since no significant covariates upon fitting.)
rt	Region-shared	Country-specific	Yes	No	LT trend acts as averaging between countries but favours those with more data; ST trend accounts for remaining variability in country.
nc	Region-shared	Country-specific	No	No	
simple_tobit	Region-shared	Country-specific	Yes	Yes	Treat the log(API) as a latent process and set a cut-off based on the smallest positive value observed. Predictions under cut-off are set to zero
simple_tobit_nc	Region-shared	Country-specific	No	Yes	
standalone_tobit	Country-specific	Country-specific	No	Yes	
standalone_tobit_c	Country-specific	Country-specific	Yes	Yes	
pf_ar	Country-specific	Country-specific	Yes	No	The moving average terms are replaced by an AR(1) series.

Table S7: National time-series model variants by country.

country	national_region	model	covariates
Bolivia	ALA	nc	NA
Ecuador	ALA	nc	NA
Paraguay	ALA	nc	NA
Peru	ALA	nc	NA
Brazil	AmLA	nc	NA
Colombia	AmLA	nc	NA
French Guiana	AmLA	nc	NA
Guyana	AmLA	nc	NA
Suriname	AmLA	nc	NA
Argentina	Bespoke	standalone_tobit_c	2
Sri Lanka	Bespoke	pf_ar	6
Belize	CCLA	nc	NA
Costa Rica	CCLA	nc	NA
Guatemala	CCLA	nc	NA
Mexico	CCLA	nc	NA
Nicaragua	CCLA	nc	NA
Armenia	CEECA	simple_tobit_nc	NA
Azerbaijan	CEECA	simple_tobit_nc	NA
Georgia	CEECA	simple_tobit_nc	NA
Kyrgyzstan	CEECA	simple_tobit_nc	NA
Uzbekistan	CEECA	simple_tobit_nc	NA
China	EAsia	nc	NA
North Korea	EAsia	nc	NA
South Korea	EAsia	nc	NA
Tajikistan	EAsia	nc	NA
Djibouti	ESA 1	rt	1, 4, 5, 6
Eritrea	ESA 1	rt	1, 4, 5, 6
South Sudan	ESA 1	rt	1, 4, 5, 6
Yemen	ESA 1	rt	1, 4, 5, 6
Comoros	MCM	rt	3, 4, 6
Madagascar	MCM	rt	3, 4, 6
Mayotte	MCM	rt	3, 4, 6
Algeria	NAME	rt	5
Iran	NAME	rt	5
Oman	NAME	rt	5
Saudi Arabia	NAME	rt	5
East Timor	Oceania	nc	NA
Vanuatu	Oceania	nc	NA
Bangladesh	SA	rt	4, 5
Bhutan	SA	rt	4, 5
India	SA	rt	4, 5
Nepal	SA	rt	4, 5
Pakistan	SA	rt	4, 5
Cambodia	SEAsia	rt	1, 4, 5, 6
Indonesia	SEAsia	rt	1, 4, 5, 6
Laos	SEAsia	rt	1, 4, 5, 6
Myanmar	SEAsia	rt	1, 4, 5, 6
Papua New Guinea	SEAsia	rt	1, 4, 5, 6
Solomon Islands	SEAsia	rt	1, 4, 5, 6
Vietnam	SEAsia	rt	1, 4, 5, 6
Botswana	SSA	rt	1, 4, 5
Namibia	SSA	rt	1, 4, 5
South Africa	SSA	rt	1, 4, 5
Swaziland	SSA	rt	1, 4, 5

country	national_region	model	covariates
Afghanistan	Standalone	pf	NA
Cape Verde	Standalone	pf	NA
Dominican Republic	Standalone	pf	NA
El Salvador	Standalone	pf	NA
Haiti	Standalone	pf	NA
Honduras	Standalone	pf	NA
Malaysia	Standalone	pf	NA
Morocco	Standalone	pf	NA
Panama	Standalone	pf	NA
Philippines	Standalone	pf	NA
Syria	Standalone	pf	NA
Thailand	Standalone	pf	NA
Turkmenistan	Standalone	pf	NA
Venezuela	Standalone	pf	NA
Iraq	Standalone Tobit	standalone_tobit	NA
Turkey	Standalone Tobit	standalone_tobit	NA
Guinea	WSA 1	rt	6
Guinea-Bissau	WSA 1	rt	6
Liberia	WSA 1	rt	6
Sao Tome and Principe	WSA 1	rt	6
Sierra Leone	WSA 1	rt	6

Table S8: Subnational time-series model variant descriptions.

Model	Long-term trend	Short-term trend	Covariates	Tobit	Comments
pf	Country-shared	ADMIN1-specific	Yes	No	ADMIN1 units are modelled as a region (that is the country) similar to rt of the national models; the corresponding national API is calculated from the sum of the subnational counts.
pf_nrt	No	ADMIN1-specific	Yes	No	ADMIN1 units are modelled as individual units whose count sum is used to calculate the national API.
pf_nrt_nc	No	ADMIN1-specific	No	No	
pf_nsubt	Country-shared	No	Yes	No	
pf_nsubt_nc	Country-shared	No	No	No	

Model	Long-term trend	Short-term trend	Covariates	Tobit	Comments
pf_onec	Country-shared	Yes	1	No	
pf_nsubt_onec	Country-shared	No	1	No	
pf_nc	Country-shared	ADMIN1-specific	No	No	
pf_tobit	Country-shared	ADMIN1-specific	Yes	Yes	ADMIN1 units are given a latent log(API) process and cut-off (based on the smallest positive value) to be able to account for and predict data zeros.
pf_tobit_nc	Country-shared	ADMIN1-specific	No	Yes	
cluster_sep	No	ADMIN1-specific	Yes	No	This model is the same as pf_nrt but allows the bandwidth of the moving averages to be varied more easily.

Table S9: Subnational time-series model variants by country.

country	subnational_units	prop_adj	zeros	model	covs	st_bw	draws	aggr.
Brazil	All	Yes	No	pf_nsubt	4, 5, 7	0.5	No	No
China	All	Yes	Yes	pf_tobit_nc	NA	0.5	No	No
Indonesia	All	No	No	pf_nrt_onec	9	0.5	Yes	No
India	Andhra Pradesh, Bihar, Chhattisgarh, Gujarat, Haryana, Jharkhand, Meghalaya, Mizoram, Odisha, Telangana, Tripura	Yes	No	cluster_sep	4, 5	1.2	No	Yes
India	Arunachal Pradesh, Assam, Kerala, Madhya Pradesh, Maharashtra, Manipur, Nagaland, Sikkim, West Bengal, The Six Minor Territories	Yes	No	cluster_sep	1, 2, 6	1	No	Yes
India	Himachal Pradesh, Punjab, Uttarakhand, NCT of Delhi	Yes	No	cluster_sep	6, 10	1.8	No	Yes
India	Jammu and Kashmir, Tamil Nadu, Uttar Pradesh, Rajasthan, Goa, Karnataka	Yes	No	cluster_sep	6, 10	1.5	No	Yes
Iran	All	No	No	pf	2, 8	0.5	No	No
Mexico	All	Yes	Yes	pf_tobit_nc	NA	0.5	Yes	No

1.2.6 Outside of Africa: disaggregation regression

1.2.6.1 Machine learning with PR points

In order to incorporate information from parasite prevalence surveys, we fitted a number of machine learning models to PR survey data and environmental covariates. Models were fitted using the ‘caret’ package.[36] We then predicted these models globally. The new predicted surfaces were then used as covariates in later models.

We used all data in the MAP *Pf* PR database as described above. The prevalence proportion was converted to incidence rate using the prevalence-incidence model.

1.2.6.1.1 Model validation

5-fold cross validation was used to select hyperparameters and measure model accuracy. For each model, grid search was used to create candidate hyperparameter sets. That is, for each hyperparameter a number of values were chosen and then models were fitted with all possible combinations of these values. Root mean squared error was used as the metric of model accuracy.

1.2.6.1.2 Models and hyperparameters

We fitted a number of regression models to predict incidence from the set of covariates. The models fitted were elastic nets,[37] random forests,[38] k-nearest neighbour, robust linear models,[39] gradient boosted tree[40], and neural networks.[39] As the predictions from these models are to be used as covariates by later models, models with uncorrelated predictions are the most useful. These models were selected as they cover a number of underlying model structures (tree-based methods, nearest neighbour methods, linear models, neural networks) which gives them the best chance at making uncorrelated predictions. Finally, we selected a 5 models by fitting an elastic net model using prevalence as the response variable and out-of-sample predictions from the models as covariates. The alpha parameter (fraction of LASSO penalty vs ridge penalty) was set to 0.05. As the ridge penalty is strong, this will penalise correlated covariates, while including the LASSO penalty allows the model to force coefficients to exactly zero and therefore perform covariate selection.

1.2.6.2 Disaggregation regression

1.2.6.2.1 Data

The response data is malaria incidence rate (per person per year) for an associated spatial polygon. The data comes from two sources.

The first set of data comes from time-series models fitted previously. These time-series models are fitted to data at the national level and to the ADMIN1 level for a select few countries (Brazil, China, India, Indonesia, Iran, Mexico, and South Africa). In the case of India, each state is split into two, a rural polygon and an urban polygon. These are the results published as part of the GBD2017 study.[41] Therefore, the results here are required to exactly match these results. As the data are from a time-series model, they are complete and have associated uncertainty estimates. In the subsequent disaggregation modelling, these datapoints are considered equally whether they are at the national or subnational level and are referred to collectively as “ADMIN0”.

The second set of data are any additional subnational incidence rate data (sub-ADMIN1 for Brazil, China, India, Indonesia, Iran, Mexico, and South Africa). These data have upper and lower bounds based on M_{upper} and M_{lower} as described in section “AFI formulae”. The mean of the upper and lower bound is used as the point estimate of incidence. The polygons associated with these data range from ADMIN1 to ADMIN3 levels. They form a hierarchy with each polygon being considered a child of the larger polygon that it is within.

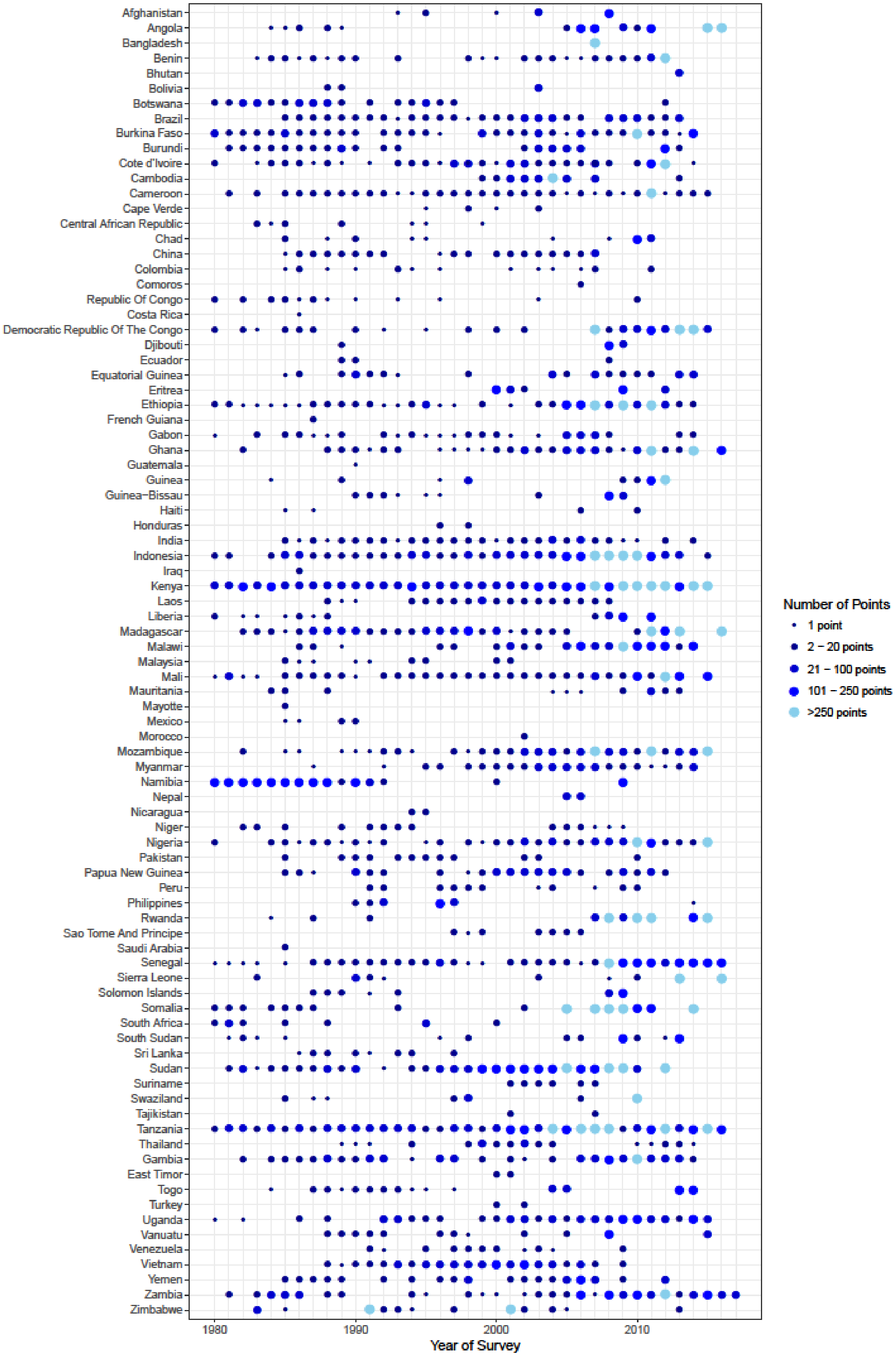

Figure S4: *Pf*PR global data summary.

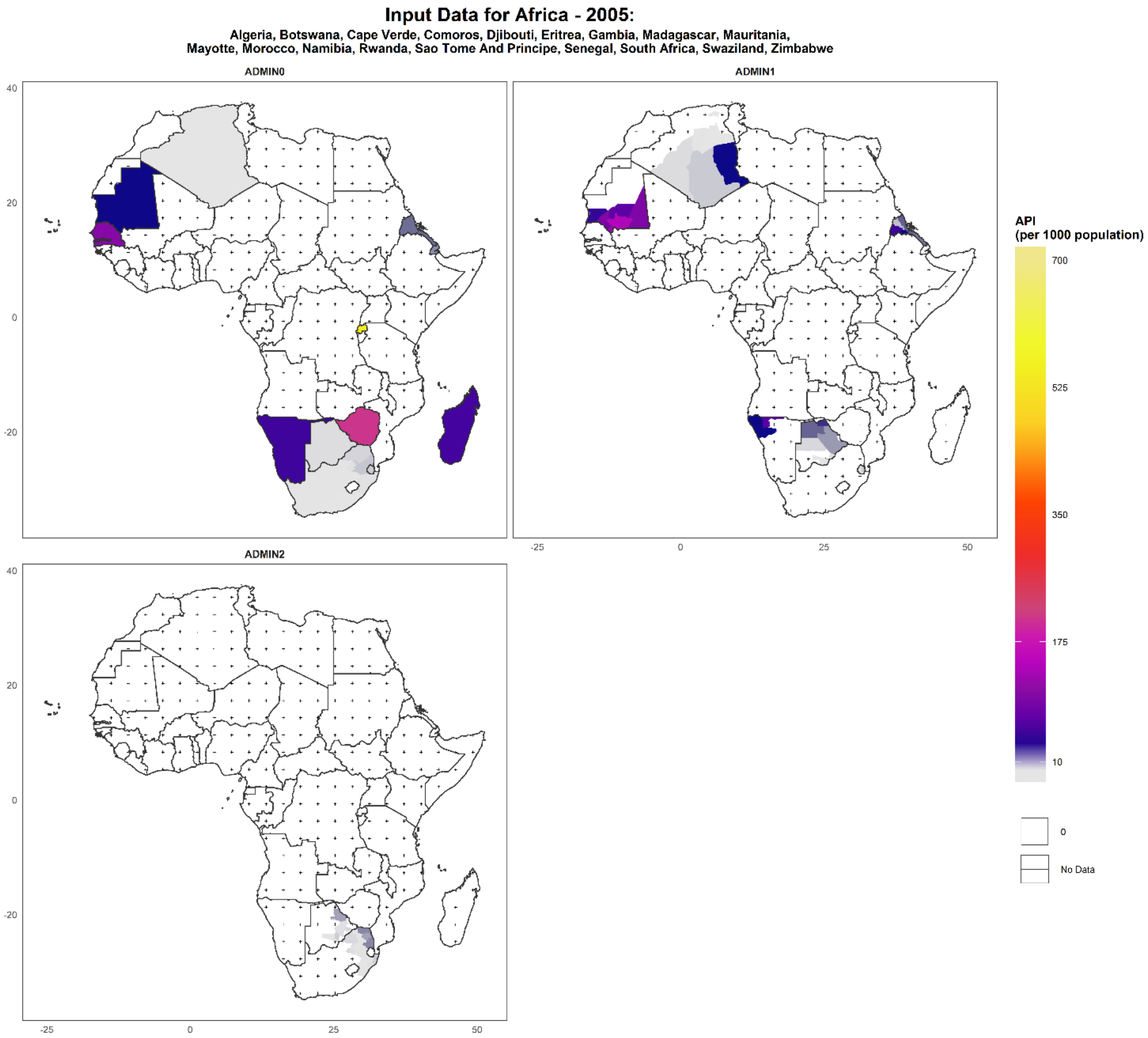

Figure S5: Africa 2005 input data facetted by admin level.

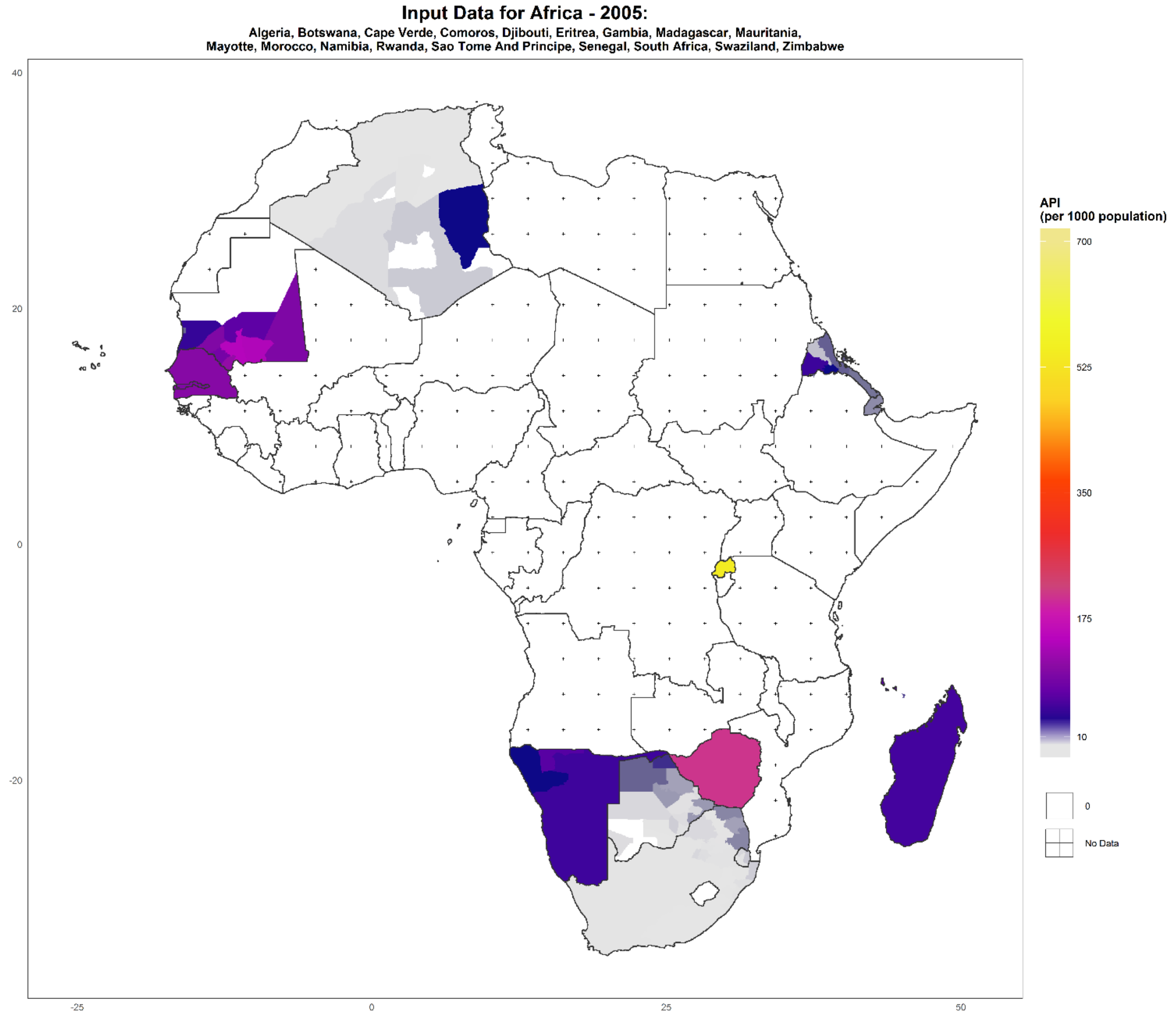

Figure S6: Africa 2005 input data overplotted such that lower admin level data is on top.

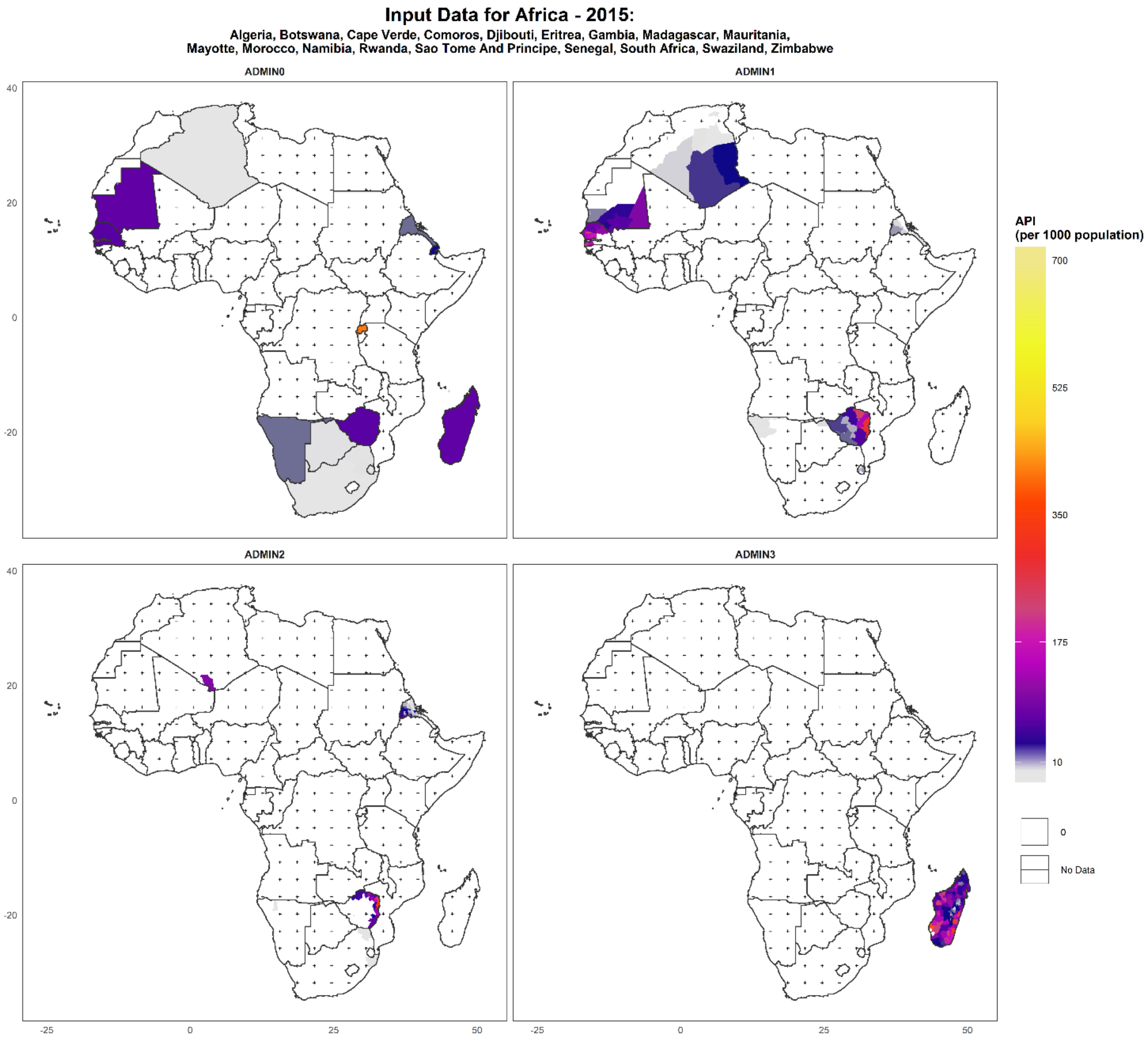

Figure S7: Africa 2015 input data facetted by admin level.

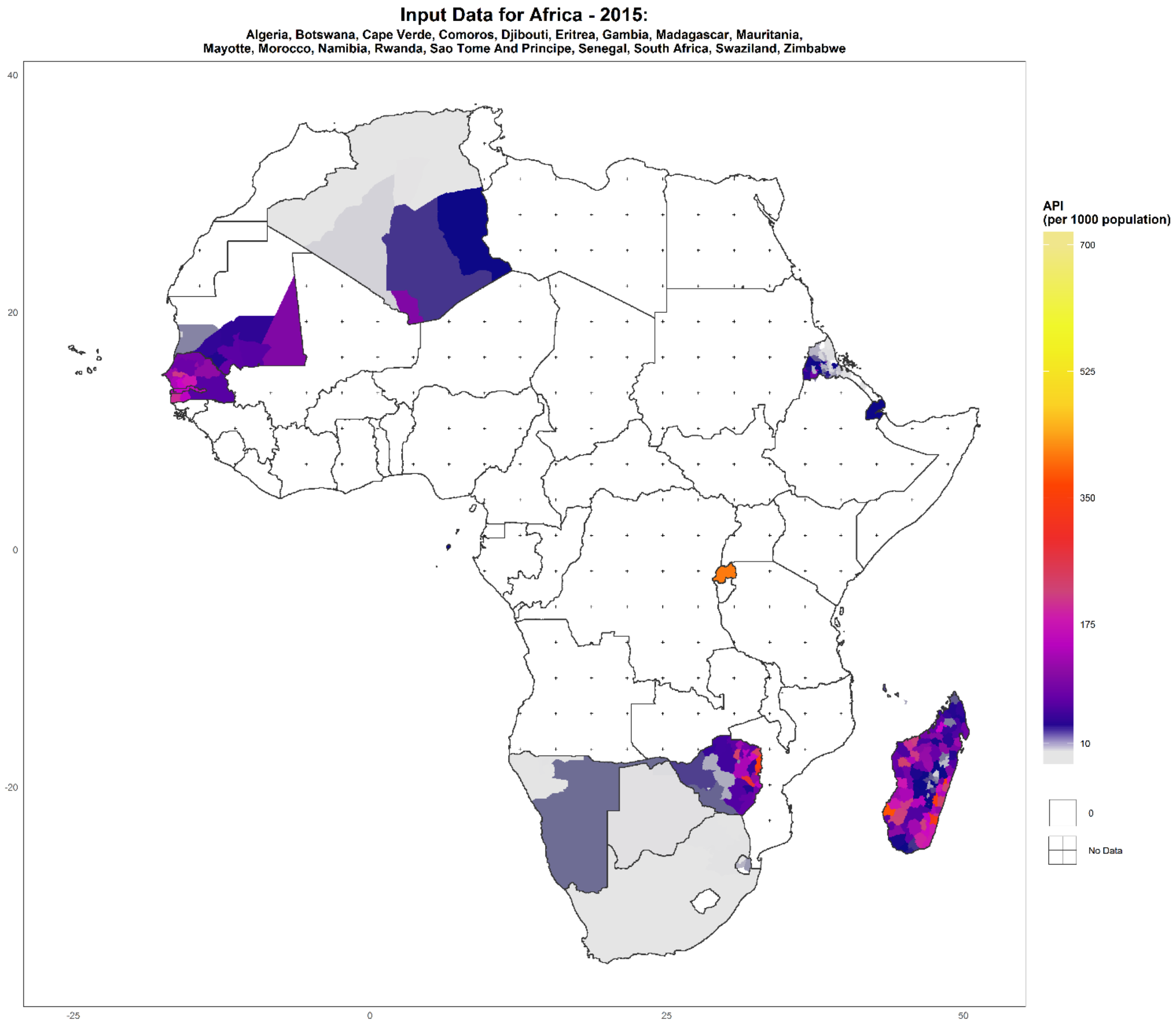

Figure S8: Africa 2015 input data overplotted such that lower admin level data is on top.

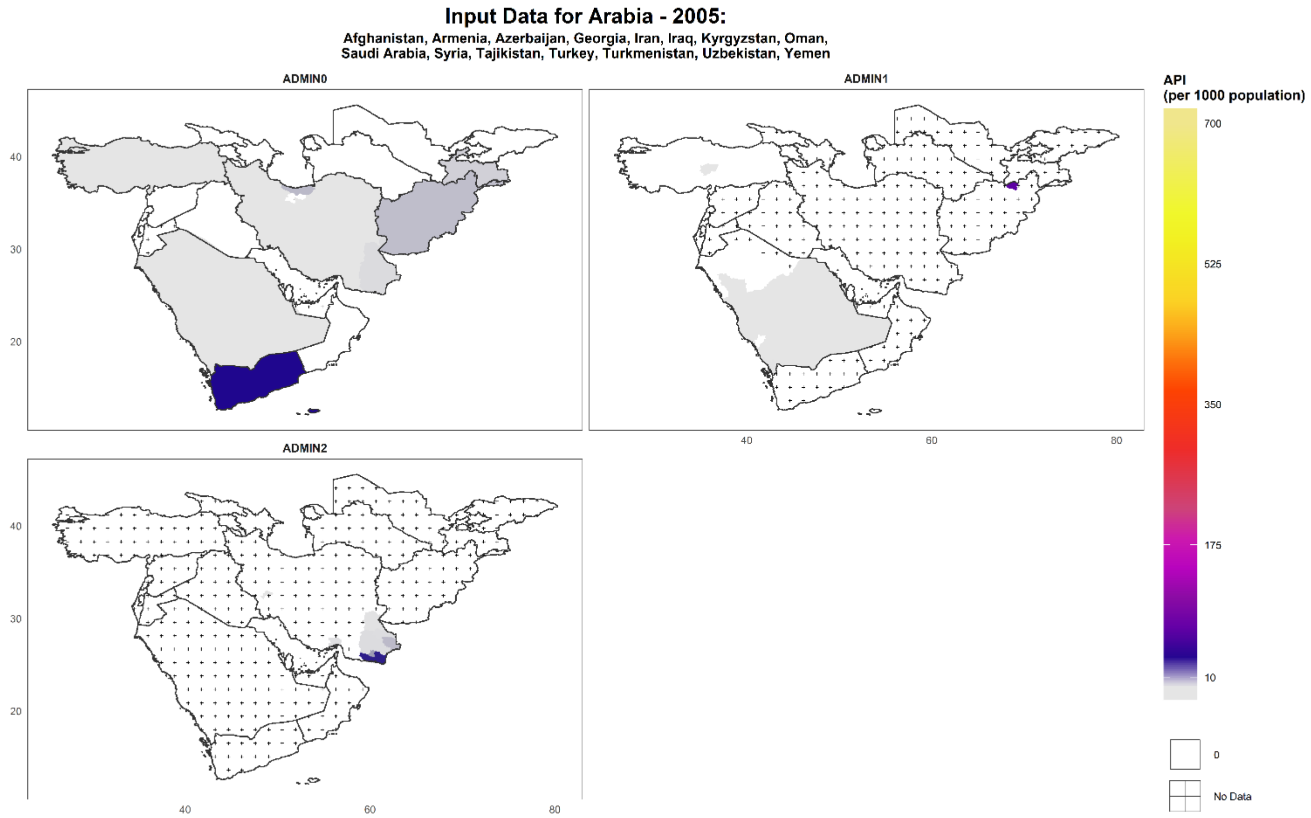

Figure S9: Arabia 2005 input data facetted by admin level.

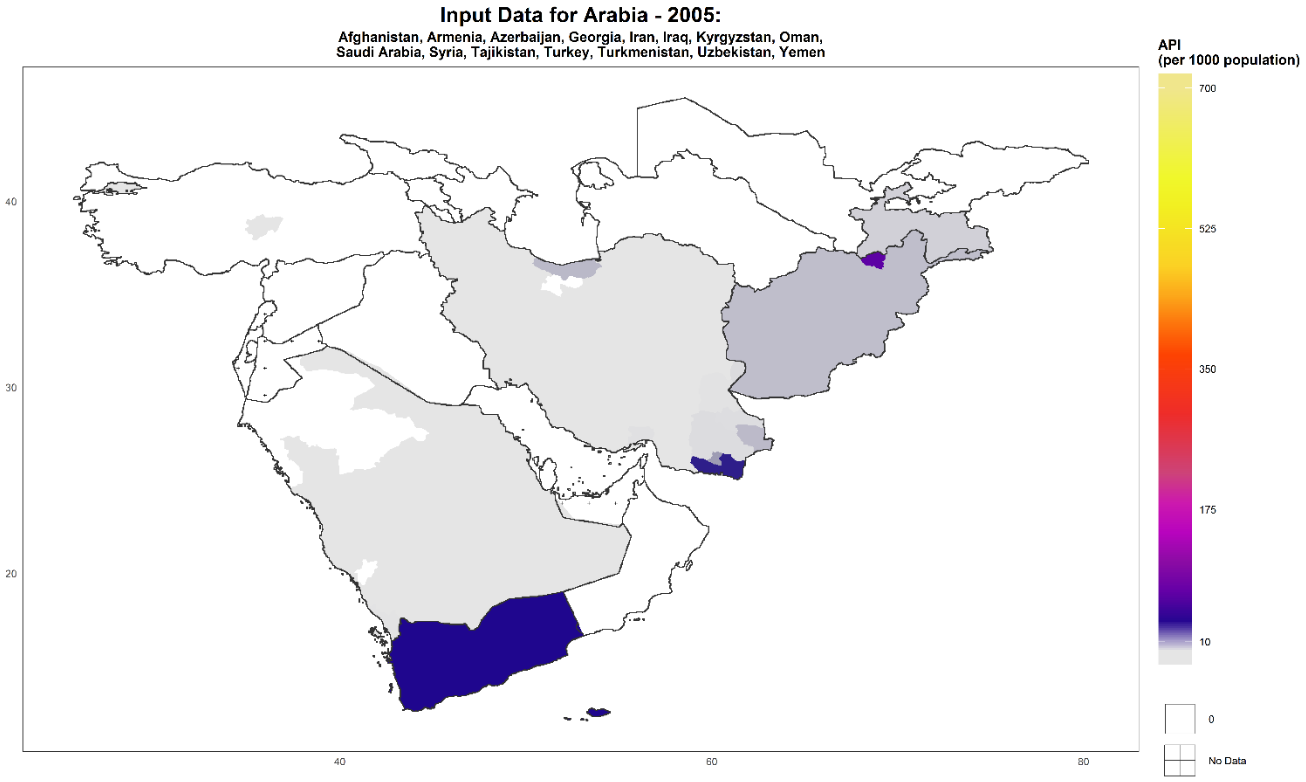

Figure S10: Arabia 2005 input data overplotted such that lower admin level data is on top.

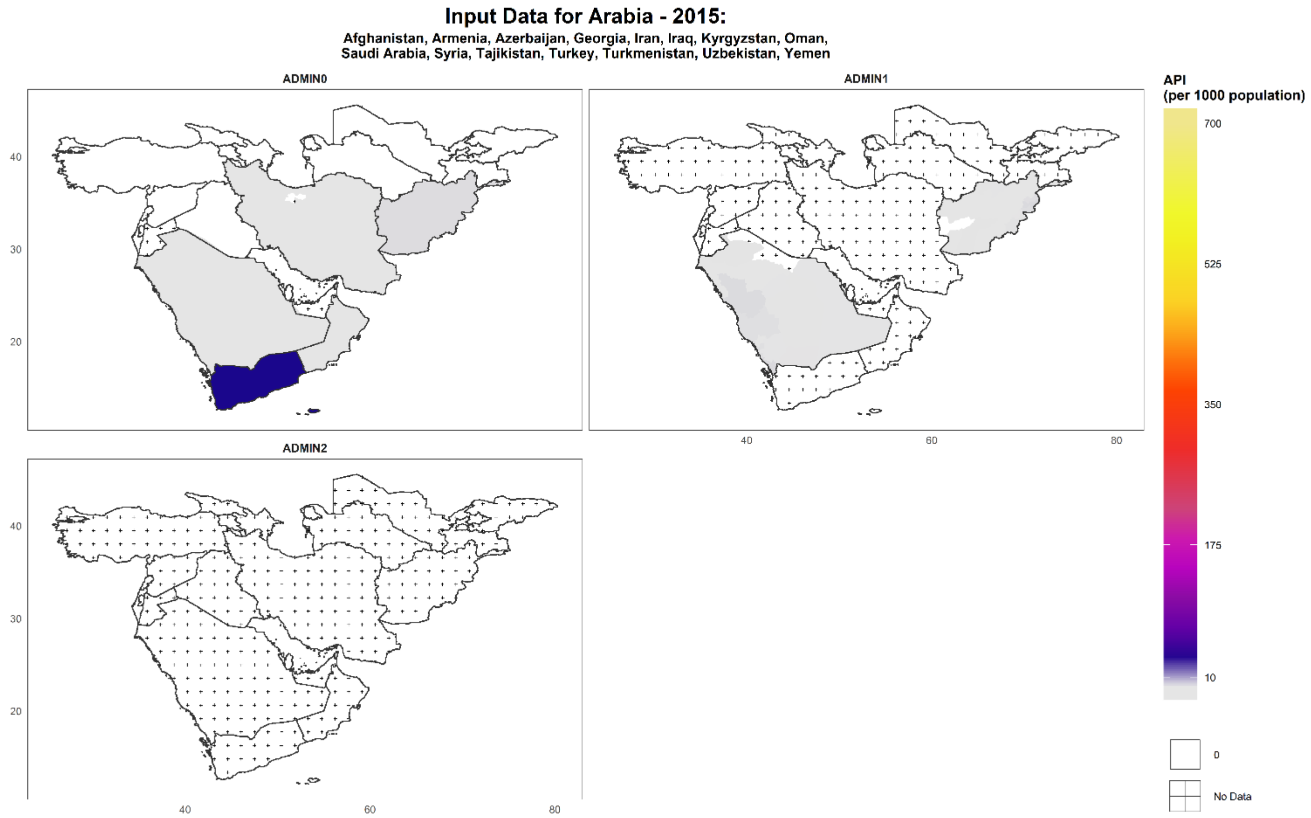

Figure S11: Arabia 2015 input data facetted by admin level.

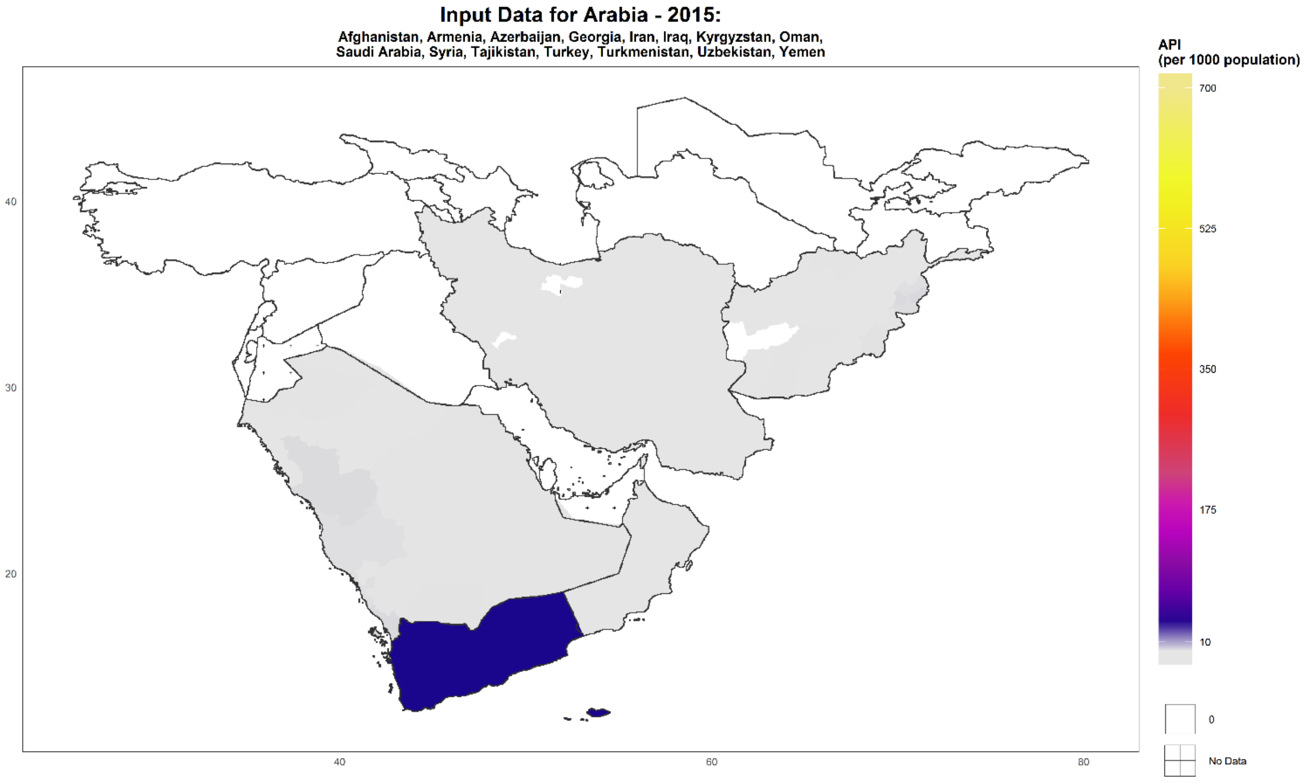

Figure S12: Arabia 2015 input data overplotted such that lower admin level data is on top.

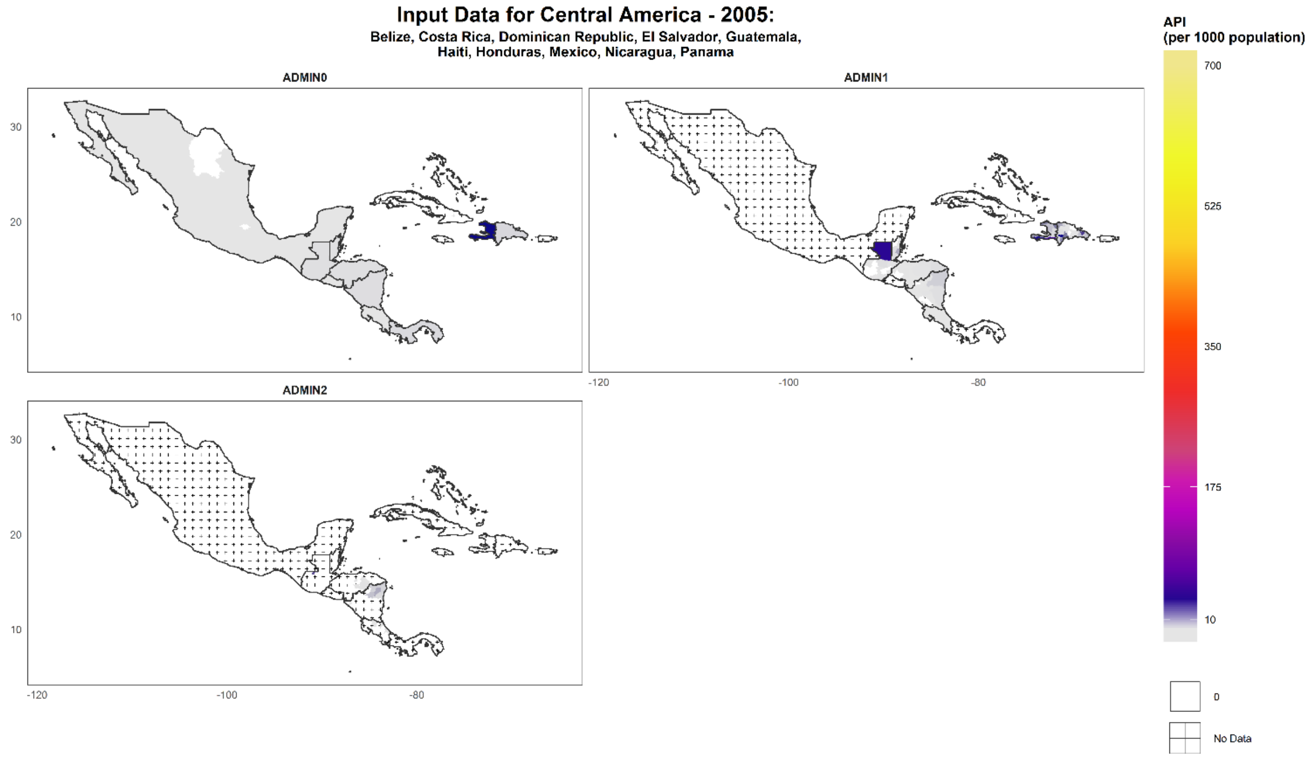

Figure S13: Central America 2005 input data facetted by admin level.

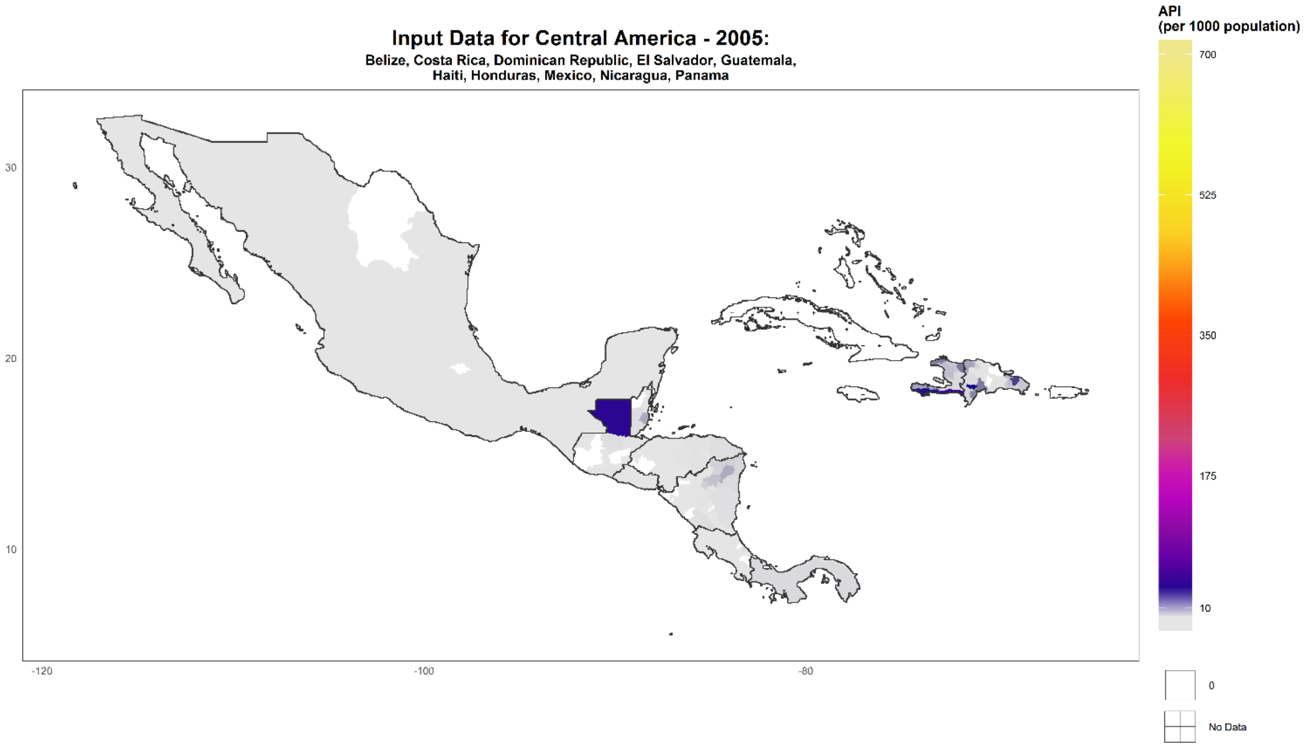

Figure S14: Central America 2005 input data overplotted such that lower admin level data is on top.

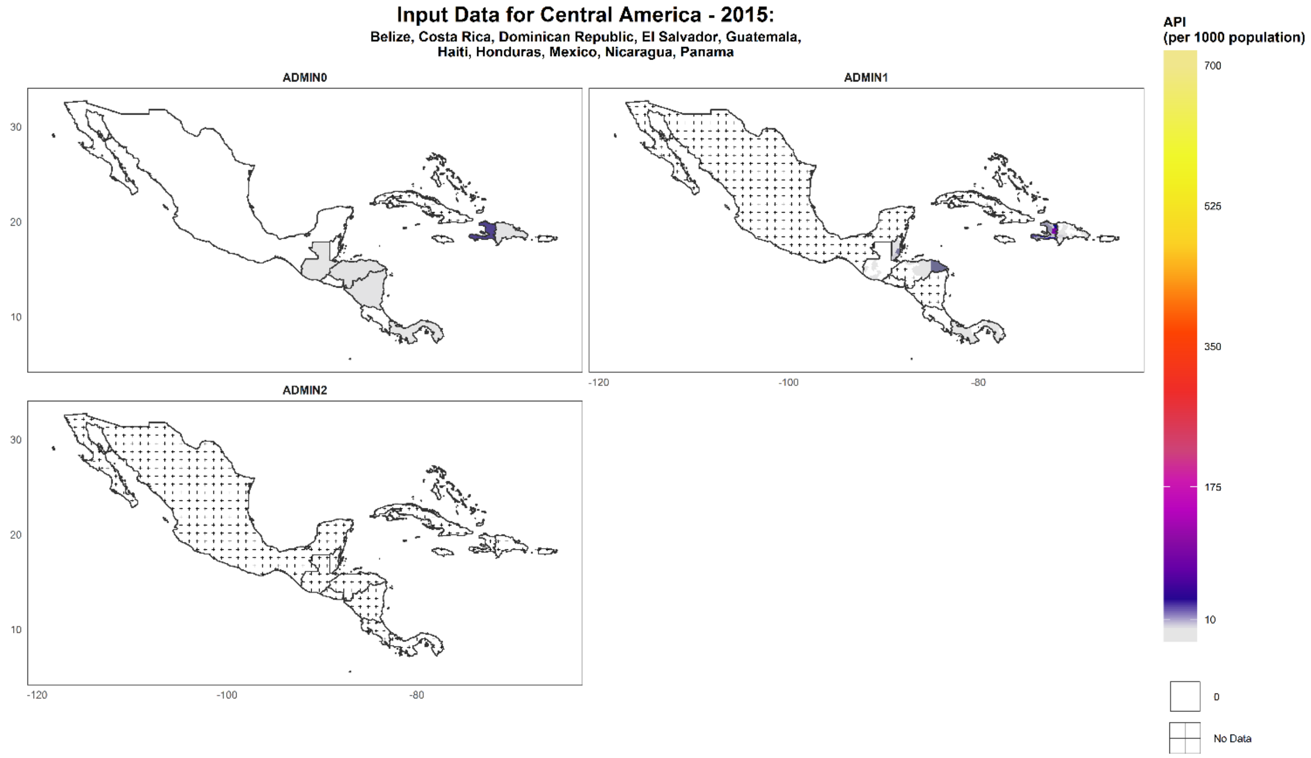

Figure S15: Central America 2015 input data facetted by admin level.

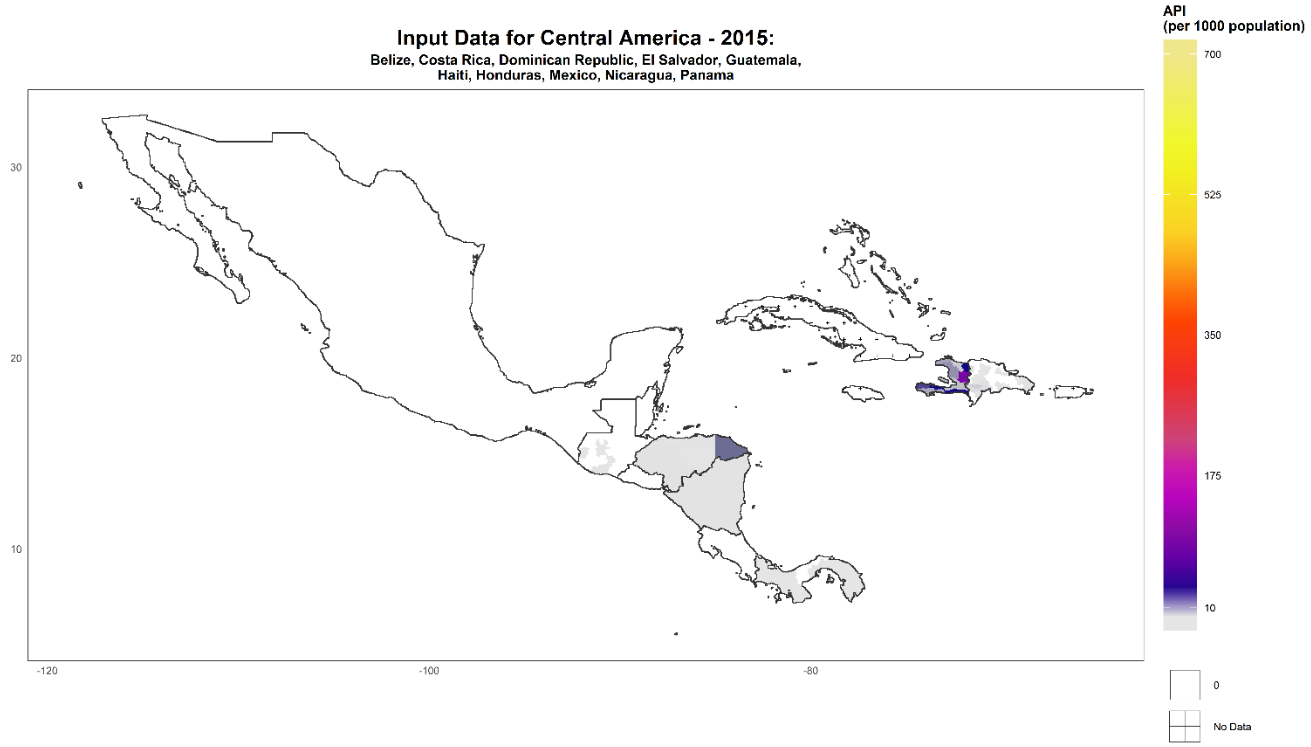

Figure S16: Central America 2015 input data overplotted such that lower admin level data is on top.

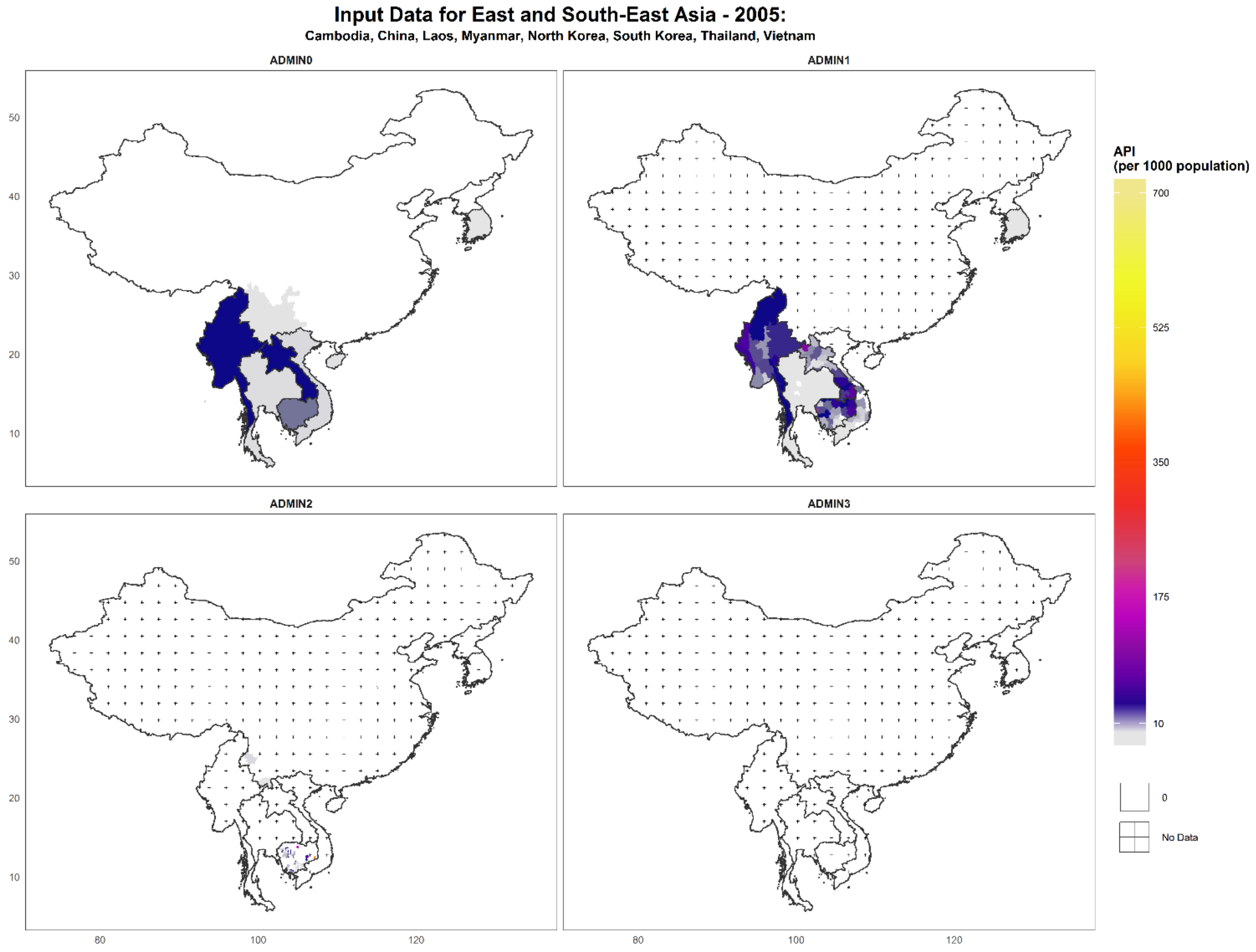

Figure S17: East and South-East Asia 2005 input data facetted by admin level.

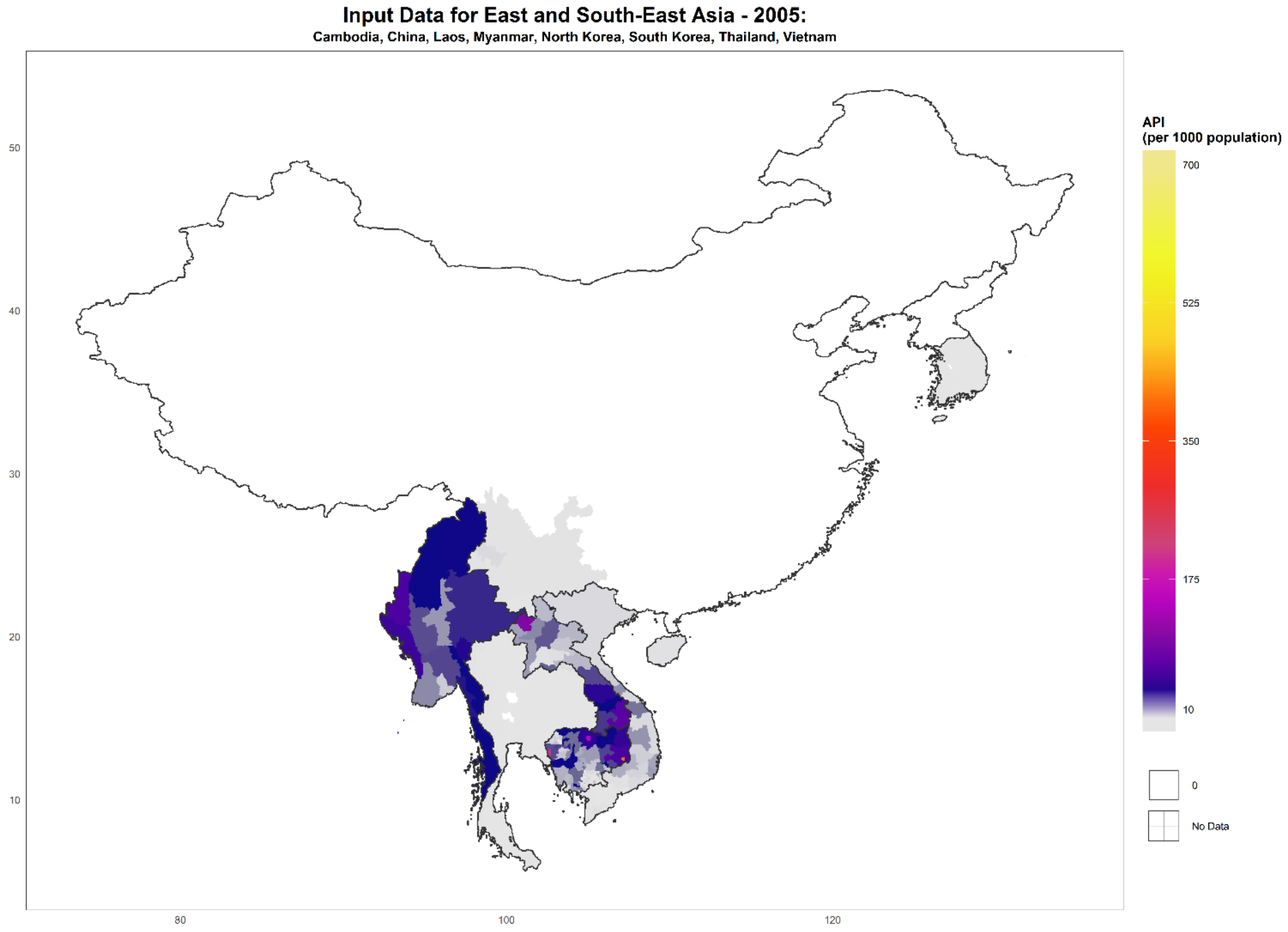

Figure S18: East and South-East Asia 2005 input overplotted such that lower admin level data is on top.

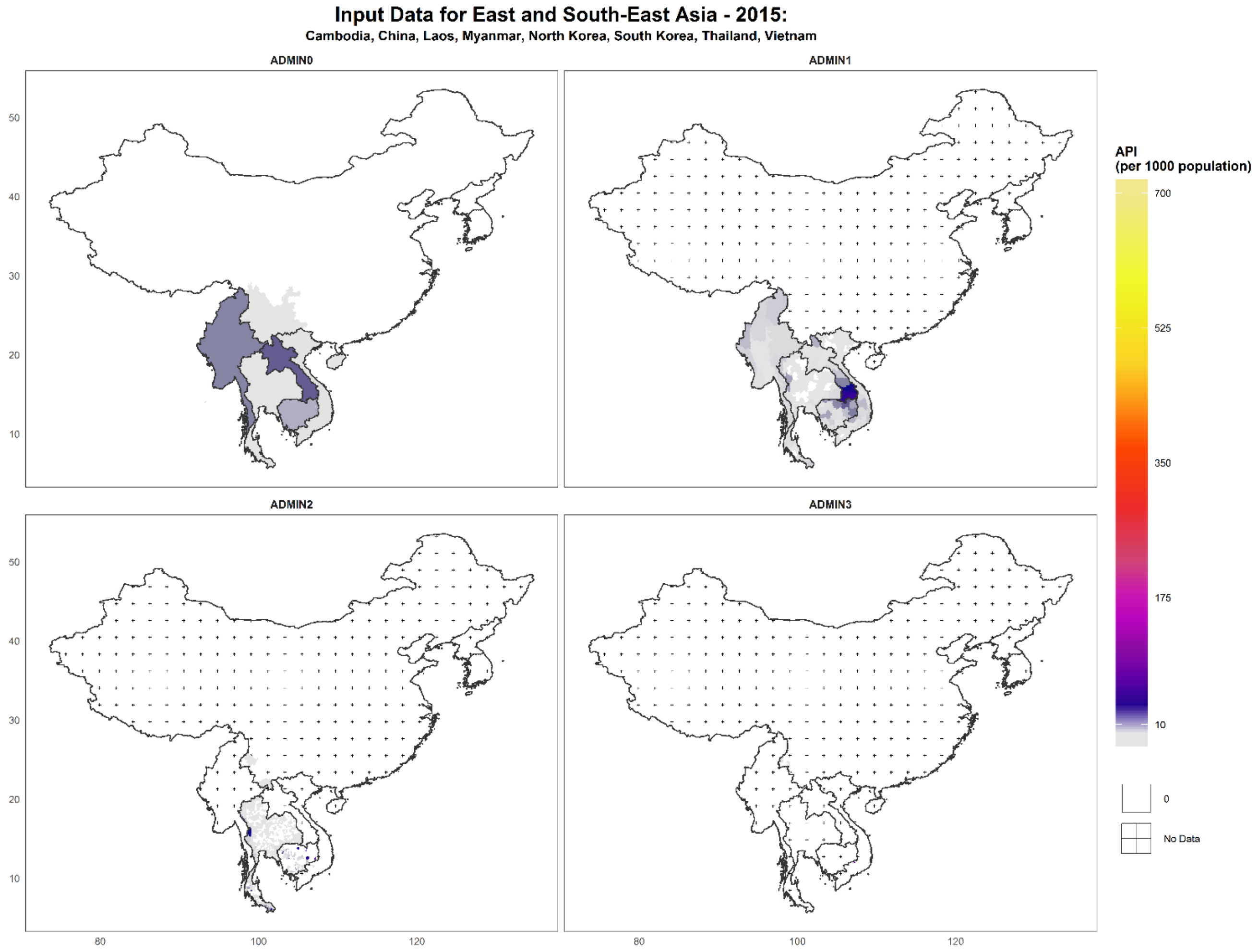

Figure S19: East and South-East Asia 2015 input data facetted by admin level.

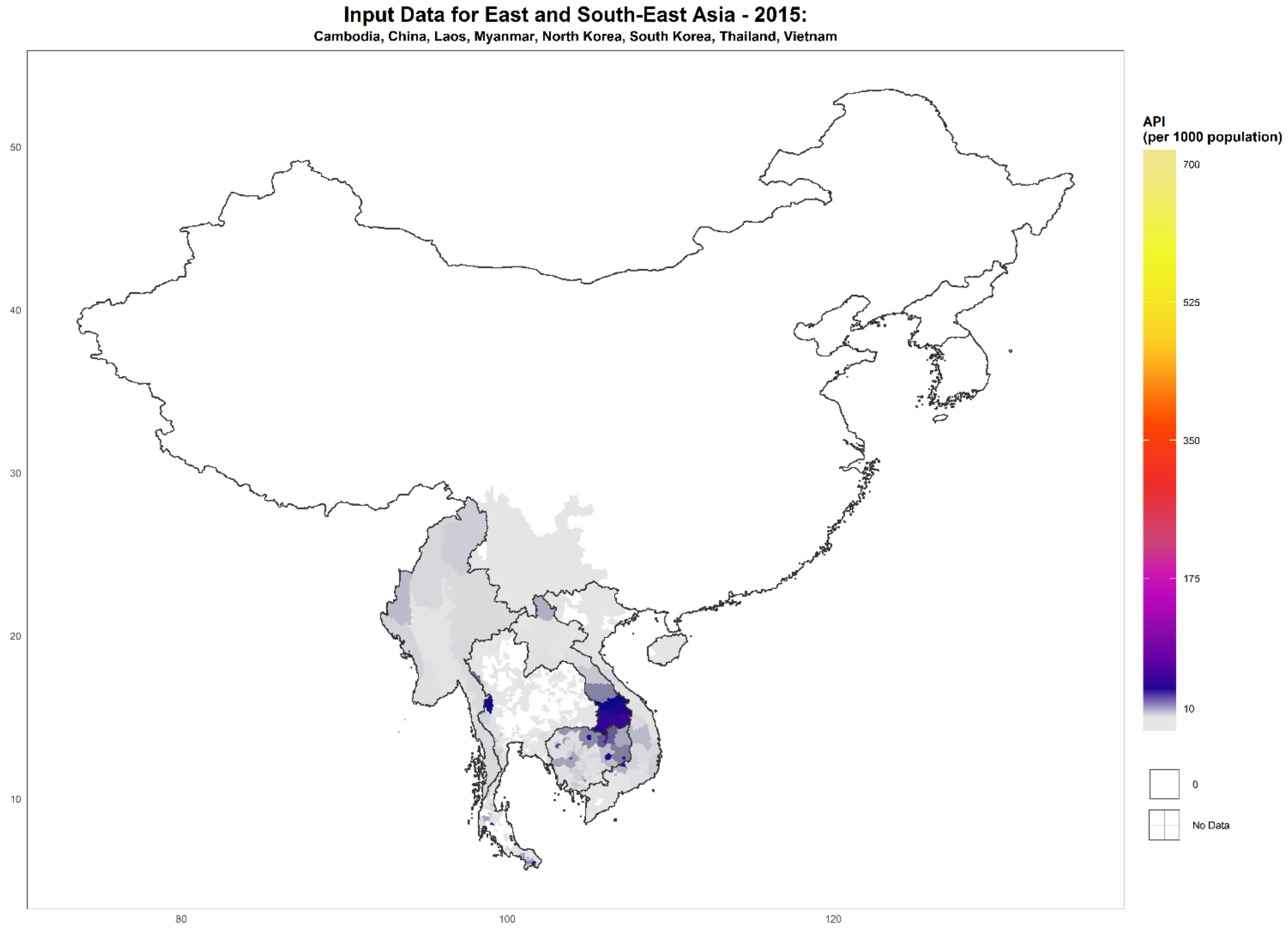

Figure S20: East and South-East Asia 2015 input data overplotted such that lower admin level data is on top.

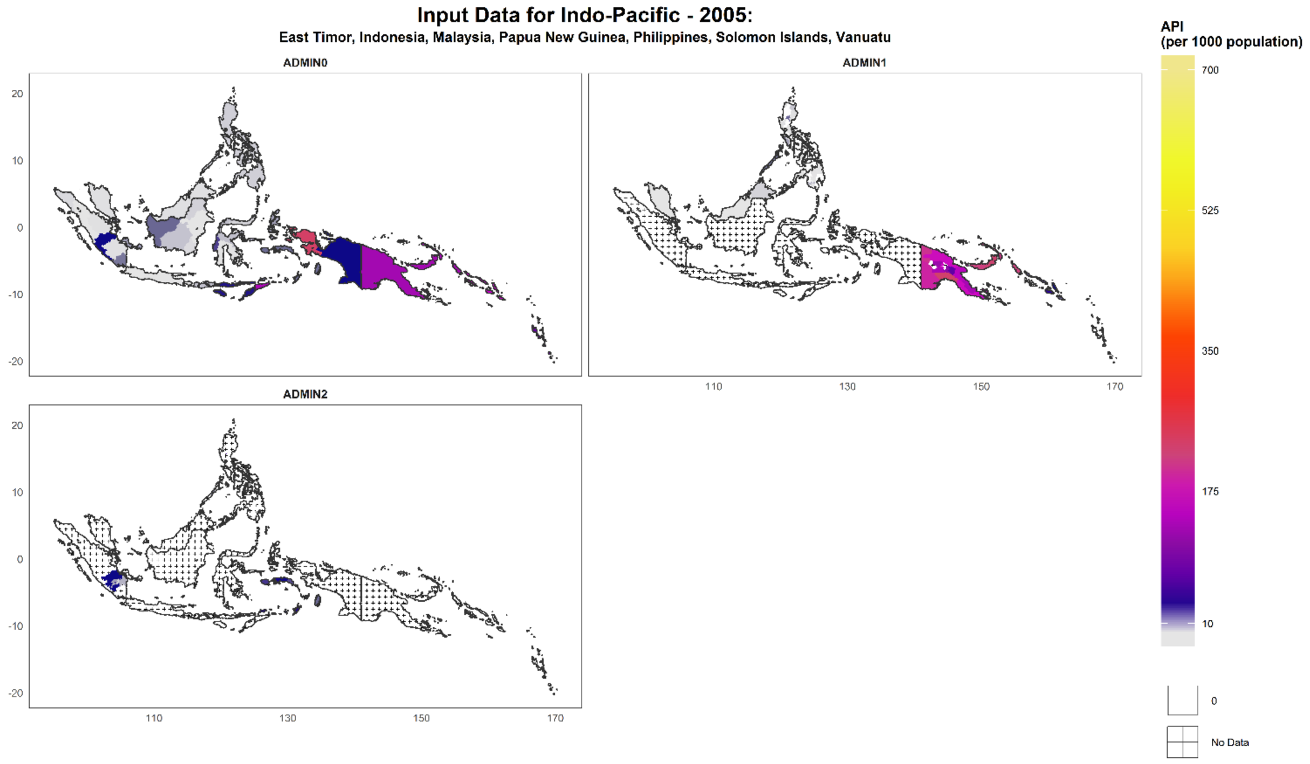

Figure S21: Indo-Pacific 2005 input data facetted by admin level.

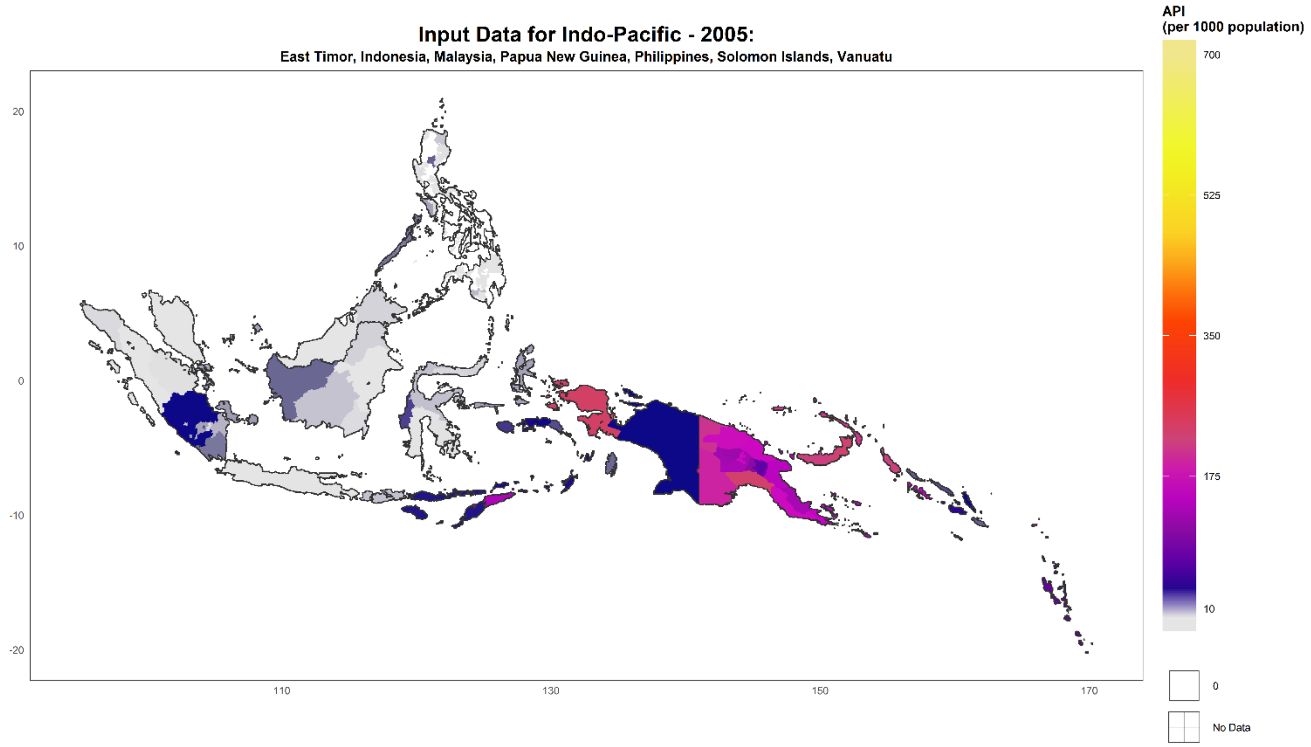

Figure S22: Indo-Pacific 2005 input overplotted such that lower admin level data is on top.

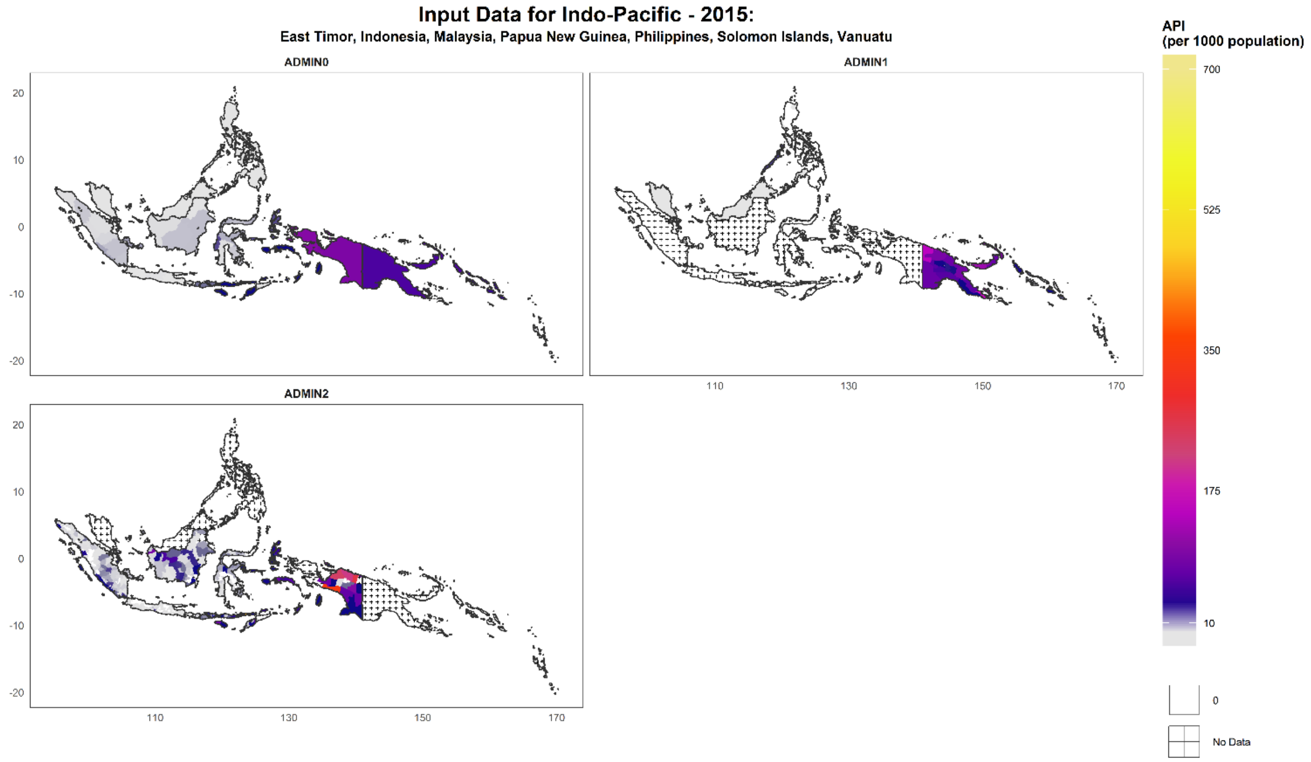

Figure S23: Indo-Pacific 2015 input data facetted by admin level.

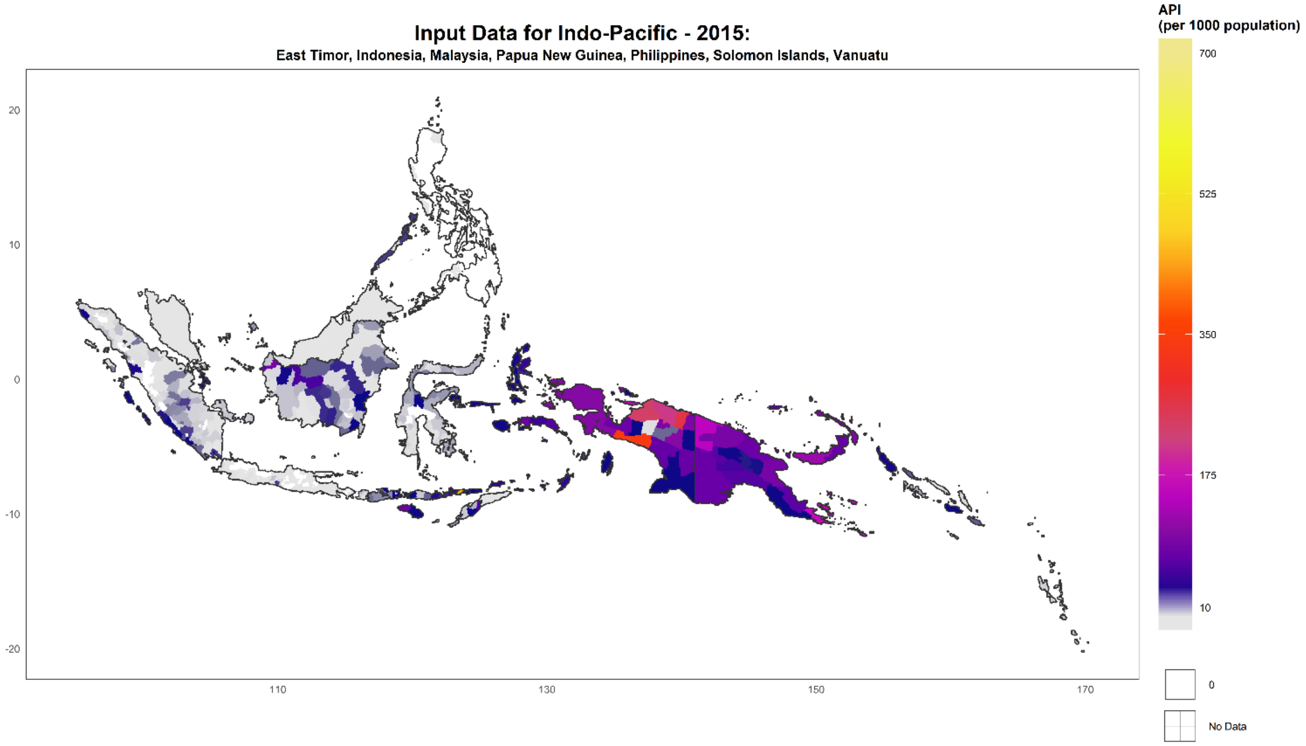

Figure S24: Indo-Pacific 2015 input data overplotted such that lower admin level data is on top.

1.2.6.2.2 Model definition

We define a multi-level, disaggregation regression model inspired by Sturrock *et al.*[42] This model uses polygon-level incidence rate data with pixel-level covariates and a spatial random field. Throughout, we index polygon-level variables with j and pixel level variables with i.
We start by defining the linear predictor which contains an intercept (β_0), covariates ($\boldsymbol{\beta}\mathbf{X}$), a spatial random field ($\mathrm{GP}(s_i)$) and an iid random effect (u_j)

$$\eta_i = \beta_0 + \boldsymbol{\beta}\mathbf{X}_i + \mathrm{GP}(s_i) + u_j.$$

Here, $\boldsymbol{\beta}$ is a vector of M regression slope parameters and $\mathbf{X}_i$ is a vector of M covariate values at pixel i. The grouping for the iid random effect is the ADMIN0 polygon that each polygon is within. The one exception to this is India as dividing the states into rural and urban means polygons no longer fit into a clear hierarchy. Therefore in India there is one group per state and each group will contain two ADMIN0 polygons.

The spatial random effect is a continuous random field. For tractability the random field is implemented as a Gaussian Markov Random Field approximation to the full continuous field using the SPDE approach.[43] We used

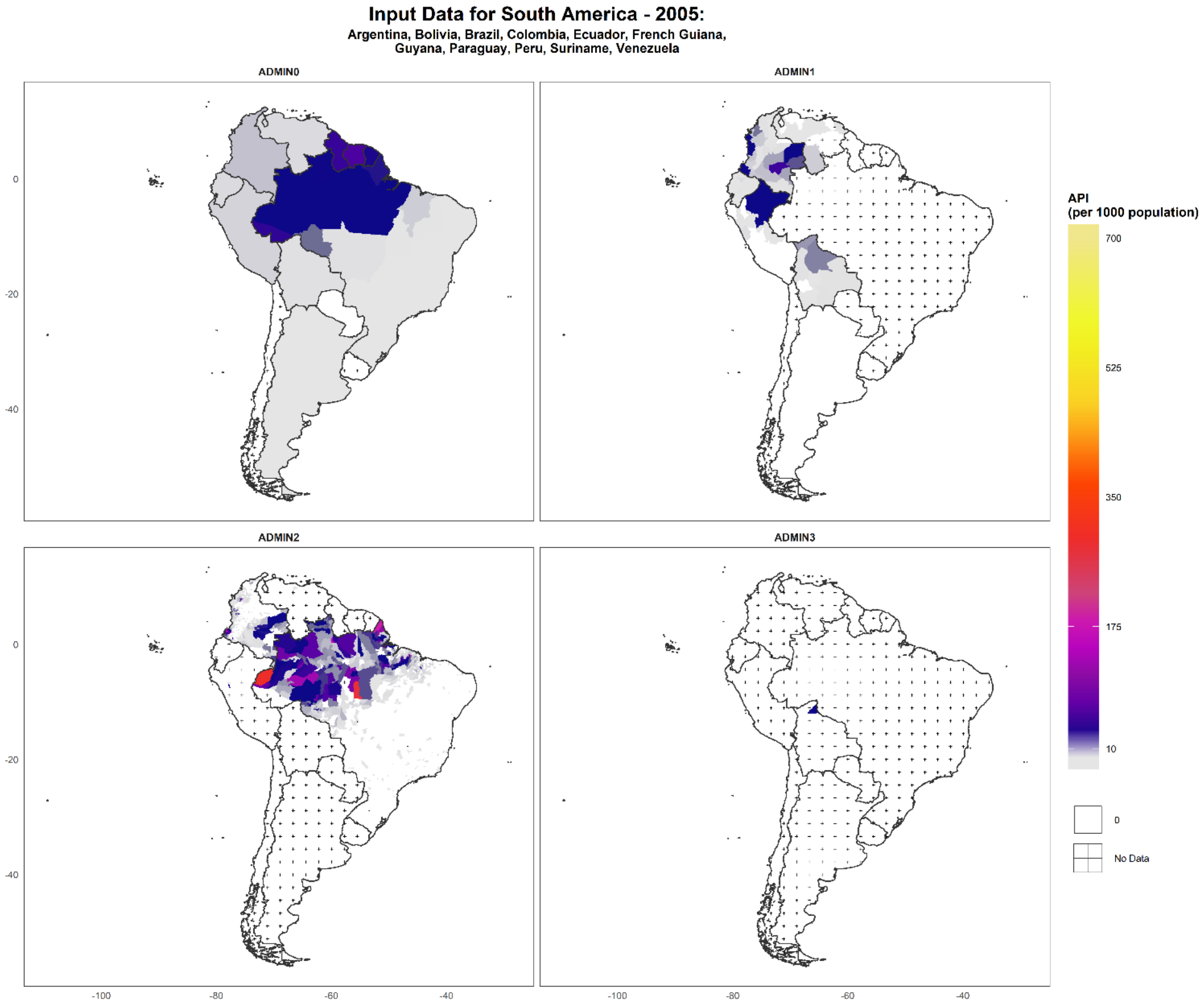

Figure S25: South America 2005 input data facetted by admin level.

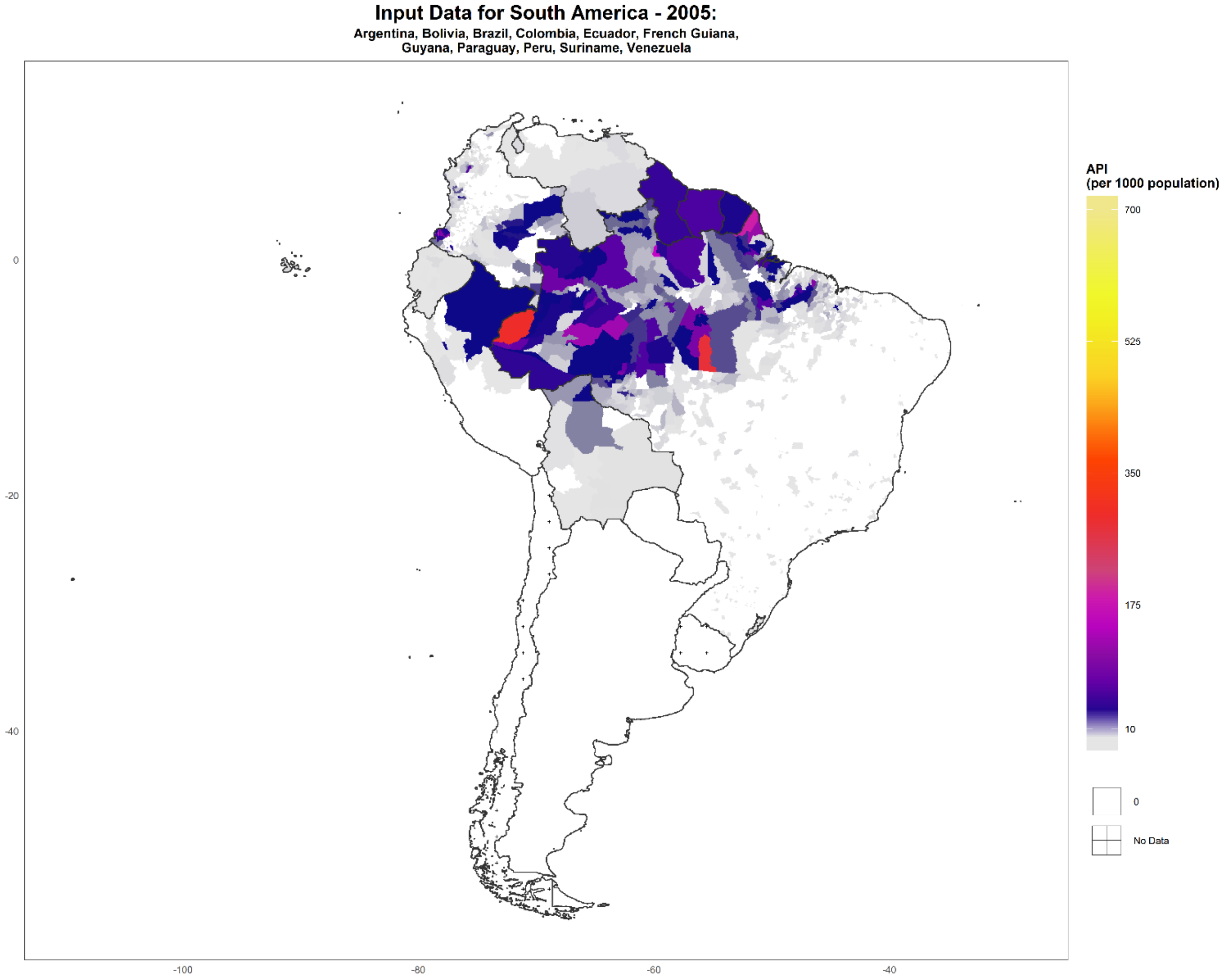

Figure S26: South America 2005 input data overplotted such that lower admin level data is on top.

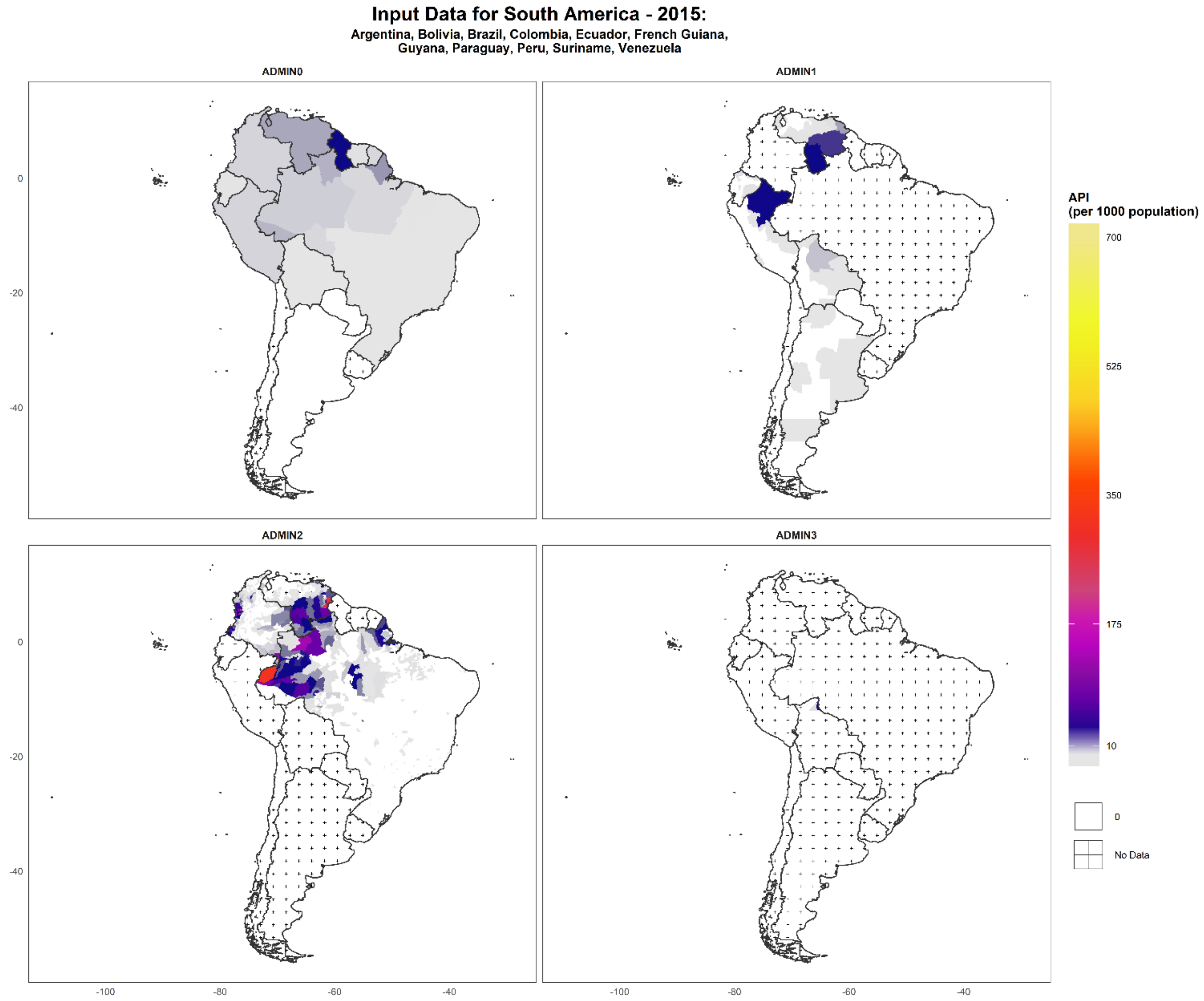

Figure S27: South America 2015 input data facetted by admin level.

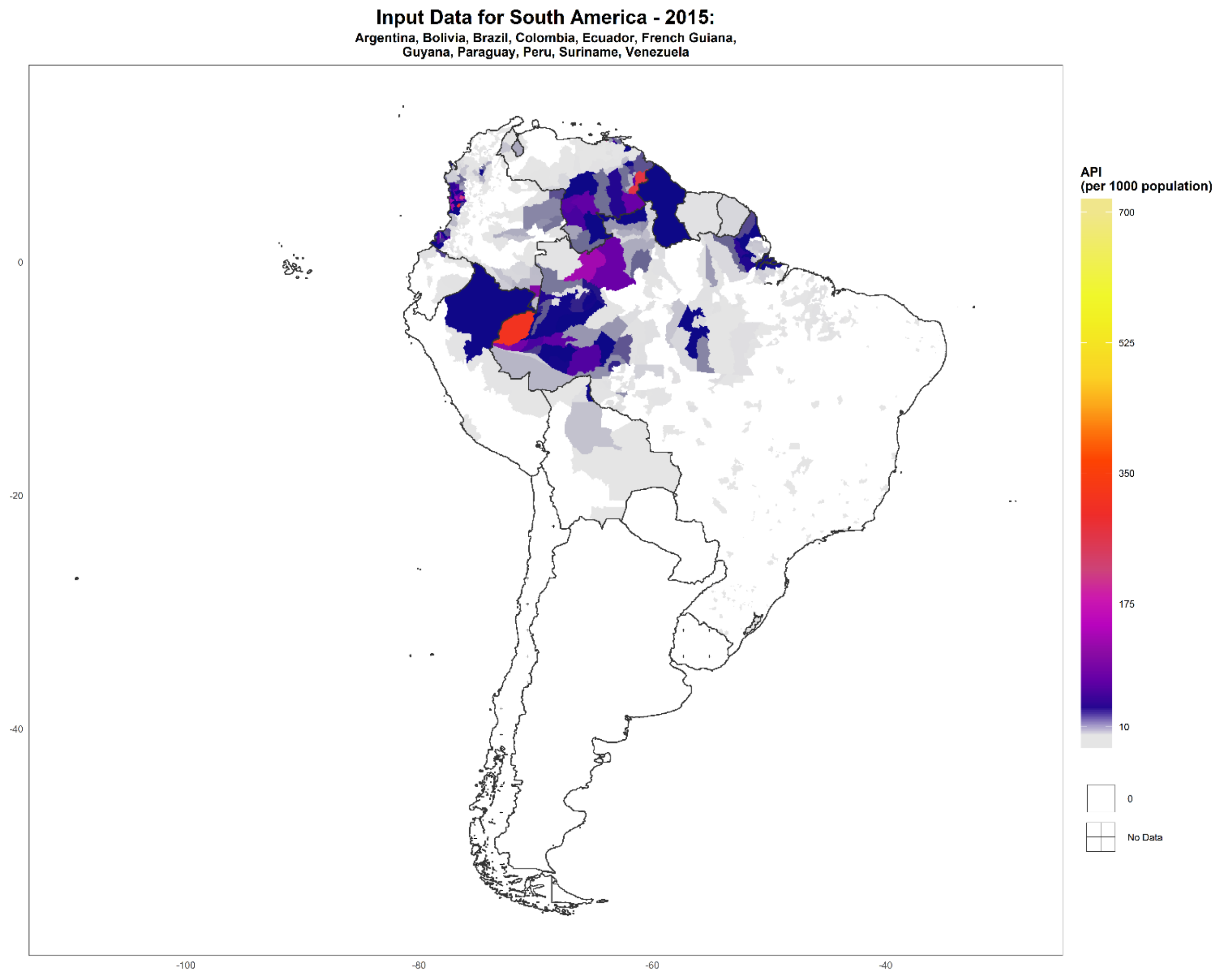

Figure S28: South America 2015 input data overplotted such that lower admin level data is on top.

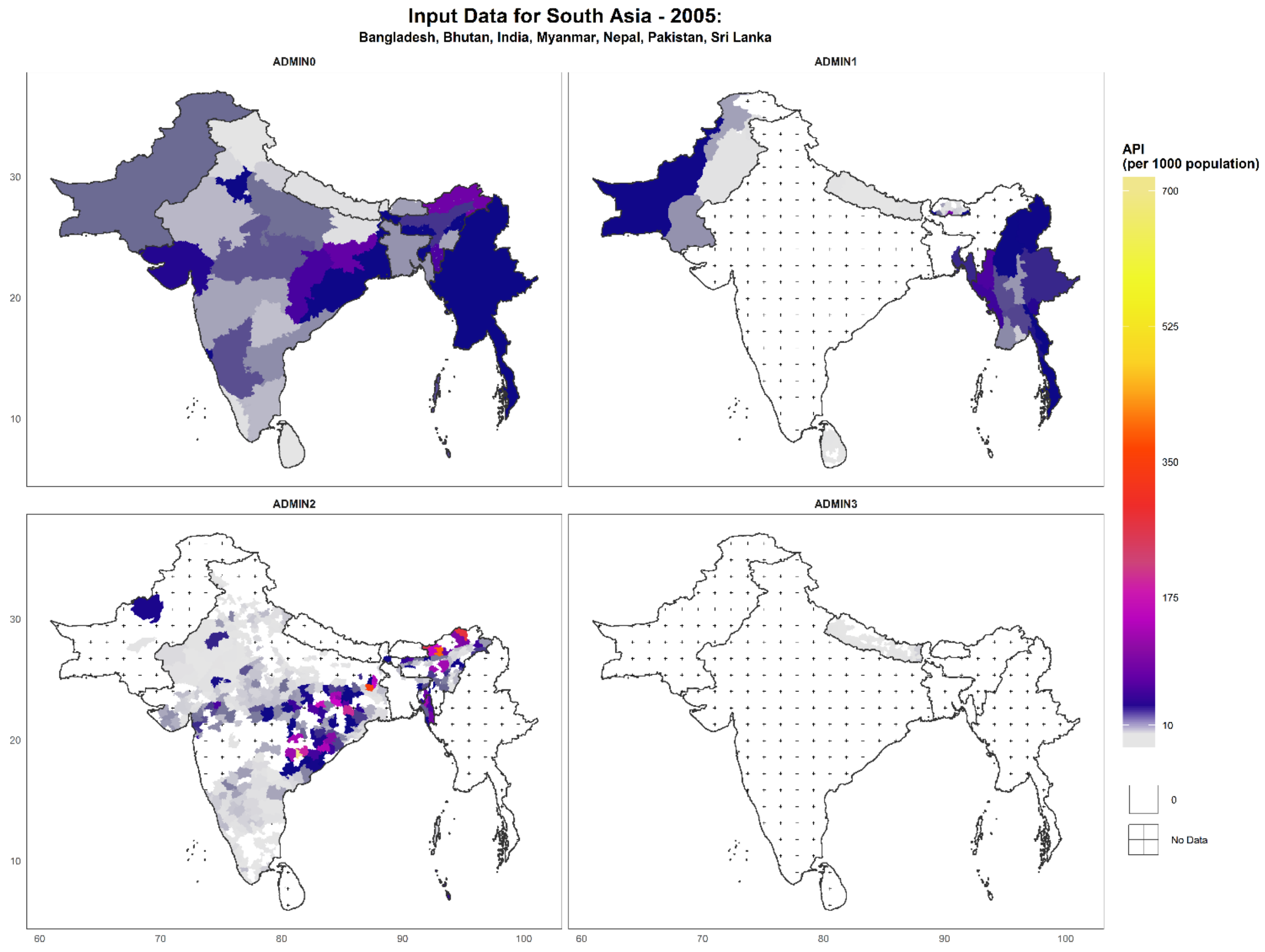

Figure S29: South Asia 2005 input data facetted by admin level.

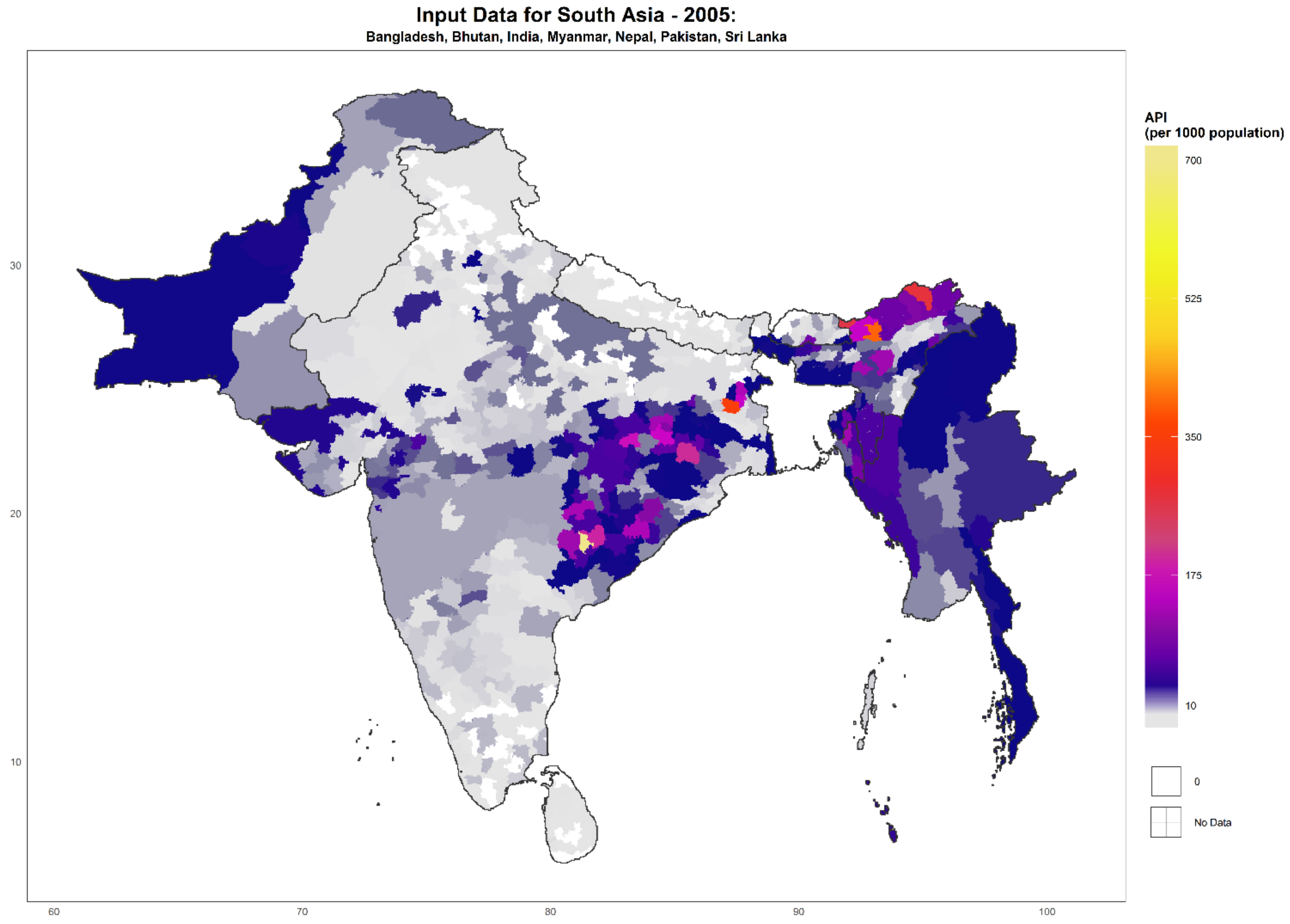

Figure S30: South Asia 2005 input data overplotted such that lower admin level data is on top.

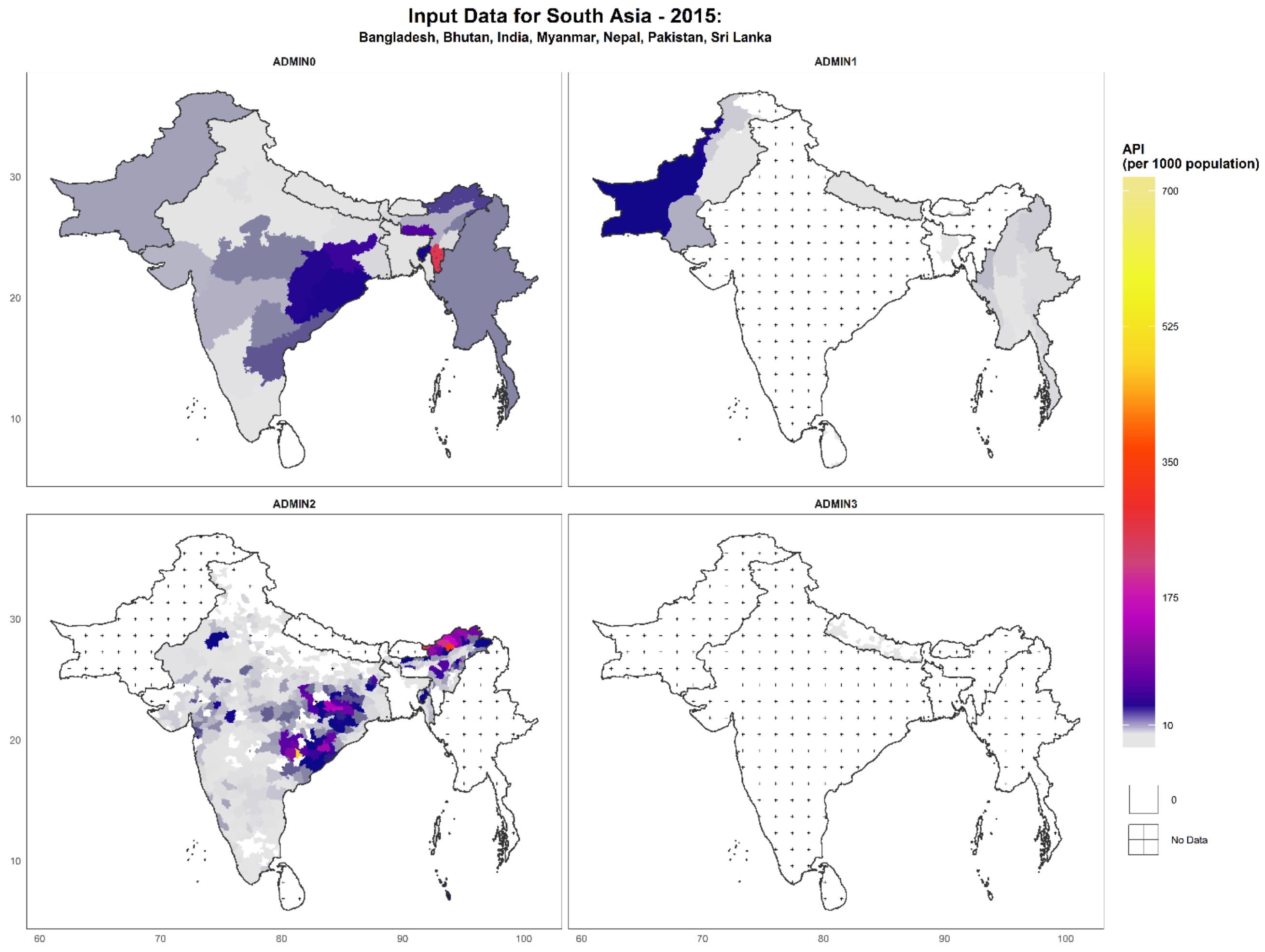

Figure S31: South Asia 2015 input data facetted by admin level.

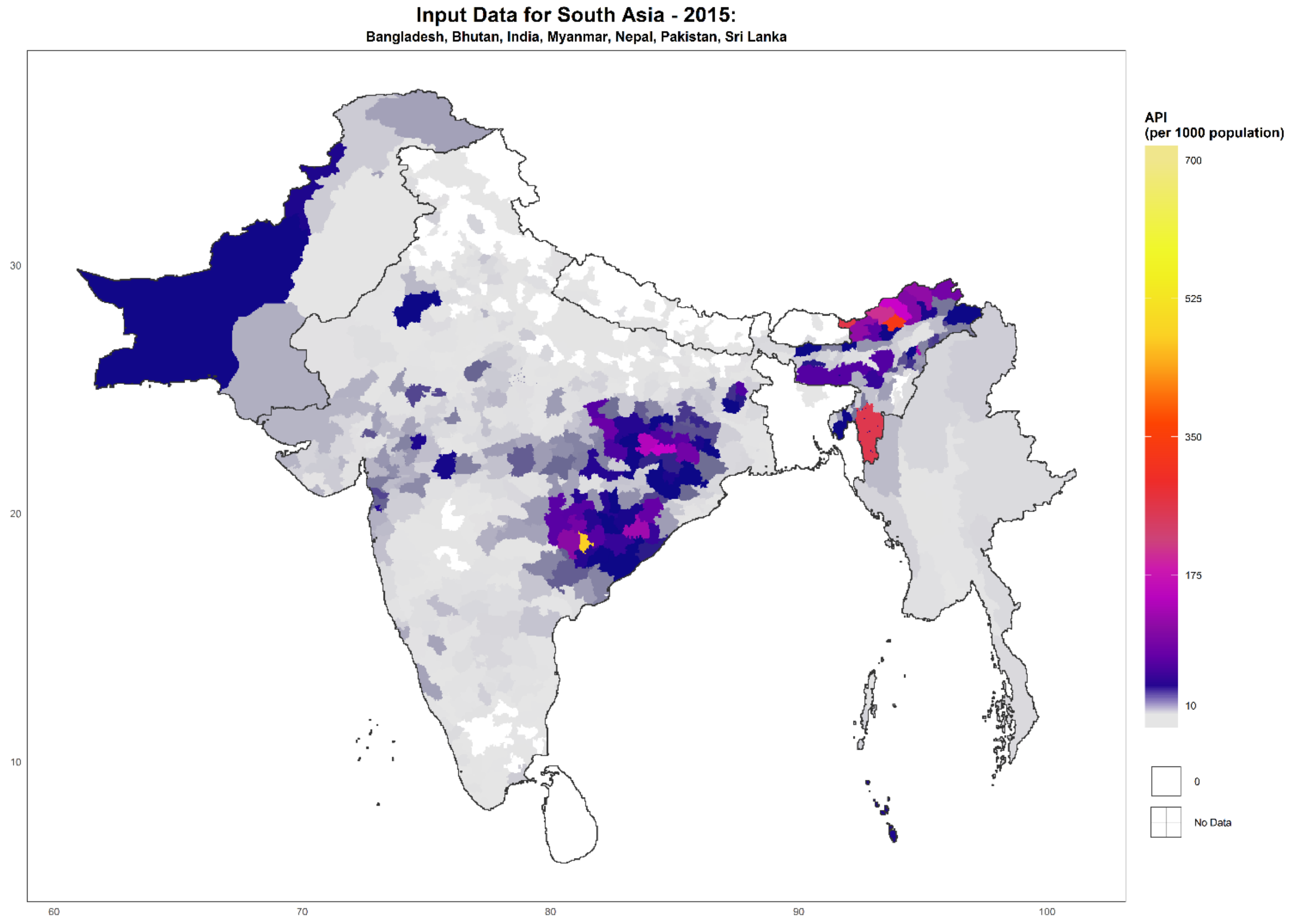

Figure S32: South Asia 2015 input data overplotted such that lower admin level data is on top.

the Matern covariance function (as required by this approximation) and created triangular meshes for each separately modelled region.

We then define the link-function between the linear predictor and I_i. In order to consistently link incidence and prevalence, we choose a link function that passes through prevalence. Furthermore, as this model is only being applied in lower burden areas, we constrain the model to be defined between an incidence of 0 and $I_{max} = 0.612$ as 0.612 is the maximum value given by the prevalence incidence relationship. This corresponds to a maximum prevalence $P_{\max} = 0.616$. This constraint prevents many stability issues caused by the non-monotonic shape of Prev2Inc.

We have pixel-level prevalence P_i given by

$$P_i = P_{\max}\mathrm{logit}(\eta_i)^2$$

where the exponent simply scales the linear predictor to help small incidences to be distinguishable. From here we have

$$I_i = \mathrm{Prev2Inc}(P_i)$$

to transform to the incidence scale. We then define the relationship between polygon-level incidence, I_j, and pixel-level incidence, I_i

$$I_j = \frac{\sum_{i \in j} I_i \times \mathrm{pop}_i}{\sum_{i \in j} \mathrm{pop}_i}.$$

with pop_i being the pixel-level population and the summations going over all pixels in polygon j.

1.2.6.2.3 Likelihood definition

We use a pseudo-likelihood that captures the uncertainty in incidence values. For each incidence value we have an upper bound and lower bound, I_j^U and I_j^L. For ADMIN0 data these bounds are given by the 95% uncertainty intervals from the time-series models. For subnational data the bounds are given by incidence rates calculated using M_{upper} and M_{lower} as described in section “AFI formulae”. We define the likelihood for each incidence value as

$$\log(I_j) \sim \mathrm{Norm}(\mu_j, \sigma_j^2)$$

$$\mu_j = \log\left(\frac{I_j^U + I_j^L}{2}\right)$$

$$\sigma_j^2 = \frac{\log(I_j^U) + \log(I_j^L)}{2 \times 1.96}$$

For data with $I_j^U = I_j^L$ we assign σ_j^2 as the smallest non-zero value of σ_j^2.

1.2.6.2.4 Priors

We set the priors on the fixed effects, β_0 and $\boldsymbol{\beta}$ as

$$\beta_0 \sim \mathrm{Norm}(-4,2)$$

and

$$\beta_{m \in 1:M} \sim \mathrm{Norm}(0,0.5).$$

The weakly informative, zero centred priors on the regression parameters help to regularise the model. This is particularly important due to the large number of covariates being used relative to the number of data points being included in each modelled region. To select this value we considered that one covariate alone should not be able to explain the full range of observed malaria incidence rates. The 95% quantiles of this prior, coupled with approximately normal covariates scaled to have a standard deviation of one, would allow a single covariate to explain a little less than the full range of observed malaria incidence.

The spatial random field has a Gaussian process prior with hyperpriors

$$\log(\kappa) \sim \mathrm{Norm}(-3, 0.3)$$

and

$$\log(\tau) \sim \mathrm{Norm}(0, 1).$$

Finally, the iid random effect has prior

$$u_j \sim \mathrm{Norm}(0, 1).$$

Again, the zero-centred, weakly-informative prior helps to prevent overfitting.

1.2.6.2.5 Weighting by admin level

The polygon data has a hierarchical structure with ADMIN0 polygons and nested, subnational ADMIN1 and ADMIN2 polygons. The data within these levels is very imbalanced and varies from country to country. One country may have an ADMIN0 data value and thousands of subnational data values, while another may only have data for ADMIN0.

To address this imbalance, we weight the data by admin level. We weight ADMIN0 data by 5, ADMIN1 by 1, ADMIN2 by 0·01 and ADMIN3 by 0·005. In the South America region, due to the very large number of ADMIN2 datapoints, we instead weight ADMIN2 and ADMIN3 by 0.001. Given that only around 10 countries are included in each regional analysis, we could not weight ADMIN0 data by 1 and downweight all other data as this would give unreasonable weight to the priors. It was decided that ADMIN1 data are reliable enough to be weighted by 1 with ADMIN0 data upweighted accordingly so that the summed weight of ADMIN1 data for a country is rarely much greater than the weight of the ADMIN0 unit. Similarly, the downweighting values for ADMIN2 and ADMIN3 units are determined so that the summed weight of these data rarely outweigh the higher-level, more reliable, data they are below in the hierarchy.

1.2.6.2.6 Computational model fitting

To find the maximum *a posteriori* estimate for the model, we optimise a vector θ of all parameters and hyperparameters such that we find the values that minimise $-\log(p(I|\theta)p(\theta))$. As this is proportional to the true posterior, the parameter values that minimise this expression are also the mode of the posterior.

The model was defined with the R package Template Model Builder (TMB)[35] and optimised in R.

1.2.6.2.7 Temporal Interpolation

To allow the malaria surface to change through time we fit two models per region (2005 and 2015). After fitting, both these models are predicted globally and malaria surfaces for other year are calculated by linearly interpolating between them. However it is only the underlying surface that it interpolated linearly as the results from the time-series models are raked over these surfaces.

For the covariates other than precipitation, night-time lights, elevation, accessibility and PET, the appropriate year data are used for both model fitting and prediction.

We then linearly interpolate between the two models. For an incidence value at pixel i and year t we have

$$I_{i,t} = w_{2015} I_{i,2015} + (1 - w_{2015}) I_{i,2005}$$

where w_{2015} is the 2015 weight and is given by

$$w_{2015} = \begin{cases} 0, & \text{if } t \le 2005 \\ 1, & \text{if } t \ge 2015 \\ \dfrac{t - 2005}{2015 - 2005}, & \text{if } 2005 < t < 2015 \end{cases}$$

1.2.6.2.8 Masking and population

After the models have been predicted and interpolated, such that we have a full space-time cube of incidence, we use a mask (see section "Post-hoc masking") to ensure that areas known to be malaria free are predicted as such. This is particularly required because the model cannot predict a true zero. For all region-year pair in the post-hoc mask table, we set all incidence and prevalence pixels in the appropriate polygons to zero.

Pixels with zero population-at-risk are also set to incidence and prevalence of zero as these metrics are undefined with a denominator of zero.

1.2.6.2.9 Raking

In order that our final results exactly match the results given in the Global Burden of Disease 2017[40] we rake the published over the final predicted raster so that for a given polygon j, the predicted cases, $\sum_{i \in j} I_i \times \text{pop}_i$, equals the data value μ_j. However, given that we wish to keep incidence and prevalence consistently linked, this raking must be applied to both of these values together. Furthermore, the raking must obey $P_i \in [0, P_{\text{max}}]$. We increase or decrease the P_i values by a nonlinear factor governed by ϕ so that $\sum_{i \in j} I_i \times \text{pop}_i = \mu_j$.

If $\sum_{i \in j} I_i \times \text{pop}_i > \mu_j$ we simply chose ϕ such that

$$\sum_{i \in j} \text{pop}_i \times \text{Prev2Inc}\left(e^{-\phi} P_i\right) = \mu_j.$$

However, if $\sum_{i \in j} I_i \times \text{pop}_i < \mu_j$ we have to make sure that P_i never goes above P_{max}. Therefore we define

$$f(P_i) = -log\left(\frac{2}{(P_i/P_{\text{max}})^{\alpha} + 1} - 1\right)$$

and its inverse

$$f^{-1}(z_i) = P_{\text{max}}\left(\frac{2}{1+\exp(-z_i)} - 1\right)^{\frac{1}{\alpha}}.$$

Here α is a parameter that governs how fast small prevalence values increase relative to large prevalence values. This parameter is set to 0.00001. This value was chosen as it avoided creating very large incidence values too readily.

Again we chose ϕ such that

$$\sum_{i \in j} \text{pop}_i \times \text{Prev2Inc}\left(f^{-1}(\phi f(P_i))\right) = \mu_j.$$

Therefore, for a given ADMIN0 polygon, we increase prevalence by ϕ in the transformed space between 0 and infinity. After the inverse transformation, all values of P_i are therefore still within $[0, P_{\text{max}}]$.

1.2.6.2.10 Bootstrap uncertainty

After finding the maximum *a posteriori* estimate of the posterior we need a method to draw 100 independent samples from the posterior. However, the Laplace approximations calculated by Template Model Builder failed to find estimates for a multivariate normal approximation to the posterior. We also attempted to use Hamiltonion Markov Chains (HMC) to draw samples from the posterior, but the chains mixed very poorly, and given the slow evaluation of $-\log(p(I|\theta)p(\theta))$, the expected runtime to get 100 independent samples was prohibitively high. Similarly, attempts at importance sampling and relative importance sample failed.

Instead we used bootstrapping to obtain 100 samples that characterise the uncertainty in the model. In designing the bootstrap resampling scheme, care was taken to account for both the fact that the ADMIN0 data raked over predicted maps come from a separate time-series model and the hierarchical nature of the polygon data.

For each bootstrap resample, the following scheme was used:

1. For each ADMIN0 unit, sample the posterior of the time-series models.
2. Select polygons with probability 0·05.
3. Remove all data that are descendants of selected polygons (i.e. admin units below the selected polygons).
4. Remove the selected polygons themselves unless the polygon is ADMIN0.
5. Fit the model to the remaining data and rake the surface back to the resampled ADMIN0 values.

Therefore, for each bootstrap, there is always complete ADMIN0 data. This is required as the model is raked to these values in each case. Given that the bootstrap is characterising the uncertainty in the spatial pattern of incidence within ADMIN0 units, this is reasonable.

This sampling scheme also means that the amount of missing data per bootstrap iteration is highly variable: some ADMIN0 units have thousands of descendant polygons while some have none. Furthermore, as each polygon is selected independently, different bootstrap iterations will have different numbers of ADMIN0 polygons that have all their descendants removed. In fact there is no guarantee that any ADMIN0 polygons are selected.

We also note that we did not run 100 separate bootstraps of the machine learning prevalence models. Given their role as fixed covariates, similar to the modelled and gap filled environmental covariates, we deemed this unnecessary.

1.2.6.2.11 Age splitting

Finally, incidence and *Pf* PR estimates were age-split into three bins (0–5; 5–14; >15) using previously published models.[22]

1.3 Results

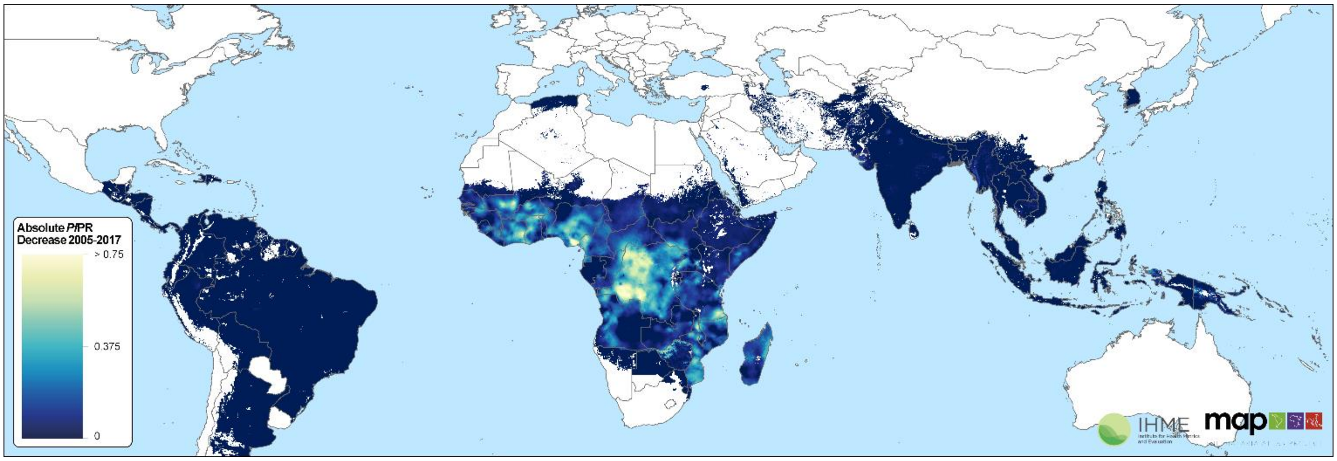

Figure S33: Absolute *Pf*PR decrease 2005-2017.

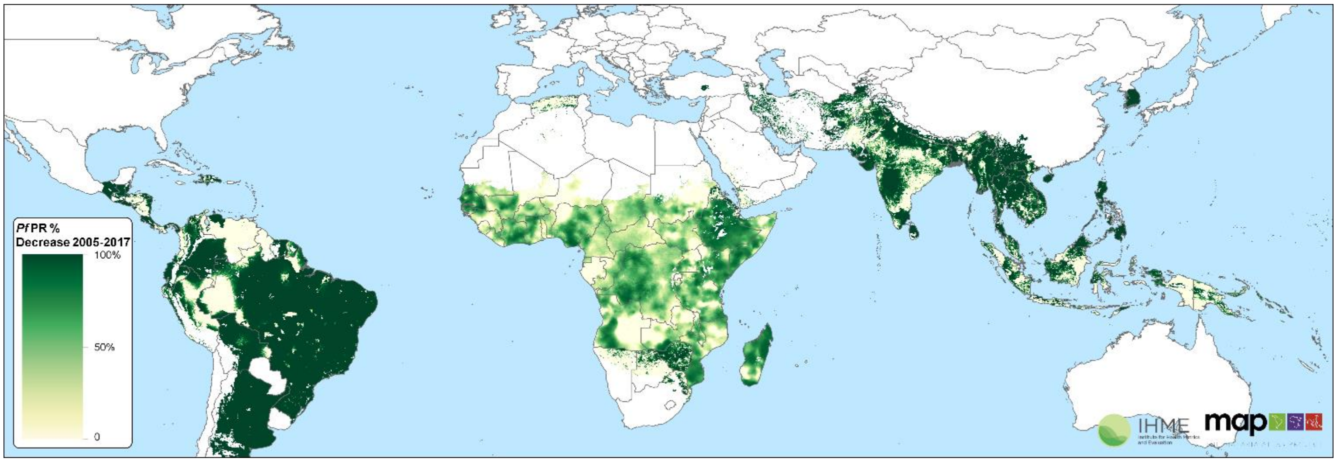

Figure S34: Relative *Pf*PR decrease 2005-2017.

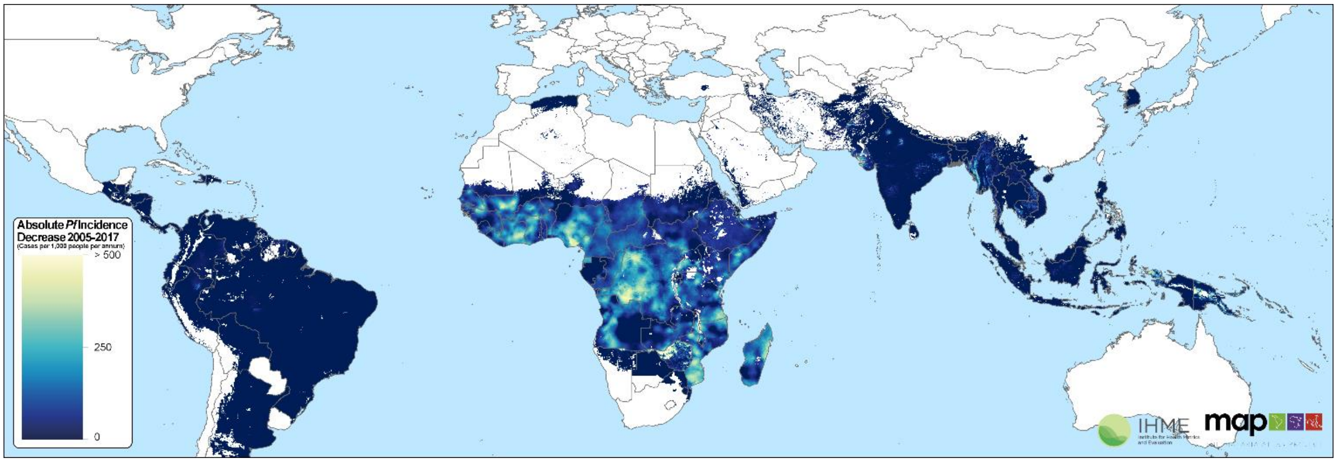

Figure S35: Absolute incidence decrease 2005-2017.

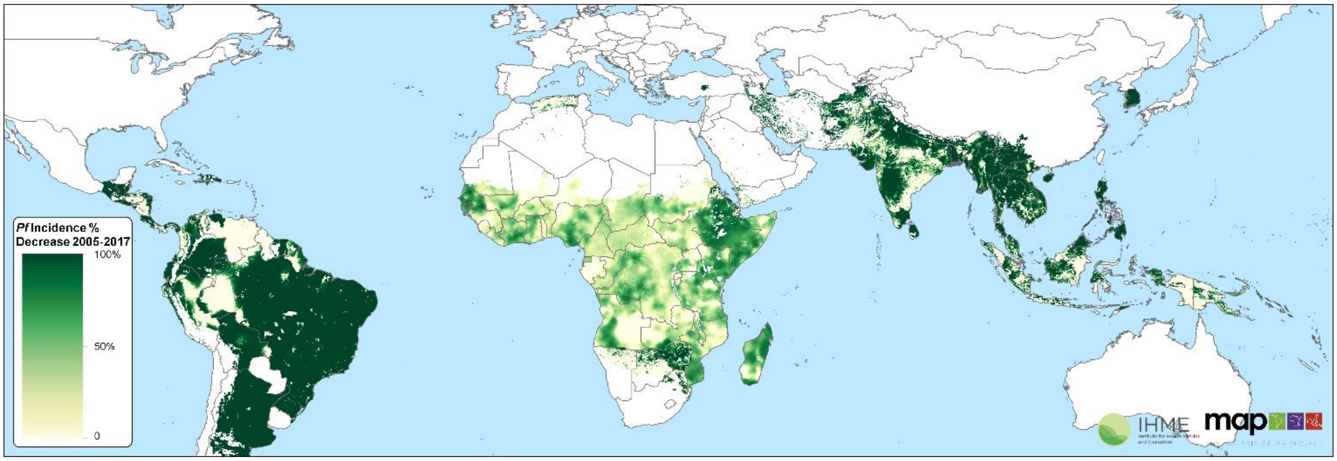

Figure S36: Relative incidence decrease 2005-2017.

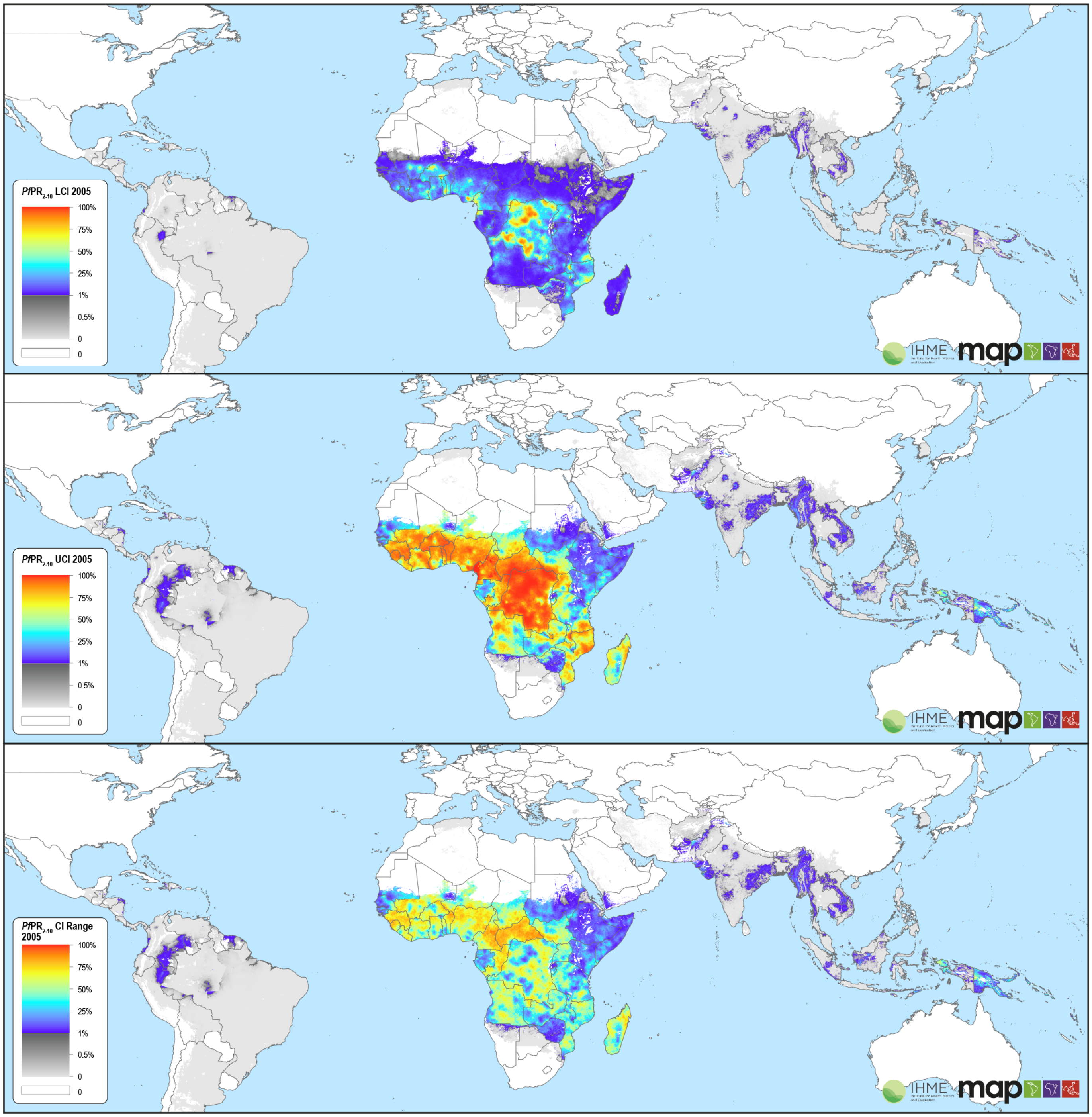

Figure S37: Lower and upper credible intervals (CIs) as well as the range for *Pf*PR2-10 in 2005.

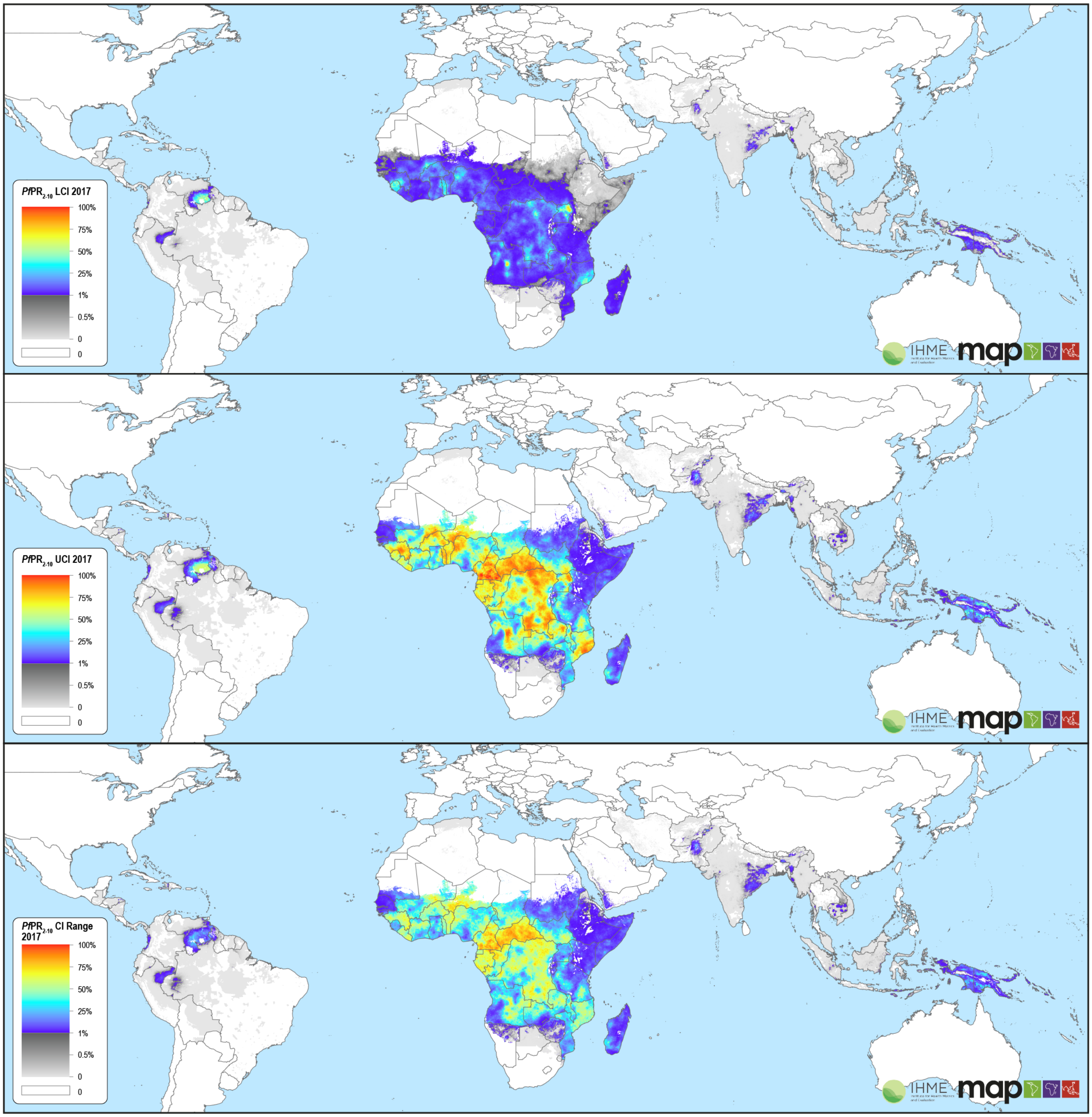

Figure S38: Lower and upper credible intervals (CIs) as well as the range for *Pf*PR2-10 in 2017.

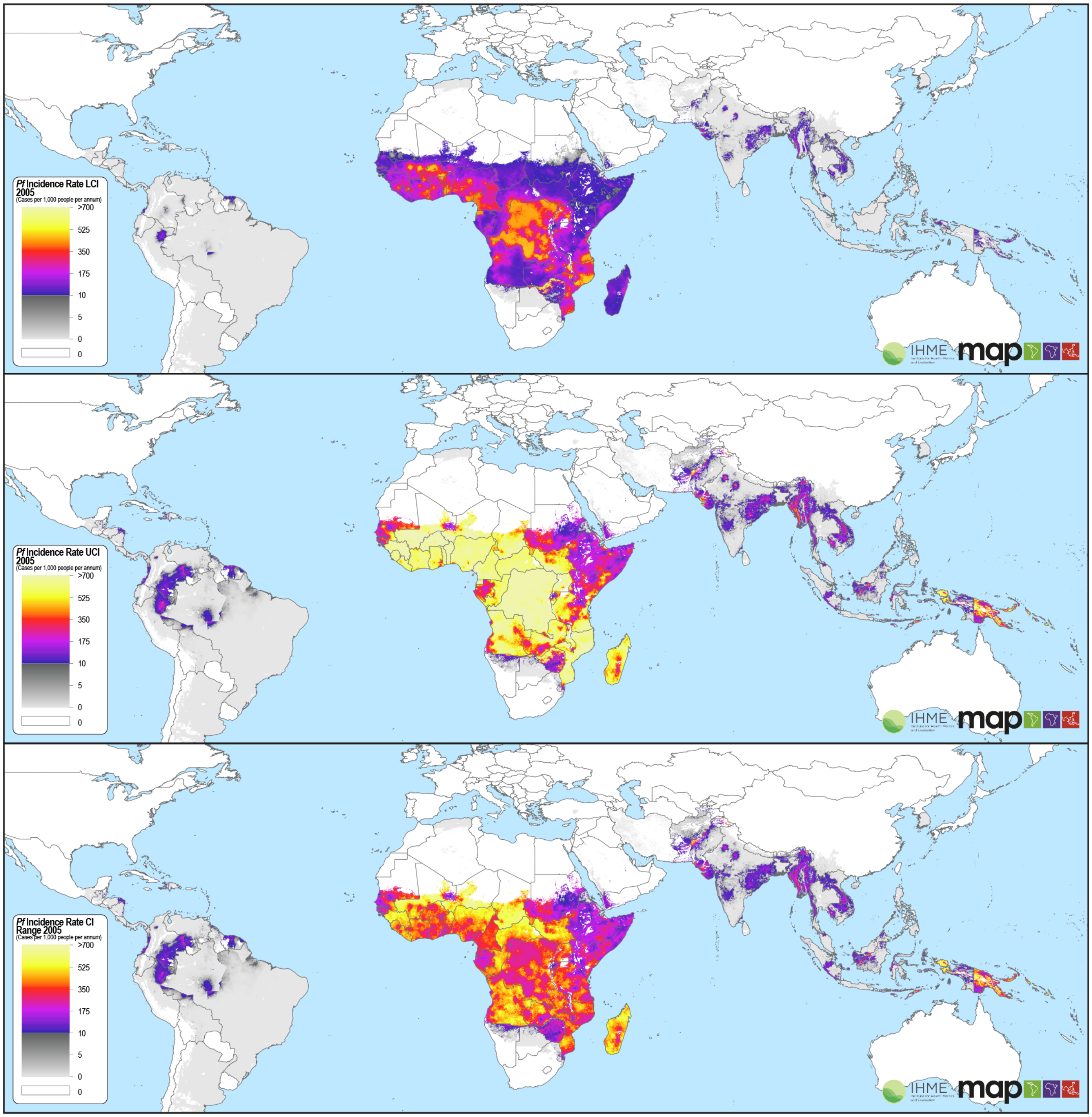

Figure S39: Lower and upper credible intervals (CIs) as well as the range for *Pf* incidence in 2005.

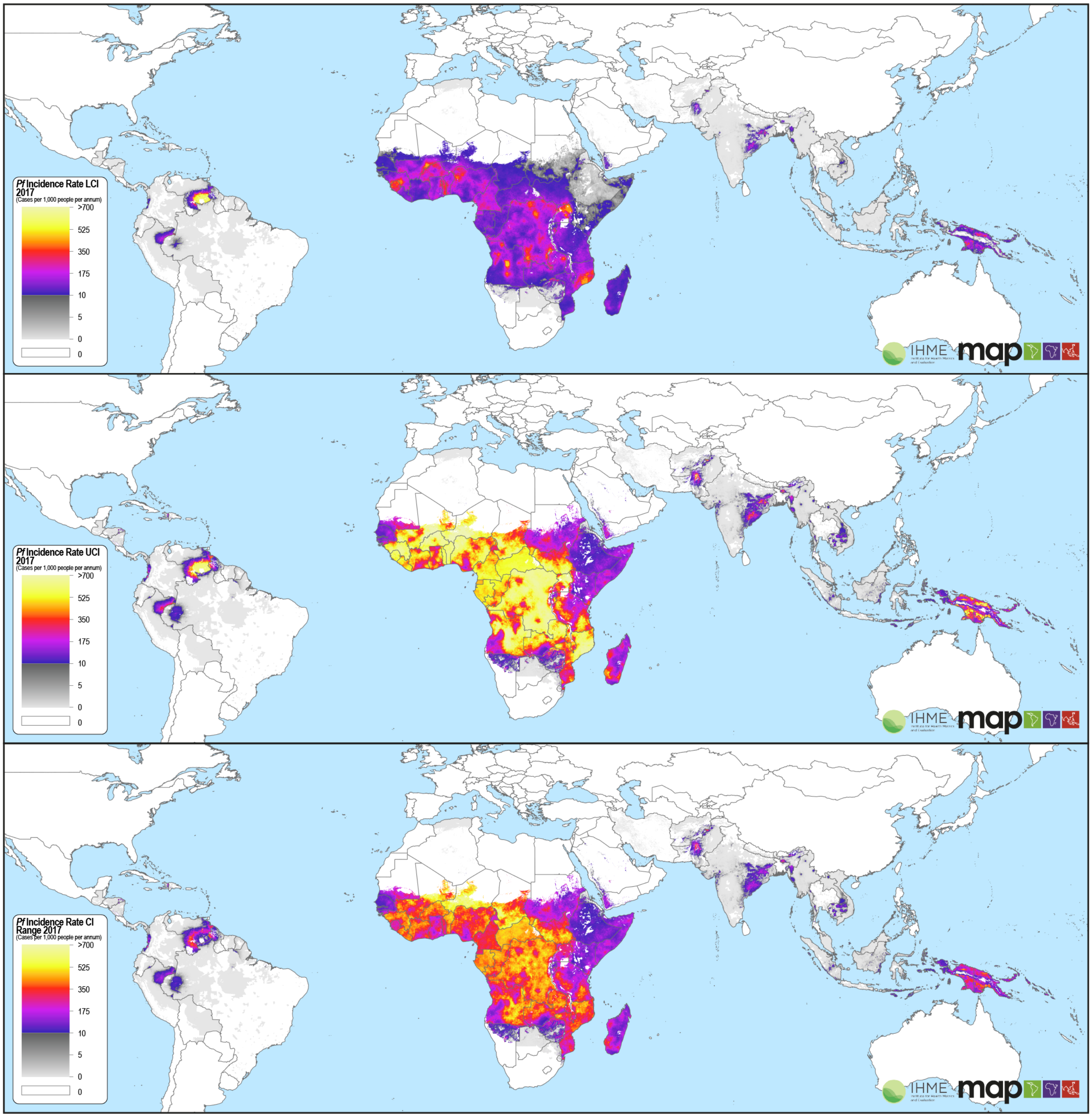

Figure S40: Lower and upper credible intervals (CIs) as well as the range for *Pf* incidence in 2017.

2 Mortality

2.1 Data

Data for *P. falciparum* mortality models included vital registration, verbal autopsy, and surveillance data obtained from the IHME Cause of Death (CoD) Database.[45] The CoD database provides cause-specific mortality information at both site and administration unit levels. Variability in the type and abundance of CoD and related data meant that two distinct approaches were developed to estimate malaria mortality due to *P. falciparum* inside Africa and *P. falciparum* outside Africa.

2.2 Methods

2.2.1 Mortality in Africa

2.2.1.1 Effective treatment

Effective treatment was estimated by estimating the usage of three antimalarial drugs: chloroquine (CQ), sulfadoxine-pyrimethamine (SP) and artemisinin combination therapy (ACT).

This was combined with estimates of drug resistance to give a spatiotemporal cube of effective treatment rate. To disaggregate into GBD age-bins, we separately ran a traditional national-level CODEm model with covariates: prevalence of *P. falciparum* in 2–10 age group, *P. falciparum* incidence rate, years of education, access to effective antimalarial drugs, and health system access. The effective treatment rate was combined with the incidence rate cube to derive a third cube estimating the incidence of untreated cases. Details of the models can be found in the appendix to Gething *et al.*[23]

2.2.1.2 Case fatality rate

For each site-year for which CoD malaria cause fraction data were available we (i) estimated a site-year specific malaria mortality rate, as the product of malaria cause fraction and all-cause mortality rate (with the latter drawn from national-level values); (ii) divided the malaria mortality rate by the site-year specific estimate of untreated malaria incidence rate to estimate a site-year specific case fatality rate (CFR) amongst untreated malaria cases. These derived site-year specific CFR values were then used in a mixed-effects regression model to estimate pixel-year CFR for each 5km × 5km grid cell. The covariates used in the model were the log of country-year all-cause mortality, night-time lights, accessibility and fractional land-cover classes, and study-specific age and sex, with the location of each study site included as a national-level random effect. Data were weighted by sample size (i.e. the number of all-cause deaths observed in each study site-year). Further details can be found in previous publications.[23]

2.2.1.3 Cartographic mortality cube

To estimate the fatal burden of *P. falciparum* malaria in Africa, we used epidemiologic measures of non-fatal malaria burden as described in section "Africa prevalence model". Pixel-year predictions of CFR were then multiplied by the corresponding untreated incidence rate cube to yield a pixel-year mortality rate estimate, which was then multiplied by pixel-year population to compute pixel-year malaria death estimates. These were then aggregated to yield the required GBD national or subnational death estimates.

2.2.2 Mortality in surveillance countries

In locations modelled using the surveillance approach, we used a traditional cause of death ensemble model (CODEm),[45] closely mirroring that used in GBD 2015. The model included the following covariates: prevalence of *P. falciparum* of 2–10 age group, *P. falciparum* incidence rate, years of education, access to effective antimalarial drugs, and health system access.

2.2.3 Raking death estimates to match GBD results

Malaria mortality estimates both inside and outside of Africa were aligned to the malaria death results from the GBD2017 by raking (i.e. linearly scaling up or down) them over our modelled mortality surfaces. This process preserved the spatial heterogeneity from earlier models while taking advantage of the GBD approach for balancing all causes of death within a single model. In brief, the GBD estimates were generated at the administrative level by taking the mortality estimates from all causes of deaths in the study (including those for malaria that we produced), and then applying the CodCorrect model to ensure that the sum of deaths for all causes matched the modelled total from an all-cause envelope. This approach reflects a key principle of the GBD to ensure internal consistency in mortality estimates across causes and within the all-cause envelope. One consequence is that the original modelled link between malaria case incidence and malaria mortality is decoupled to enable the latter to be adjusted where required by the CodCorrect process. This, in turn, caused some areas with low-malaria burden to have implied CFRs that are outside expected ranges (i.e., implausible mortality given the modelled incidence). This was considered preferable than the possible alternatives of (a) omitting the CodCorrect step or (b) making post-hoc adjustments to underlying case incidence estimates to preserve originally modelled CFRs.

2.3 Results

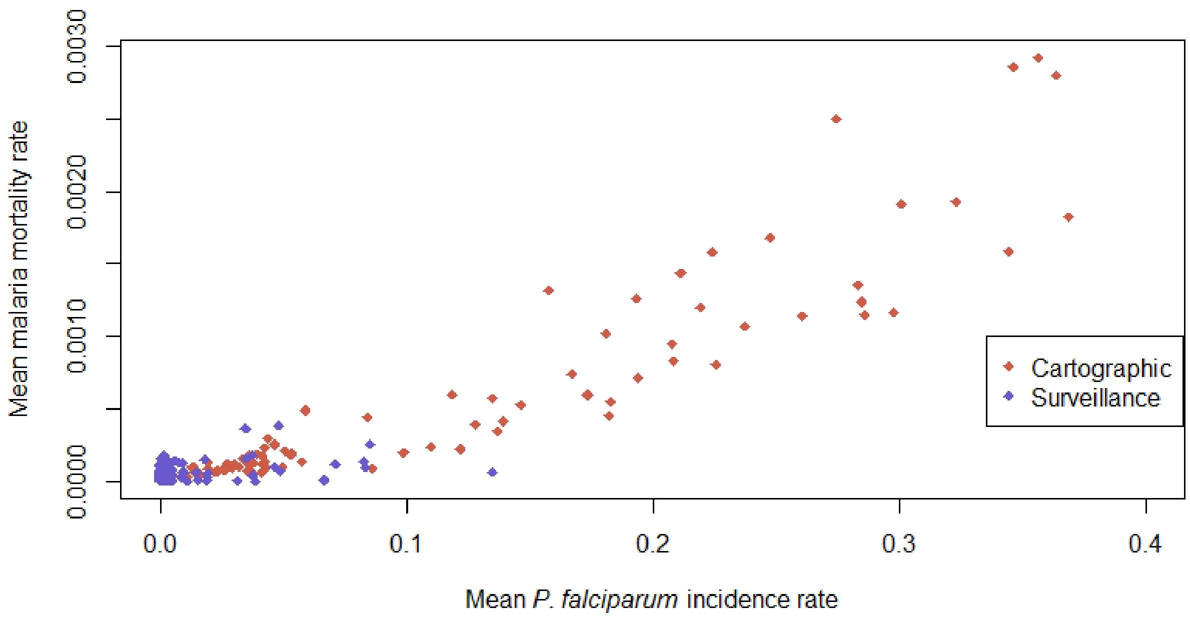

Figure S41: Plot of the mean malaria mortality rate against the mean *P. falciparum* incidence rate.

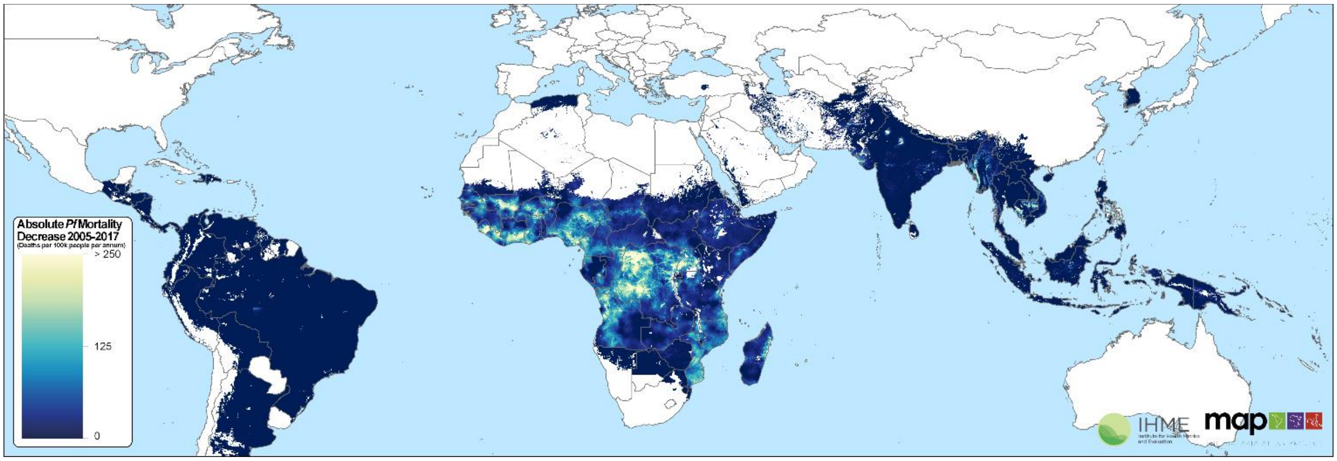

Figure S42: Absolute mortality rate decrease 2005-2017.

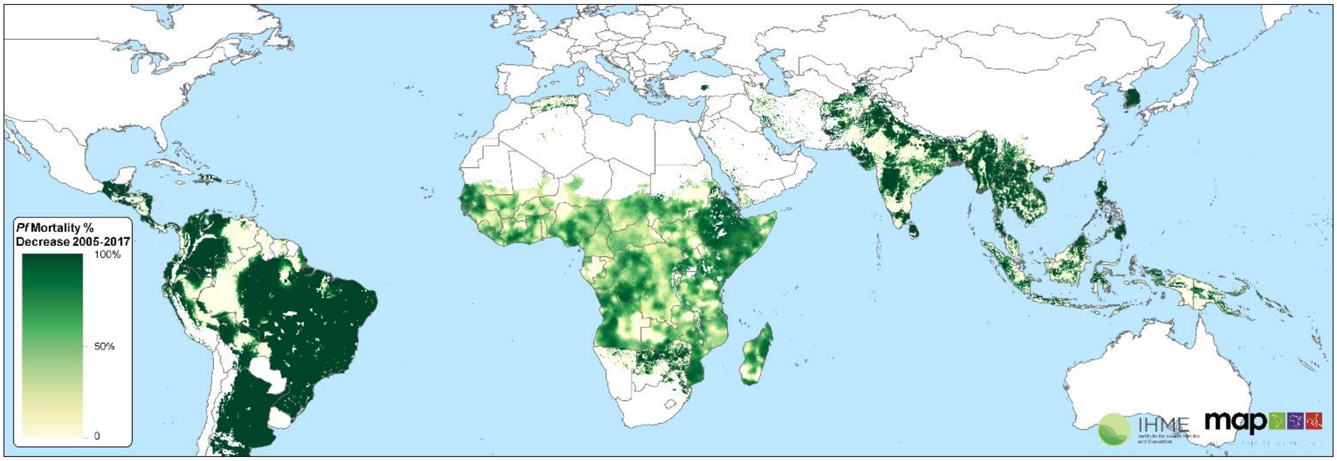

Figure S43: Relative mortality rate decrease 2005-2017.

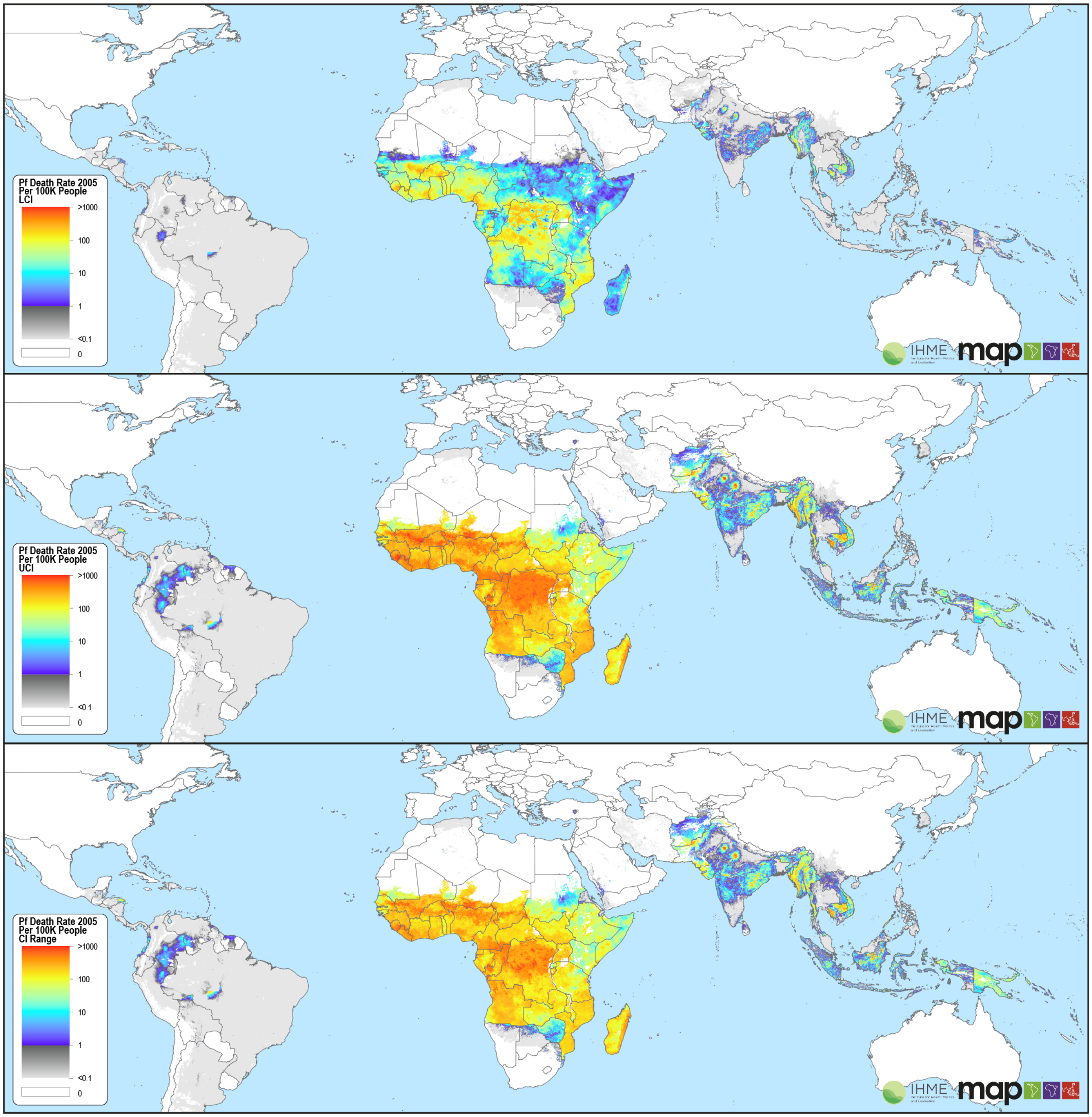

Figure S44: Lower and upper credible intervals (CIs) as well as the range for *Pf* mortality in 2005.

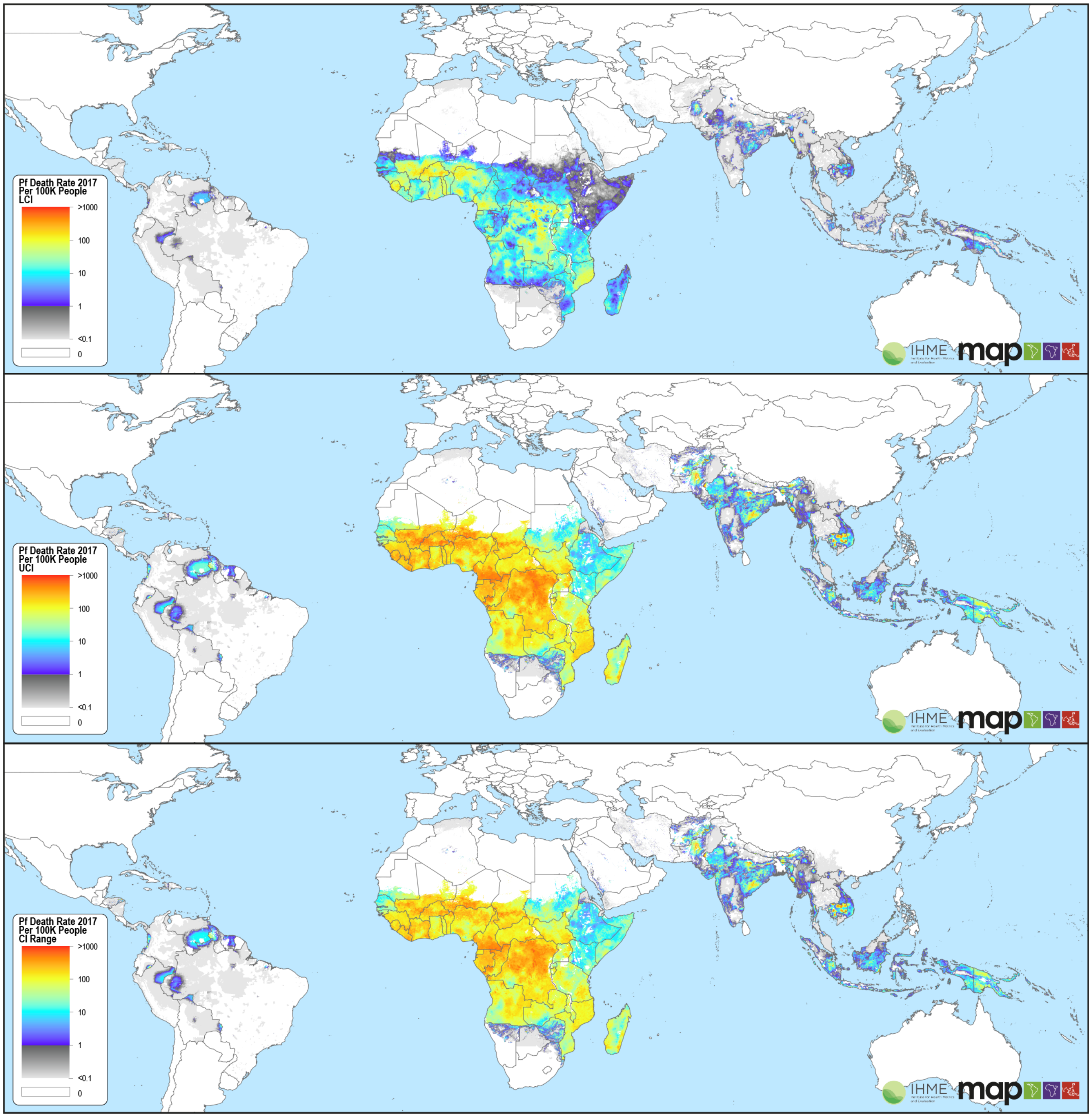

Figure S45: Lower and upper credible intervals (CIs) as well as the range for *Pf* mortality in 2017.

3 Schematic diagrams

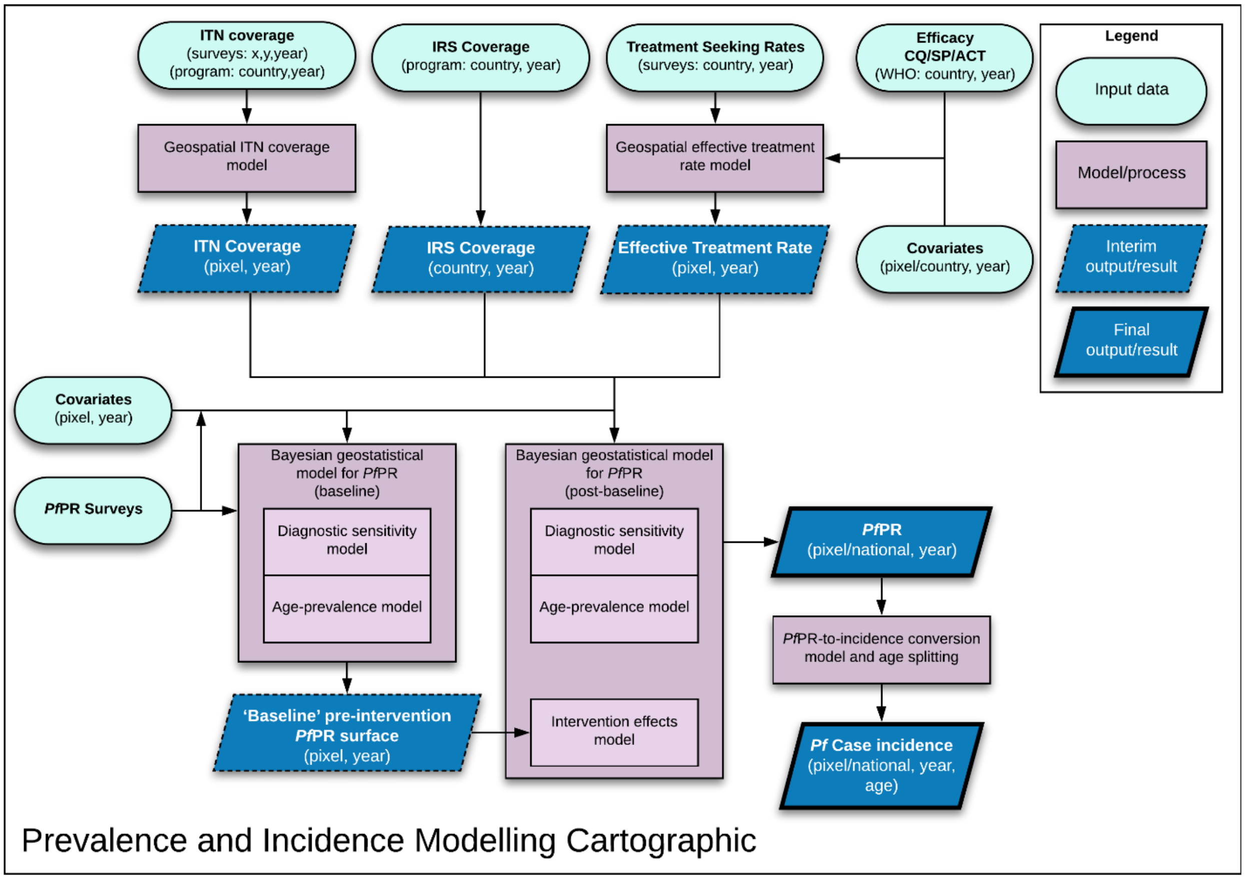

Figure S46: Flowchart of the prevalence and incidence modelling process for cartographic countries.

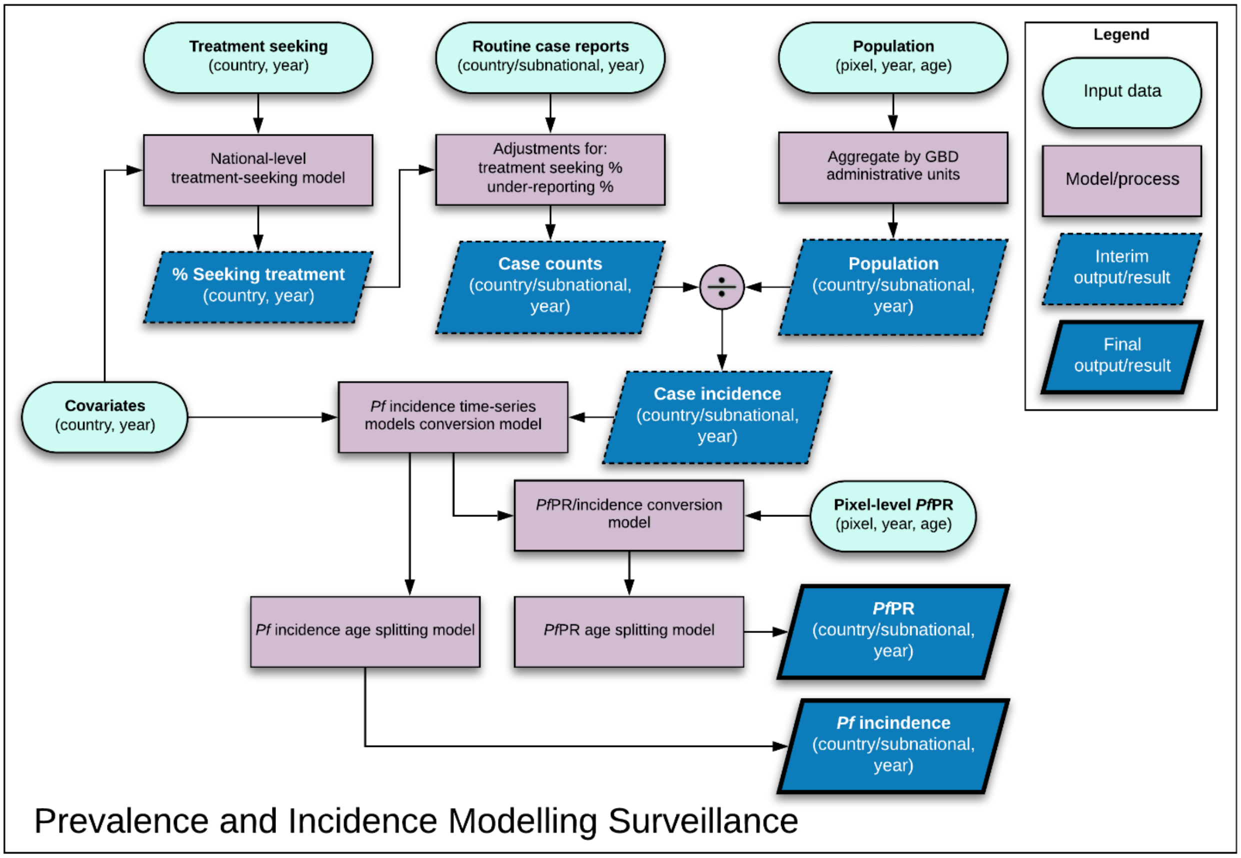

Figure S47: Flowchart of the prevalence and incidence modelling process for surveillance countries.

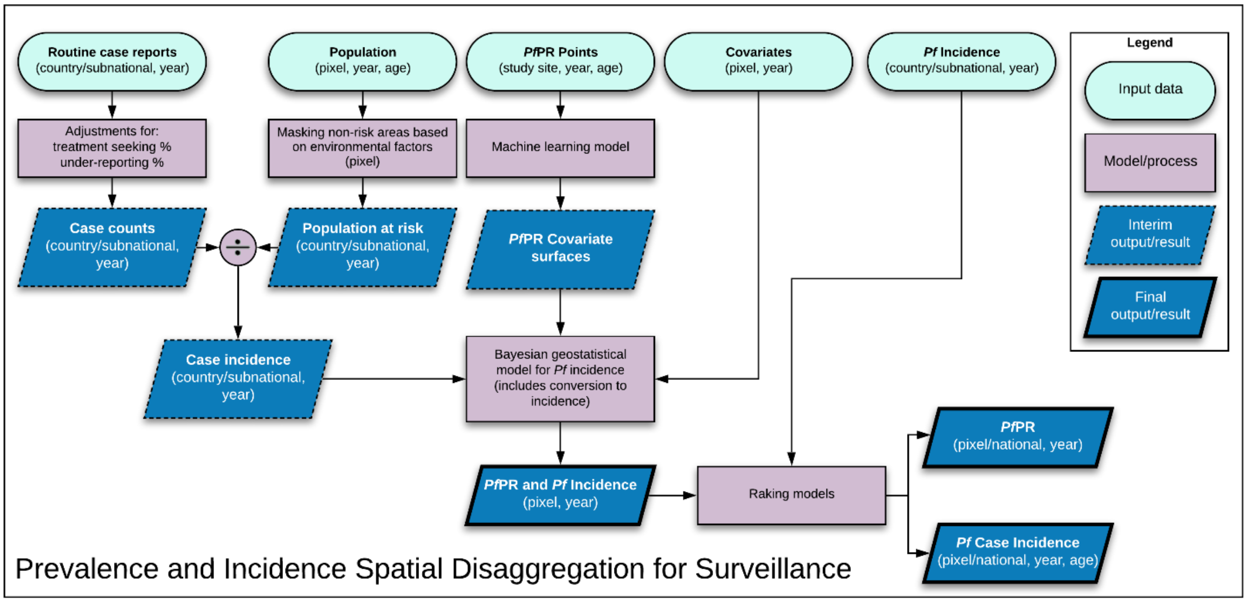

Figure S48: Flowchart of the spatial disaggregation of prevalence and incidence estimates for surveillance countries.

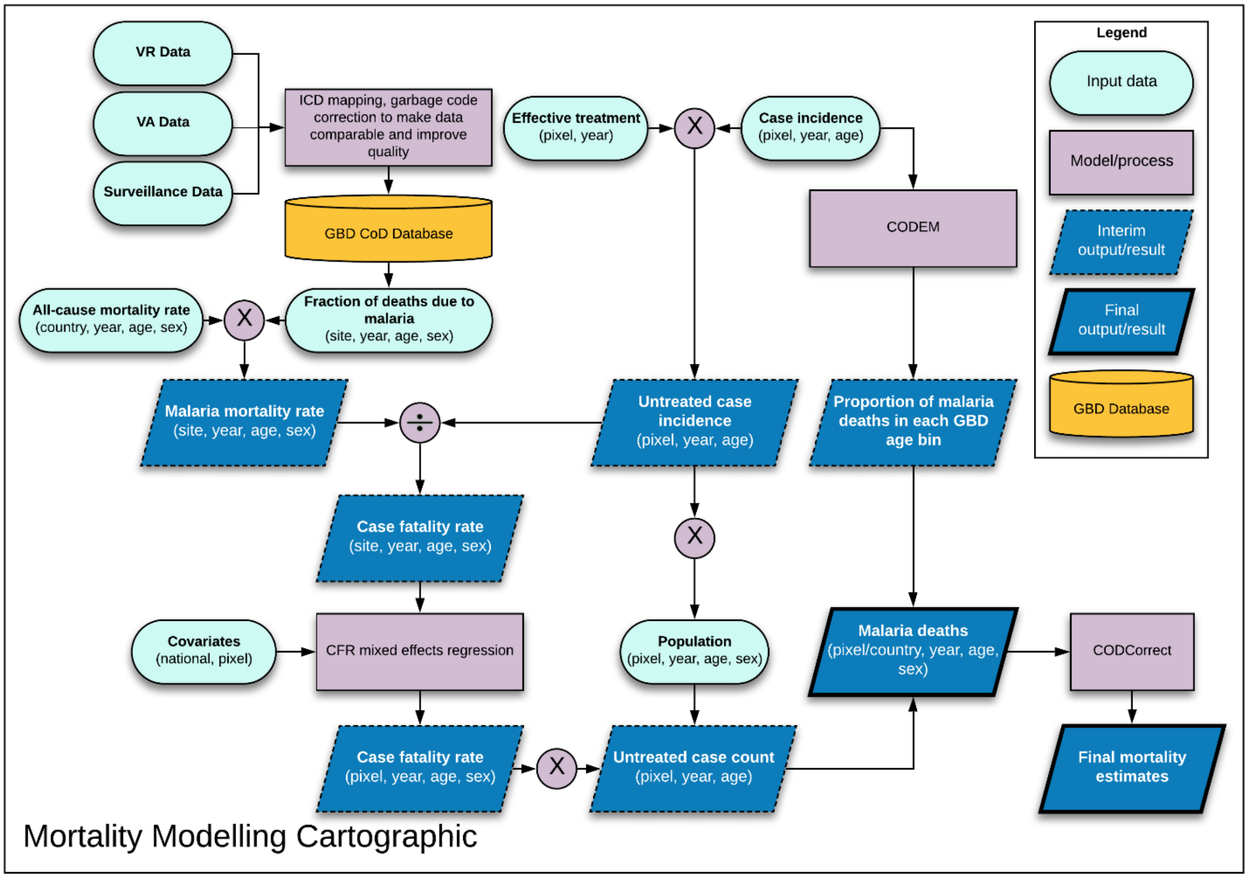

Figure S49: Flowchart of the mortality modelling process for cartographic countries.

4 Extended figures and table

Year	Region	*Pf-Free*	Hypo	Meso	Hyper	Holo
2005	Global	0.515 (0.515-0.515)	0.411 (0.409-0.413)	0.047 (0.044-0.051)	0.018 (0.015-0.020)	0.010 (0.008-0.012)
2017	Global	0.591 (0.591-0.592)	0.344 (0.341-0.349)	0.057 (0.052-0.060)	0.007 (0.005-0.009)	0.001 (0.000-0.002)
2005	Latin America and Caribbean	0.353 (0.353-0.353)	0.647 (0.646-0.647)	0.001 (0.000-0.001)	-	-
2017	Latin America and Caribbean	0.665 (0.665-0.665)	0.335 (0.335-0.335)	0.000 (0.000-0.001)	-	-
2005	North Africa and Middle East	0.701 (0.699-0.710)	0.294 (0.286-0.298)	0.005 (0.003-0.008)	-	-
2017	North Africa and Middle East	0.769 (0.762-0.776)	0.228 (0.221-0.237)	0.003 (0.001-0.007)	-	-
2005	South Asia	0.018 (0.018-0.018)	0.974 (0.971-0.975)	0.007 (0.006-0.010)	-	-
2017	South Asia	0.179 (0.179-0.179)	0.817 (0.816-0.817)	0.004 (0.004-0.005)	-	-
2005	Southeast Asia, East Asia, and Oceania	0.718 (0.718-0.718)	0.280 (0.280-0.280)	0.002 (0.001-0.002)	-	-
2017	Southeast Asia, East Asia, and Oceania	0.822 (0.822-0.822)	0.176 (0.176-0.177)	0.001 (0.001-0.001)	-	-
2005	Sub-Saharan Africa	0.102 (0.102-0.102)	0.262 (0.244-0.285)	0.391 (0.363-0.422)	0.157 (0.135-0.178)	0.088 (0.073-0.106)
2017	Sub-Saharan Africa	0.090 (0.091-0.091)	0.449 (0.425-0.481)	0.404 (0.369-0.428)	0.050 (0.040-0.063)	0.006 (0.003-0.011)

Table S10: **Global and regional proportions of population within *P. falciparum* endemicity classes in 2005 and 2016.** Hypo- (*Pf*PR >0·0 – 0·1); Meso- (>0·1 – 0·5); Hyper- (>0·5 – 0·75); and Holo-Endemic (>0·75 – 1).

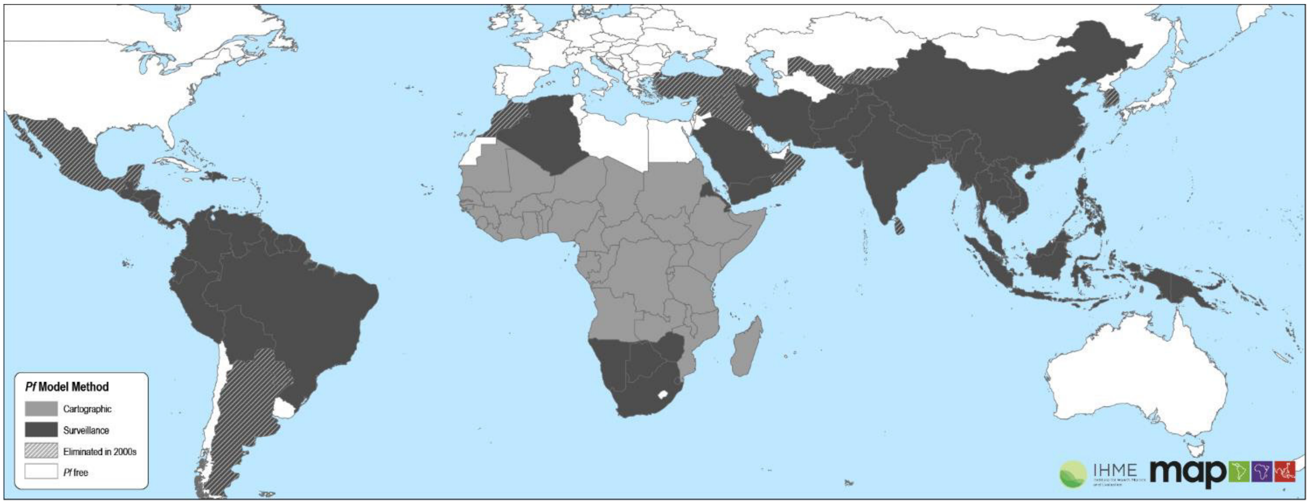

Figure S50: **Categorizing modeling strategies for *Plasmodium falciparum* endemic countries in 2000-2017**. Malaria estimates were generated with the cartographic method for countries colored light gray and with the surveillance-based method for countries colored dark gray. Countries colored as white have either never been malaria-endemic or achieved malaria-free status prior to the year 2000.

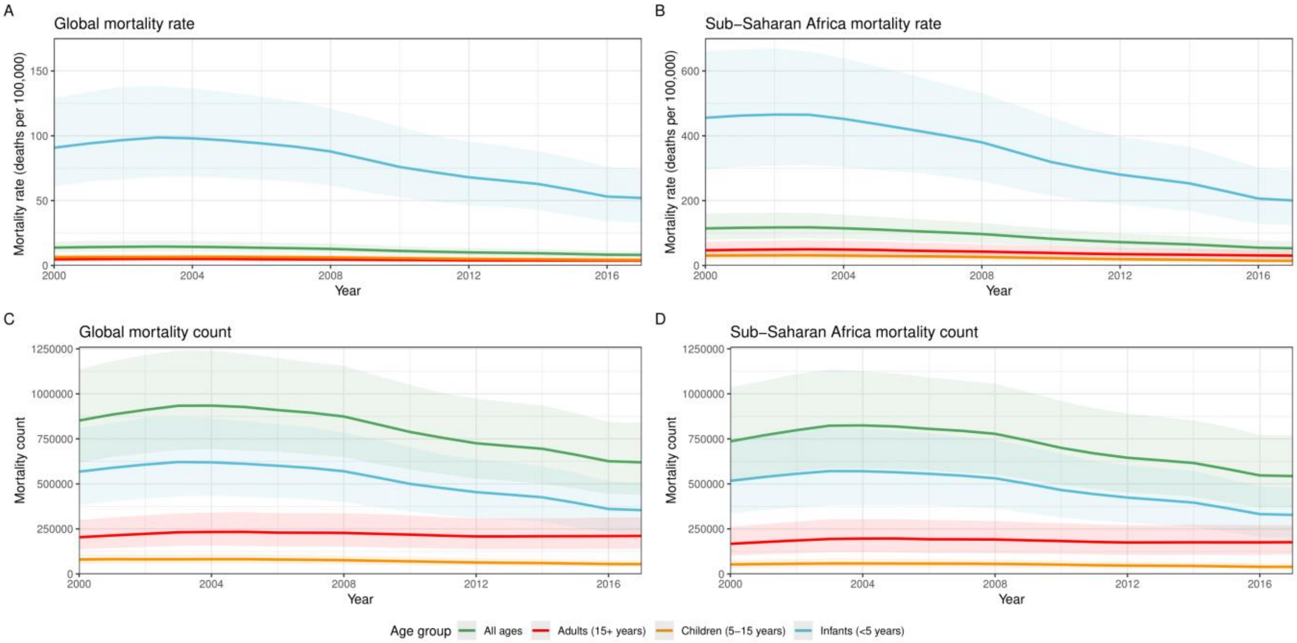

Figure S51: ***Plasmodium falciparum* mortality rate (A and B) and count (C and D) presented with uncertainty intervals, stratified by age group, globally and for sub-Saharan Africa from 2000-2017.** 95% uncertainty intervals shown via the corresponding colored bands behind the mean lines. Rates were calculated using the total population in each age group in all endemic countries.

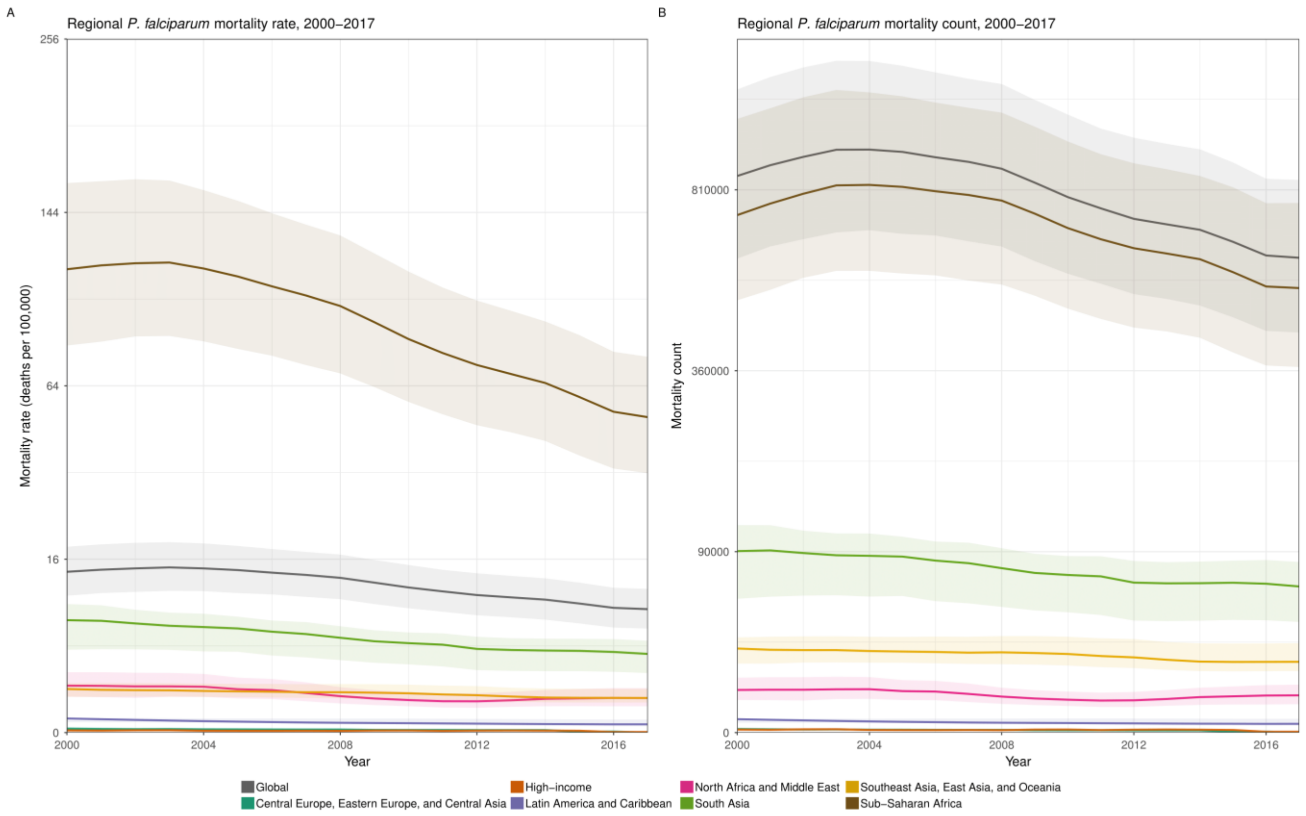

Figure S52: **The regional distribution of *Plasmodium falciparum* mortality rate (A) and count (B).** To show trends across regions with such different endemicity levels the y-axis is scaled using the square root of incidence rate (per 100,000 individuals) and count (in millions of cases) for A and B, respectively. 95% uncertainty intervals shown via the corresponding colored bands behind the mean lines. Rates were calculated using the total population in all endemic countries within each region.

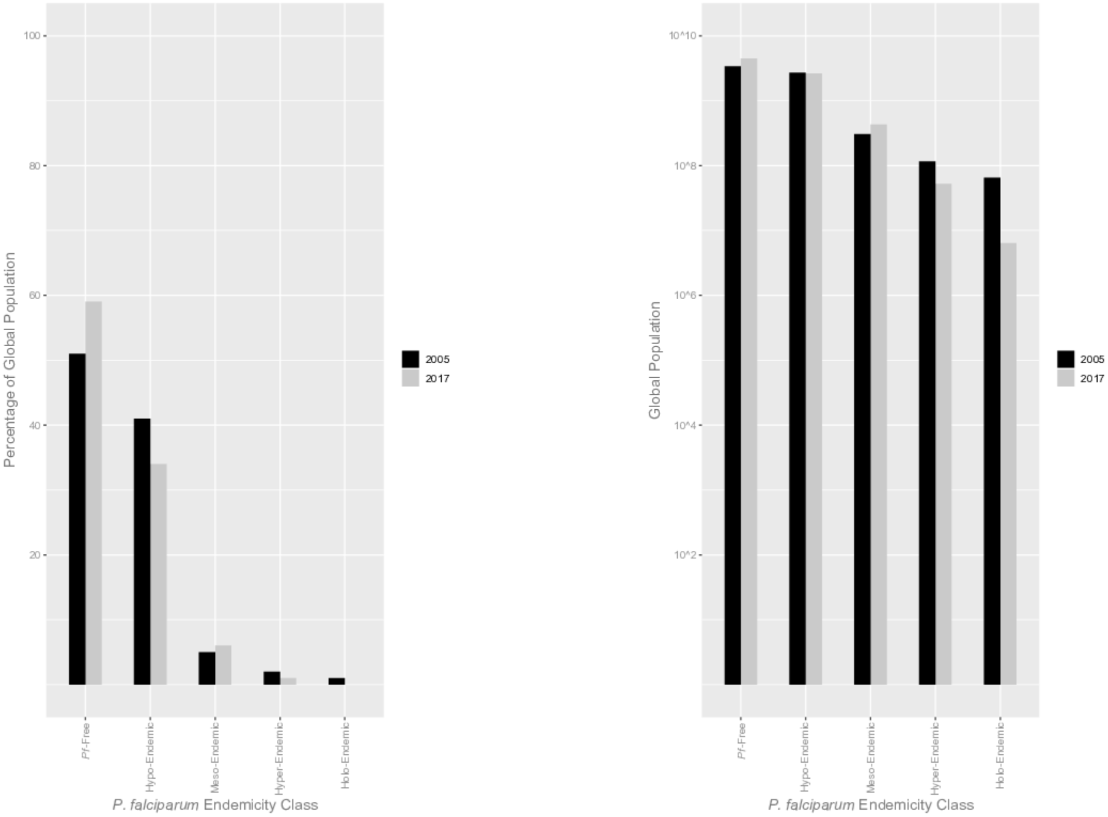

Figure S53: **The distribution of global population in 2005 and 2017 relative to *Plasmodium falciparum* risk shown as a percentage of global population (left) and in total population on a log base 10 scale (right).** *P. falciparum* risk is stratified according to standard endemic classes: Hypo- (*Pf*PR >0·0 – 0·1); Meso- (>0·1 – 0·5); Hyper- (>0·5 – 0·75); and Holo-endemic (>0·75 – 1).

5 GATHER compliance

5.1 Checklist

Item No.	Checklist item	Reference
	**Objectives and funding**	
**1**	Define the indicator(s), populations (including age, sex, and geographic entities), and time period(s) for which estimates were made.	Main text (Introduction, page 4)
**2**	List the funding sources for the work.	Main text (funding statement)
	**Data Inputs**	
	From multiple sources that are synthesized as part of the study:	
**3**	Describe how the data were identified and how the data were accessed.	Main text (pages 5 - 6), Supplementary Information (sections 1.1 and 2.1)
**4**	Specify the inclusion and exclusion criteria. Identify all ad-hoc exclusions.	Supplementary Information (section 1.1)
**5**	Provide information on all included data sources and their main characteristics. For each data source used, report reference information or contact name/institution, population represented, data collection method, year(s) of data collection, sex and age range, diagnostic criteria or measurement method, and sample size, as relevant.	Supplementary Information (sections 1.1 and 2.1) and online data citation tools from http: //ghdx.healthdata.org/gbd-2017 and https://map.ox.ac.uk/gather-compliance/
**6**	Identify and describe any categories of input data that have potentially important biases (e.g., based on characteristics listed in item 5).	Main text (page 4)
	Which contribute to the analysis but were not synthesized as part of the study:	
**7**	Describe and give sources for any other data inputs.	Supplementary Information (section 1.1)
	For all data inputs:	
**8**	Provide all data inputs in a file format from which data can be efficiently extracted (e.g., a spreadsheet rather than a PDF), including all relevant meta-data listed in item 5. For any data inputs that cannot be shared because of ethical or legal reasons, such as third-party ownership, provide a contact name or the name of the institution that retains the right to the data.	http://ghdx.healthdata.org/gbd-2017 and https: //map.ox.ac.uk/gather-compliance/
	**Data analysis**	
**9**	Provide a conceptual overview of the data analysis method. A diagram may be helpful.	Main text (Methods overview)

Item No.	Checklist item	Reference
**10**	Provide a detailed description of all steps of the analysis, including mathematical formulae. This description should cover, as relevant, data cleaning, data pre-processing, data adjustments and weighting of data sources, and mathematical or statistical model(s).	Supplementary Information (sections 1.2 and 2.2)
**11**	Describe how candidate models were evaluated and how the final model(s) were selected.	Main text (page 5-7) and Supplementary Information (sections 1.2 and 2.2)
**12**	Provide the results of an evaluation of model performance, if done, as well as the results of any relevant sensitivity analysis.	Supplementary Information (sections 1.2 and 2.2)
**13**	Describe methods for calculating uncertainty of the estimates. State which sources of uncertainty were, and were not, accounted for in the uncertainty analysis.	Main text (page 5) and Supplementary Information (sections 1.2 and 2.2)
**14**	State how analytic or statistical source code used to generate estimates can be accessed.	Main text (page 7)
	**Results and Discussion**	
**15**	Provide published estimates in a file format from which data can be efficiently extracted.	Available from www.map.ox.ac.uk/malaria-burden
**16**	Report a quantitative measure of the uncertainty of the estimates (e.g. uncertainty intervals).	Main text (Results, Table 1, Figures 2 – 5)
**17**	Interpret results in light of existing evidence. If updating a previous set of estimates, describe the reasons for changes in estimates.	Research in context, Main text (Discussion)
**18**	Discuss limitations of the estimates. Include a discussion of any modelling assumptions or data limitations that affect interpretation of the estimates.	Main text (Discussion)

References

1 NASA LP DAAC. Land Cover Type Yearly L3 Global 500m SIN Grid. Version 051. 2013. https://lpdaac.usgs.gov.

2 NASA LP DAAC. Nadir BRDF- Adjusted Reflectance Reflectance 16-Day L3 Global 1km. Version 005. 2015. https://lpdaac.usgs.gov.

3 NASA LP DAAC. Land Surface Temperature and Emissivity 8-Day L3 Global 1km. Version 005. 2015. https://lpdaac.usgs.gov.

4 Hijmans RJ, Cameron SE, Parra JL, Jones PG, Jarvis A. Very high resolution interpolated climate surfaces for global land areas. *International Journal of Climatology* 2005; **25**: 1965–78.

5 Weiss D, Nelson A, Gibson H *et al.* A global map of travel time to cities to assess inequalities in accessibility in 2015. *Nature* 2018; **553**: 333.

6 NASA LP DAAC. SRTMGL3S: NASA Shuttle Radar Topography Mission Global 3 arc second sub-sampled. Version 003. 2013. https://lpdaac.usgs.gov.

7 Zomer RJ, Trabucco A, Bossio DA, Verchot LV. Climate change mitigation: A spatial analysis of global land suitability for clean development mechanism afforestation and reforestation. *Agriculture, Ecosystems & Environment* 2008; **126**: 67–80.

8 Weiss DJ, Atkinson PM, Bhatt S, Mappin B, Hay SI, Gething PW. An effective approach for gap-filling continental scale remotely sensed time-series. *ISPRS Journal of Photogrammetry and Remote Sensing* 2014; **98**: 106–18.

9 NASA. Gridded Population of the World (GPW), v4. http://sedac.ciesin.columbia.edu/data/collection/gpw-v4.

10 Tatem AJ. WorldPop, open data for spatial demography. *Scientific data* 2017; **4**: 170004–4.

11 Gething PW, Patil AP, Smith DL *et al.* A new world malaria map: *Plasmodium falciparum* endemicity in 2010. *Malaria journal* 2011; **10**: 378.

12 GADM database of Global Administrative Areas, version 2.8. 2015. http://www.gadm.org.

13 FAO. The Global Administrative Unit Layers (GAUL). 2015. http://www.fao.org/geonetwork/srv/en/main.home#boundaries.

14 Bhatt S, Weiss D, Cameron E *et al.* The effect of malaria control on *Plasmodium falciparum* in Africa between 2000 and 2015. *Nature* 2015; **526**: 207.

15 Guerra CA, Hay SI, Lucioparedes LS *et al.* Assembling a global database of malaria parasite prevalence for the Malaria Atlas Project. *Malaria journal* 2007; **6**: 17.

16 The DHS Program. The DHS Program. 2017. http://www.dhsprogram.com/.

17 Rutstein SO, Rojas G. Guide to DHS statistics. *Calverton, MD: ORC Macro* 2006.

18 World Health Organization. World malaria report 2015. Geneva, 2015.

19 Lumley T. Analysis of complex survey samples. *Journal of Statistical Software* 2004; **9**: 1–19.

20 The World Bank. Data: Indicators. http://data.worldbank.org/indicator/all.

21 Battle KE, Bisanzio D, Gibson HS *et al.* Treatment-seeking rates in malaria endemic countries. *Malaria journal* 2016; **15**: 20.

22 Cameron E, Battle KE, Bhatt S *et al.* Defining the relationship between infection prevalence and clinical incidence of *plasmodium falciparum* malaria. *Nature communications* 2015; **6**.

23 Gething PW, Casey DC, Weiss DJ *et al.* Mapping *Plasmodium falciparum* mortality in Africa between 1990 and 2015. *New England Journal of Medicine* 2016; **375**: 2435–45.

24 Rasmussen CE. Gaussian processes in machine learning. In: Advanced lectures on machine learning. Springer, 2004: 63–71.

25 Roberts S, Osborne M, Ebden M, Reece S, Gibson N, Aigrain S. Gaussian processes for time-series modelling. *Phil Trans R Soc A* 2013; **371**: 20110550.

26 R Core Team. R: A language and environment for statistical computing. Vienna, Austria: R Foundation for Statistical Computing, 2017 https://www.R-project.org/.

27 MacDonald B, Ranjan P, Chipman H. GPfit: An R package for fitting a Gaussian process model to deterministic simulator outputs. *Journal of Statistical Software* 2015; **64**: 1–23.

28 Ranjan P, Haynes R, Karsten R. A computationally stable approach to Gaussian process interpolation of deterministic computer simulation data. *Technometrics* 2011; **53**: 366–78.

29 Carey V, Wang Y-G. Mixed-effects models in S and S-PLUS. 2001.

30 Cibulskis RE, Aregawi M, Williams R, Otten M, Dye C. Worldwide incidence of malaria in 2009: Estimates, time trends, and a critique of methods. *PLoS medicine* 2011; **8**: e1001142.

31 World Health Organization. World malaria report 2012. Geneva, 2012.

32 World Health Organization. World malaria report 2016. Geneva, 2016.

33 Cibulskis R. 2017.

34 World Health Organization. World malaria report 2017. Geneva, 2017.

35 Kristensen K, Nielsen A, Berg CW, Skaug H, Bell BM. TMB: Automatic differentiation and Laplace approximation. *Journal of Statistical Software* 2016; **70**: 1–21.

36 Kuhn M, Wing J, Weston S *et al.* caret: Classification and Regression Training. 2017 https://CRAN.R-project.org/package=caret.

37 Zou H, Hastie T. elasticnet: Elastic-Net for Sparse Estimation and Sparse PCA. 2012 https://CRAN.R-project.org/package=elasticnet.

38 Liaw A, Wiener M. Classification and Regression by randomForest. *R News* 2002; **2**: 18–22.

39 Venables WN, Ripley BD. Modern Applied Statistics with S, Fourth. New York: Springer, 2002 http://www.stats.ox.ac.uk/pub/MASS4.

40 Greenwell B, Boehmke B, Cunningham J *et al.* gbm: Generalized Boosted Regression Models. 2017 https://CRAN.R-project.org/package=gbm.

41 GBD 2017 Disease and Injury Incidence and Prevalence Collaborators. Global, regional, and national incidence, prevalence, and years lived with disability for 355 diseases and injuries for 195 countries, 1990–2017: a systematic analysis for the Global Burden of Disease Study 2017. *The Lancet* In press.

42 Sturrock HJ, Cohen JM, Keil P *et al.* Fine-scale malaria risk mapping from routine aggregated case data. *Malaria journal* 2014; **13**: 421.

43 Lindgren F, Rue H, Lindström J. An explicit link between Gaussian fields and Gaussian Markov random fields: The stochastic partial differential equation approach. *J R Stat Soc Ser B Stat Methodol* 2011; **73**.
